# Long noncoding RNA MARL regulates antiviral responses through suppression miR-122-dependent MAVS downregulation in lower vertebrates

**DOI:** 10.1371/journal.ppat.1008670

**Published:** 2020-07-17

**Authors:** Qing Chu, Tianjun Xu, Weiwei Zheng, Renjie Chang, Lei Zhang

**Affiliations:** 1 Laboratory of Fish Molecular Immunology, College of Fisheries and Life Science, Shanghai Ocean University, Shanghai, China; 2 Laboratory of Marine Biology and Biotechnology, Qingdao National Laboratory for Marine Science and Technology, Qingdao, China; 3 National Pathogen Collection Center for Aquatic Animals, Shanghai Ocean University, Shanghai, China; 4 Key Laboratory of Exploration and Utilization of Aquatic Genetic Resources (Shanghai Ocean University), Ministry of Education, Shanghai, China; University of Utah, UNITED STATES

## Abstract

Increasing evidence suggests important roles for long noncoding RNAs (lncRNAs) as new gene modulators involved in various biological processes. However, the function roles of lncRNAs in lower vertebrates are still unknown. Here, we firstly identify a lncRNA, named MAVS antiviral-related lncRNA (MARL), as a key regulator for antiviral immunity in teleost fish. The results indicate that fish MAVS play essential roles in host antiviral responses and inhibition of *Siniperca chuatsi rhabdovirus* (SCRV) replication. miR-122 reduces MAVS expression and suppress MAVS-mediated antiviral responses, which may help viruses evade host antiviral responses. Further, MARL functions as a competing endogenous RNA (ceRNA) for miR-122 to control protein abundance of MAVS, thereby inhibiting SCRV replication and promoting antiviral responses. Our data not only shed new light on understanding the function role of lncRNA in biological processes in lower vertebrates, but confirmed the hypothesis that ceRNA regulatory networks exist widely in vertebrates.

## Author summary

Increasing evidence indicates that lncRNAs participate in the regulation of various biological processes, especially innate and adaptive immunity. However, the relationship between lncRNAs and host antiviral responses remains largely unknown, particularly in lower vertebrates. Our results provided the first direct evidence that a lncRNA, termed MAVS antiviral-related lncRNA (MARL), acts as a key regulator for antiviral immunity in lower vertebrates. lncRNAs have been identified to function as competing endogenous RNAs (ceRNAs) and cross-talk with mRNAs by competing shared for miRNAs. Such ceRNAs regulate the distribution of miRNA molecules on their targets and thereby apply an additional level of post-transcriptional regulation. In our study, MARL functions as a ceRNA for miR-122 to control protein abundance of fish MAVS, thereby inhibiting virus replication and promoting antiviral responses. This is the first study to demonstrate ceRNA regulatory networks existing in lower vertebrates, which can provide new insights into understanding the effects of lncRNAs on host-virus interactions.

## Introduction

Viral infection triggers host immune responses to rapidly detect and eliminate invading viruses, thereby survival of the host. In host cells, detection of viral infection involves Toll-like receptors (TLRs) and retinoic acid-inducible gene-I (RIG-I)-like receptors (RLRs) that initiate signaling cascades to coordinately lead to the production of type-I interferons (IFNs) [1, 2]. Unlike the TLR receptors mediating antiviral responses, RLRs receptors RIG-I and MDA5 function as cytoplasmic sensors for viral RNA recognition [3]. Both RIG-I and MDA5 contain a C-terminal DExD/H box RNA helicase domain that directly interacts with viral RNAs and two N-terminal caspase activation and recruitment domains (CARDs) that promote the CARD-mediated downstream signaling cascade to activate the essential adaptor mitochondrial antiviral signaling protein (MAVS; also termed IPS-1/Cardif/VISA) [3–5]. MAVS then recruits the TBK1 and IKK complex to activate transcription factors IRF3/IRF7 and NF-κB respectively, which finally orchestrate the IFN antiviral response and suppress virus replication [5, 6]. Excessive activation of MAVS-mediated antiviral signaling will disrupt immune homeostasis, and further it may induce autoimmune and inflammatory diseases. Therefore, the regulation mechanisms of MAVS-mediated signaling need be extensively investigated.

MicroRNAs (miRNAs) are endogenous short RNA molecules with 22–24 nucleotides that act as regulators of gene expression by inhibiting mRNA translation or promoting mRNA degradation. miRNAs are implicated in the regulation of diverse biological processes, including development, apoptosis, proliferation, and differentiation [7, 8]. Recently, mounting evidence demonstrates that miRNAs play pivotal roles in regulating virus-induced immune response among different vertebrate species. In mammals, several miRNAs, including miR-15b, miR-155, miR-19b-3p, miR-29b, and miR-301a have been reported to involve in regulating inflammatory response upon Japanese encephalitis virus (JEV) infection [9–13]. In birds, microRNA-23b has been shown to target IRF1 and further down-regulation the antiviral responses in avian leukosis virus subgroup J infection [14]. Most recently, in lower vertebrates, studies have reported that fish miRNAs, such as miR-210 and miR-3570, act as negative regulators in modulating antiviral innate immune responses upon rhabdovirus infection [15, 16].

Long noncoding RNAs (lncRNAs) are transcribed RNA molecules longer than 200 nucleotides in length that are functionally very diverse and play important roles in various cellular processes, including development, differentiation and metabolism, and disease states [17, 18]. Until now, a series of lncRNAs have been shown to modulate innate immunity in mouse and human. For example, mouse lincRNA-Cox2 plays a central role in control of the Pam3CSK4-induced inflammatory response [19]. Human NEAT1 has been evidenced to interfere with the HIV-1 virion package and posttranscriptional expression [20]. Growing evidences have showed that lncRNAs in mammals can act as competing endogenous RNAs (ceRNAs), known as miRNA sponges or antagomirs, which downregulate miRNAs expression and activity, subsequently modulating the derepression of miRNA targets at the level of post-transcriptional regulation [17, 21]. However, the functions of lncRNA in other vertebrates species, especially in lower vertebrates remain poorly understood.

Viral diseases are the most serious threat to the aquaculture industry. In the past decades, variety of DNA and RNA viruses have been identified as pathogens that result in high mortality in aquaculture species. Rhabdoviruses are a group of enveloped, single stranded, and negative-sense RNA viruses which are one of the most significant viral pathogens in teleost fish and cause severe hemorrhagic septicemia in freshwater and marine fish [22]. Teleost fish is regarded to be an excellent biological model in immunology studies as it is a representative population of lower vertebrates serving as an important link to early vertebrate evolution. In teleost fish, the innate immune response plays a fundamental and central defence during pathogen infection [23]. Similar to mammals, teleost fish possess conserved immune-relevant genes and a series of signaling events in response to invading pathogens. However, compared with mammals, there are indeed a series of experimental techniques and materials limiting in-depth researches on fish immunobiology. For instance, most fish lack the cell lines and it is still difficult to implement gene editing techniques in almost all fish species. Therefore, the detailed signaling pathway of fish antiviral responses and their regulatory mechanisms remain to be detail investigated.

In this study, we identify a ceRNA regulatory network involved in antiviral innate responses in teleost fish, miiuy croaker (*Miichthys miiuy*). We elaborate that fish MAVS contributes to IFN antiviral immunity following the infection of *Siniperca chuatsi rhabdovirus* (SCRV), a typical fish RNA rhabdovirus. miR-122 has been identified to target MAVS and suppress MAVS-mediated antiviral responses, thereby promoting RNA viral replication. Further, our study suggests that a long noncoding RNA, named MAVS antiviral-related lncRNA (MARL), can act as a ceRNA for miR-122 to facilitate MAVS expression, thus modulating MAVS-mediated antiviral responses. To the best of our knowledge, this is the first study to demonstrate ceRNA regulatory networks existing in lower vertebrates, teleost fish. Our data not only provides new insights into understanding lncRNA-miRNA interaction in vertebrates, but reveals the significance of large numbers of non-coding genes in lower vertebrates.

## Results

### Fish MAVS plays an essential role in host antiviral responses

**Fig 1:**
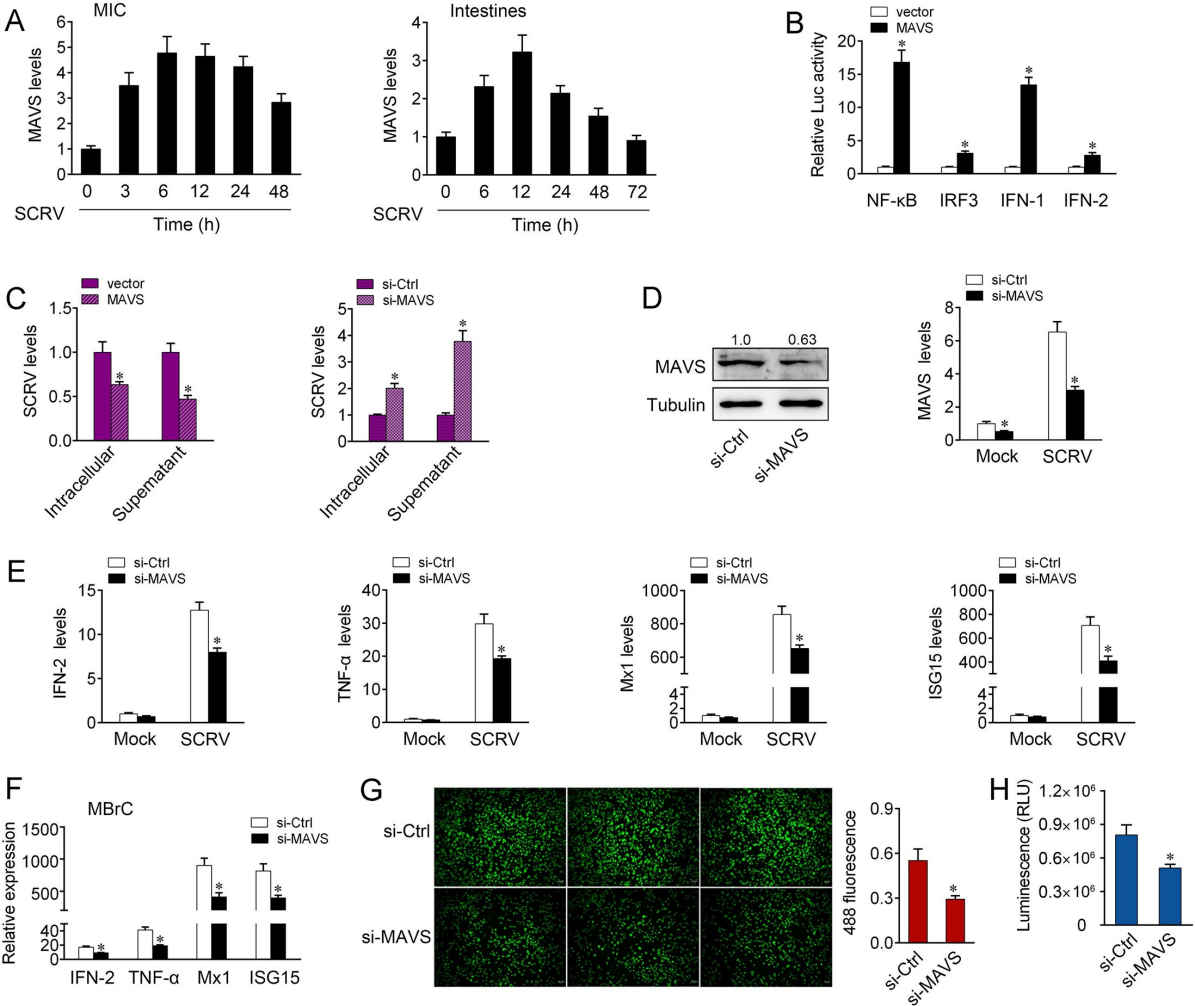
Fish MAVS suppresses antiviral responses upon SCRV infection. (A) SCRV induces an increase of MAVS expression. The expression levels of MAVS in MIC cells and intestine samples were measured by qPCR at indicated time after SCRV infection. (B) MAVS is able to activate NF-κB, IRF3, IFN-1, and IFN-2 signaling. MIC cells were transfected with pRL-TK Renilla luciferase plasmid, luciferase reporter genes, together with MAVS expression plasmid. At 48 h post-transaction, the luciferase activity was measured and normalized to renilla luciferase activity. (C) Fish MAVS suppresses SCRV replication. MIC cells were transfected with pcDNA3.1 vector or MAVS expression plasmid and control siRNA (si-Ctrl) or MAVS-specific siRNA (si-MAVS) for 48 h, then infected with SCRV. The qPCR analysis was conducted for intracellular and supernatant SCRV RNA expression. (D) Knockdown of MAVS attenuates the expression of endogenous MAVS. MIC cells were transfected with si-Ctrl or si-MAVS for 48 h, then the expression levels of MAVS were determined by western blotting and qPCR assays, respectively. (E) Knockdown of MAVS attenuates the expression of antiviral genes. MIC cells were transfected with si-Ctrl or si-MAVS. At 48 h post-transfection, cells were then treated with SCRV for 24 h. The expression of IFN-2, TNF-α, Mx1, and ISG15 were determined by qPCR. (F) MBrC cells were transfected with si-Ctrl or si-MAVS. At 48 h post-transfection, cells were then treated with SCRV for 24 h. The expression of IFN-2, TNF-α, Mx1, and ISG15 were determined by qPCR. (G and H) Effect of MAVS knockdown on cell proliferation and viability after SCRV infection. MIC cells were transfected with either si-MAVS or si-Ctrl. At 48 h post-transfection, the cells were infected with SCRV for 24 h, then cell proliferation assay (G) and cell viability assay (H) were measured. Scale bar, 20 μm; original magnification × 10. All data represented the mean ± SE from three independent triplicated experiments. *, *p* < 0.05.

Viral infection triggers host innate immune responses by activating transcription factors IRF3/IRF7 and NF-κB, which coordinately induce the production of type I IFNs. Mammal MAVS is known as an essential signaling adaptor involved in host antiviral innate immunity in response to RNA virus infection. Recent findings suggest that MAVS homologue genes have been identified in some fish species [24]. However, the signaling pathway involving in fish MAVS-mediated immune response remains poorly understood. To investigate the fish MAVS-mediated signaling pathway in response to RNA virus infection, we firstly examine the expression patterns of fish MAVS upon SCRV. To this end, we treated miiuy croaker intestinal cells (MIC) or intestinal tissues with SCRV to induce immune responses. During SCRV infection, the expression levels of MAVS were significantly increased *in vitro* and *in vivo* (Fig 1A). Given that mammal MAVS activates NF-κB and IRF3/IRF7 to induce IFNs, we therefore tested whether fish MAVS could affect the activation of NF-κB and IRF3. The results from dual-luciferase reporter assays showed that overexpression of MAVS potently activates NF-κB and IRF3 reporter genes, as well as IFN-1 and IFN-2 reporter genes (Fig 1B). Since mammal MAVS overexpression is sufficient to delay the replication virus replication, we determined whether fish MAVS could mediate a similar effect upon RNA virus infection. The results indicated that overexpression of MAVS decreased SCRV replication in the infected cells, while MAVS-specific small interfering RNA (siRNA) to silence the expression of endogenous MAVS exacerbated the viral replication (Fig 1C). These results suggested that similar to mammal MAVS, MAVS in teleost fish could mediate the activation of NF-κB and IRF3 and modulate RNA virus replication.

To confirm whether fish MAVS is required for the induction of type I IFN and inflammatory cytokines upon SCRV infection, we silenced MAVS and examined the expression patterns of indicated genes. Knockdown of MAVS effectively inhibited MAVS expression at both protein and mRNA levels (Fig 1D). Further, as shown in Fig 1E, knockdown of MAVS significantly decreased the expression of IFN-2, as well as antiviral genes and inflammatory cytokines, including TNF-α, Mx1, and ISG15 in MIC under SCRV treatment. In miiuy croaker brain cells (MBrC), MAVS-specific siRNA could also significantly suppress the expression of IFN-2, TNF-α, Mx1, and ISG15 upon SCRV infection (Fig 1F). The result indicated the contribution of MAVS to fish antiviral responses in response to RNA virus infection. Afterwards, to known whether MAVS can affect cell proliferation and viability upon SCRV infection, we used MAVS-specific siRNA for further experiments. As shown in Fig 1G, knockdown of MAVS led to decreasing in cell proliferation. When we explored its effect on cell viability using luminescent cell viability assay, knockdown of MAVS resulted in a decline in cell viability upon SCRV infection (Fig 1H). Collectively, these data verified that similar to mammals, fish MAVS could mediate the activation of NF-κB and IRF3; suppression of fish MAVS expression could block IFNs production, exacerbate viruses replication, and inhibit cell proliferation and viability.

### miR-122 targets MAVS and participates in the regulation of MAVS expression

**Fig 2:**
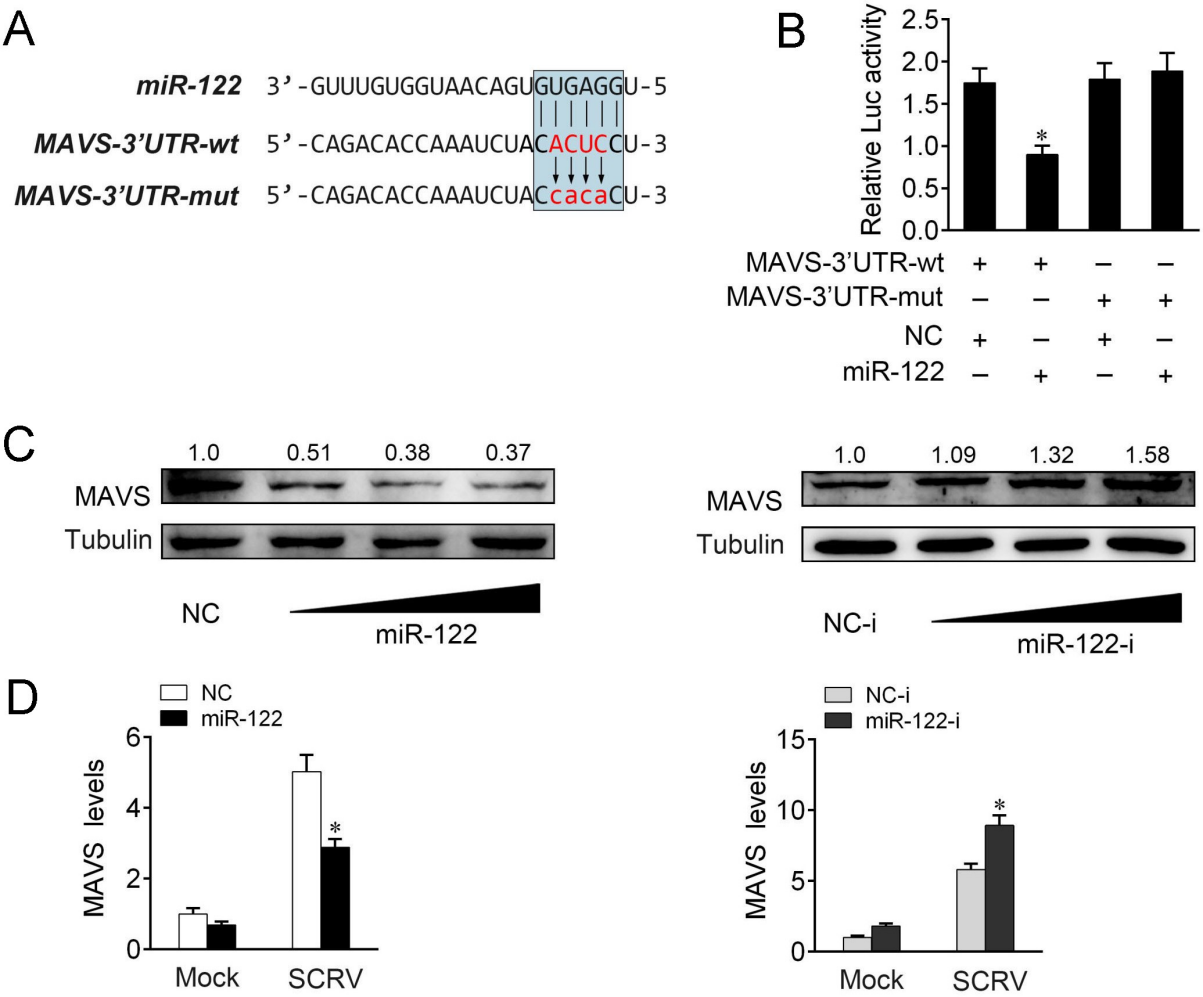
MAVS is a target gene of miR-122. (A) Sequence alignment of miR-122 and its binding sites in the 3’ UTR of MAVS. miR-122 binding sites in wild type of MAVS 3’UTR (MAVS-3’UTR-wt) and a mutated form of 3’UTR (MAVS-3’UTR-mut) were shown. (B) miR-122 target the 3’UTR of MAVS. EPC cells were transfected with control mimics (NC) or miR-122 mimics (miR-122), along with MAVS-3’UTR wt or MAVS-3’UTR-mut for 48 h, then the luciferase activity was determined. The luciferase activity was measured and normalized to renilla luciferase activity. (C) miR-122 suppresses the protein expression of endogenous MAVS. MIC cells were cotransfected with miR-122 or NC and miR-122 inhibitors (miR-122-i) or control inhibitors (NC-i). At 48 h post-transfection, the expression of MAVS were determined by western blotting. (D) miR-122 suppresses the mRNA expression of MAVS. MIC cells were cotransfected with miR-122 or NC and miR-122-i or NC-i. At 48 h post-transfection, cells were then infected with SCRV for 24 h. the expression of MAVS were determined by qPCR. All data represented the mean ± SE from three independent triplicated experiments. *, *p* < 0.05.

miRNAs act as negative regulators of gene expression, and post-transcriptionally regulate the expression of target mRNAs by binding to their 3’UTR. To find out the underlying mechanism by which MAVS is regulated upon viral infection, we tested the possible regulation role of miRNA for MAVS. To obtain direct evidence that miRNA target MAVS gene, we first analyzed the sequence of MAVS 3’UTR and found that miR-122 has a complementary sequence with MAVS 3’UTR (Fig 2A). To verify the binding sites of miR-122 to the 3’UTR of MAVS, mutations were introduced to the 3’UTR of MAVS and a mutated form was constructed (Fig 2A). After cotransfection of luciferase reporter plasmids and miR-122 mimics or control mimics, we observed that miR-122 mimics markedly inhibited the luciferase activity when the wild-type 3’UTR was transfected, whereas the mutated form demonstrated no response to miR-122 mimics (Fig 2B). Furthermore, the gradient experiments of transfection time were conducted with miR-122 mimics, and the results indicated that miR-122 mimics can inhibit the luciferase activity within 12 h to 48 h after transfection, among which it significantly inhibited the luciferase activity at 48 h after transfection (S1A Fig). We also demonstrated that the regulation role of miR-122 mimics on the wild-type 3′UTR presented a dose-dependent manner (S1B Fig). For further confirmation, we cloned the 3’UTR of MAVS into the mVenus-C1 vector, and then explore the function of miR-122 on green fluorescent protein (GFP) expression. As shown in S1C Fig, miR-122 mimics downregulate GFP gene expression, whereas no change on fluorescence intensity was observed in cells transfected with the mutated form of MAVS 3’UTR. To extend the findings, the western blotting assay has been conducted to examine the expression level of GFP protein (S1D Fig). Additionally, we applied RNA immunoprecipitation (RIP) experiments to test the potential direct binding between MAVS 3’UTR and miR-122. The results from qPCR analysis showed that the MAVS-3’UTR RIP was significantly enriched for miR-122 compared to the mutated type of MAVS 3’UTR plasmids or empty vector (MS2) (S1E and S1F Fig).

To test whether miR-122 participates in the regulation of MAVS expression, we first sought to cotransfect of miR-122, together with MAVS expression plasmid into fish epithelioma papulosum cyprini cells (EPC). To construct MAVS expression plasmid, the full-length CDS and 3’UTR of miiuy croaker MAVS regions were amplified and cloned into pcDNA3.1 vector with a flag tag. As indicated in S1G Fig, miR-122 significantly decreased MAVS expression levels in a dose-dependent manner. To measure miR-122 function in the regulation of endogenous MAVS, we transfected with miR-122 mimics or inhibitors into MIC cells to test the levels of endogenous MAVS. As shown in Fig 2C, transfection of miR-122 mimics obviously suppressed the protein levels of MAVS, whereas miR-122 inhibitors markedly enhanced its expression levels in a dose-dependent manner. Additionally, we further investigated whether miR-122 affect the stability of MAVS mRNA. Transfection of miR-122 mimics led to a reduction of MAVS expression upon SCRV treatment, while tansfection of miR-122 inhibitors induced an increase in MAVS expression (Fig 2D). Collectively, these results suggested that MAVS is a direct target of miR-122, and miR-122 regulates MAVS expression at post-transcriptional levels.

### miR-122 suppresses antiviral responses

**Fig 3:**
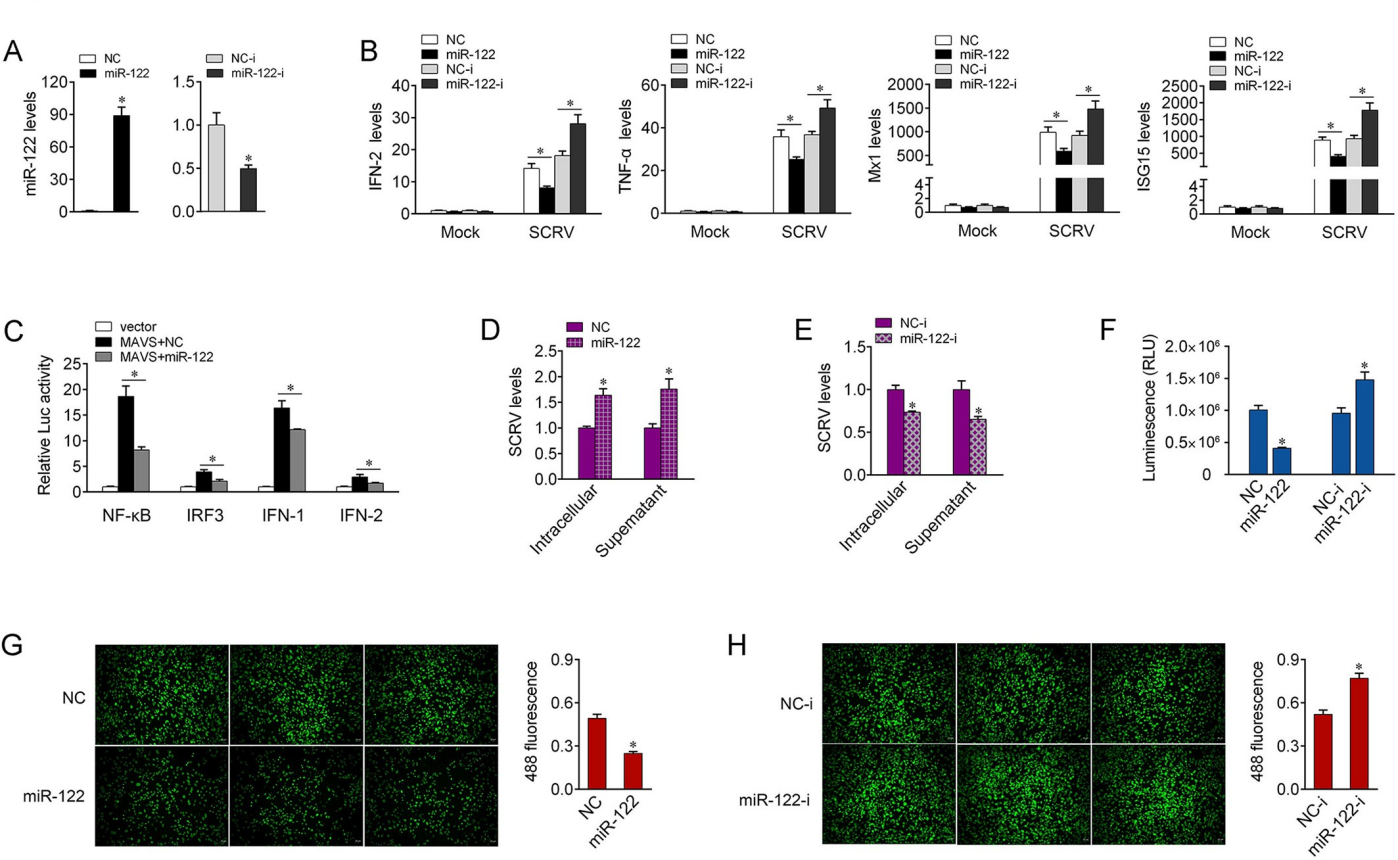
miR-122 inhibits the antiviral responses upon SCRV infection. (A) The effect of miR-122 mimics and inhibitors on endogenous miR-122 expression. MIC cells were transfected with NC or miR-122 (left panel), and NC-i and miR-122-i (right panel) for 48 h, then miR-122 expression was determined by qPCR. (B) Overexpression of miR-122 attenuates the expression of INF-2 and antiviral genes. MIC cells were transfected with NC, miR-122, NC-i or miR-122-i. At 48 h post-transfection, the cells were treated with SCRV for 24 h. The expression levels of IFN-2, TNF-α, Mx1, and ISG15 were analyzed by qPCR. (C) miR-122 could suppress NF-κB, IRF3, IFN-1, and IFN-2 signaling. MIC cells were transfected with NC or miR-122, together with MAVS expression plasmid, pRL-TK Renilla luciferase plasmid, and luciferase reporter genes. At 48 h post-transaction, the luciferase activity was measured and normalized to renilla luciferase activity. (D and E) miR-122 enhances SCRV replication. MIC cells were transfected with NC or miR-122 (D) and NC-i or miR-122-i (E) for 48 h, then infected with SCRV. The qPCR analysis was conducted for intracellular and supernatant SCRV RNA expression. (F) Effect of miR-122 on cell viability after SCRV infection. MIC cells were transfected with NC, miR-122, NC-i or miR-122-i for 48 h, and then treated with SCRV. Cell viability assay were measured. (G and H) Effect of miR-122 on cell proliferation after SCRV infection. MIC cells were transfected with NC or miR-122 (G) and NC-i or miR-122-i (H) for 48 h, then treated with SCRV for 24 h. Cell proliferation assay were measured. Scale bar, 20 μm; original magnification × 10. All data represented the mean ± SE from three independent triplicated experiments. *, *p* < 0.05.

To explore the function role of miR-122 in host antiviral responses, the expression profiles of miR-122 under SCRV treatment were firstly performed *in vitro* and *in vivo*. The result showed that SCRV-induced miR-122 upregulation in MIC cells was confirmed by qPCR (S2A Fig). We also detected the expression of miR-122 in intestinal tissues, and the expression of miR-122 was significantly increased upon SCRV treatment (S2B Fig). We thus investigated the regulation role of miR-122 in the production of type I IFNs and inflammatory cytokines. To this end, we first measured the effects of synthetic miR-122 mimics and miR-122 inhibitors on the expression of miR-122. As expected, miR-122 mimics enhanced miR-122 expression sharply, whereas miR-122 inhibitors decreased miR-122 expression (Fig 3A). Then, the effect of miR-122 and SCRV on the expression patterns of the indicated genes were evaluated. The results showed that certain inflammatory cytokines and antiviral genes, including IFN-2, TNF-α, Mx1, and ISG15 were significantly decreased by the introduction of miR-122 mimics. On the contrary, the inhibition of endogenous miR-122 significantly elevated these gene expression compared with transfection of control inhibitors (Fig 3B). Given that miR-122 targets MAVS and regulates its expression, we thus wanted to test whether miR-122 affects MAVS-mediated activation of NF-κB and IRF3. The results from dual-luciferase reporter assays showed that after cotransfection of MAVS expression plasmid, miR-122 mimics obviously suppressed the activity of NF-κB, IRF3, IFN-1, and IFN-2 luciferase reporters activated by MAVS overexpression compared with control mimics (Fig 3C). To investigate the biological significance of miR-122 in SCRV-induced host cells, we examined the effect of miR-122 on SCRV replication. Overexpression of miR-122 increased, whereas inhibition of miR-122 decreased SCRV RNA expression in the intracellular and supernatant from the infected cells (Fig 3D and 3E), which indicated that host miR-122 may help viruses evade host antiviral responses. Next, we attempted to investigate whether miR-122 is related to regulate cell proliferation and viability after SCRV infection. As shown in Fig 3F–3H, overexpression of miR-122 resulted in a reduction in cell viability and proliferation upon SCRV infection, whereas the inhibition of miR-122 expression led to an efficiently increase in cell viability and proliferation. Collectively, these data demonstrated that inducible miR-122 is able to inhibit antiviral responses and enhance SCRV replication, which may help viruses evade host immune responses.

### LncRNA MARL is able to regulate miR-122 expression and activity

**Fig 4:**
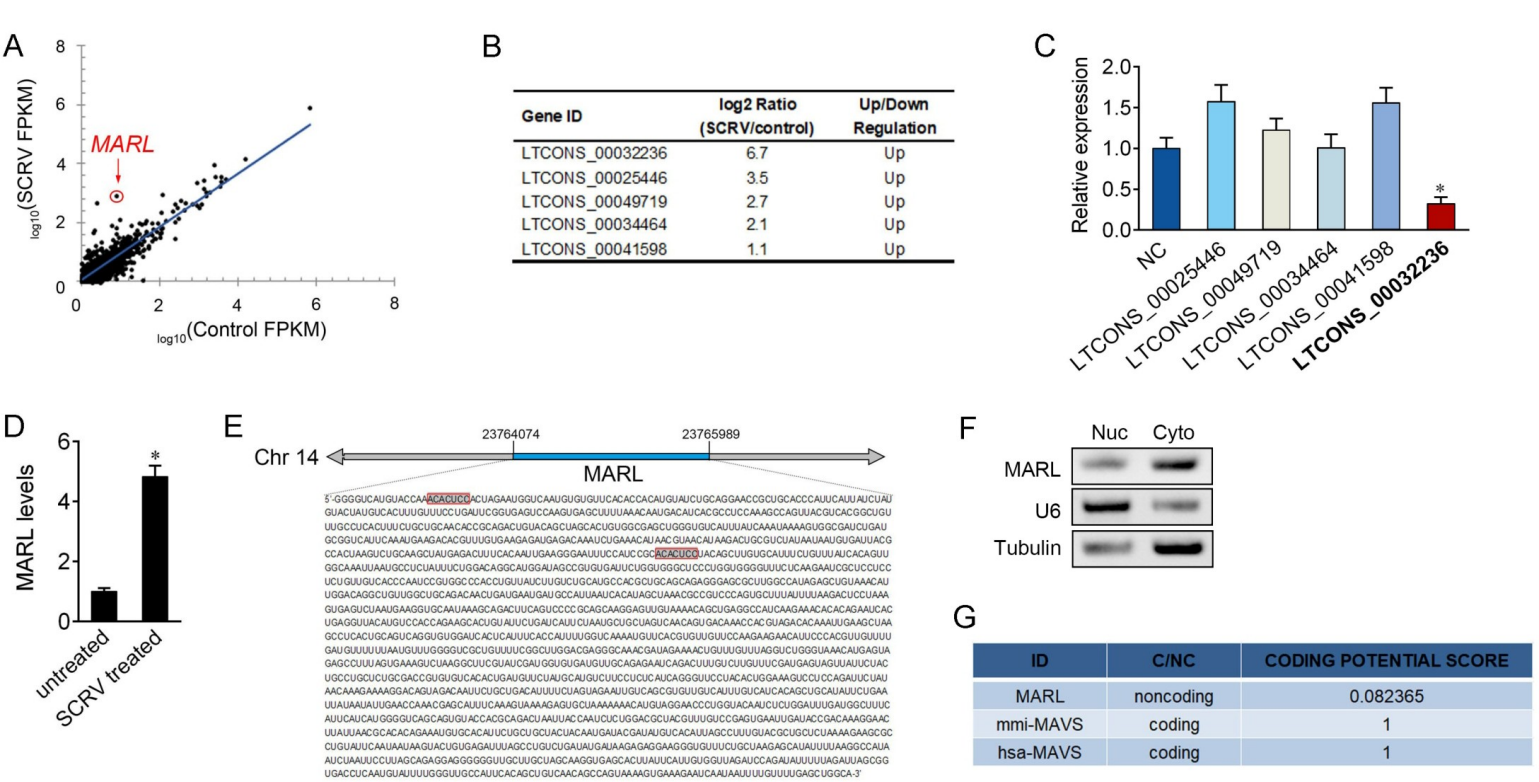
Identification and characterization of MARL. (A) Scatter plot of differentially expressed lncRNAs from untreated (control) and SCRV treated spleen tissues. For the scatter plot, X-axis and Y-axis present log10 value of FPKM of untreated and treated samples, respectively. MARL was one of the top lncRNAs that was found to be significantly upregulated upon SCRV infection. (B) Differentially expressed lncRNAs within the miR-122 miRNA response elements. LncRNAs with log2 (SCRV/Control) ≥ 1 and *p*-value < 0.05 were defined as the significantly differential ones. (C) Relative expression of five lncRNAs in MIC cells treated with miR-122 mimics were measured by qPCR. (D) Miiuy croaker was untreated or treated with SCRV. After 48 h treatment, the expression levels of MARL in spleen samples were measured by qPCR. (E) Schematic of the MARL locus. MARL locates on miiuy croaker chromosome 14, and miR-122 binding site was shown in boxes. (F) MARL is mainly localized in the cytoplasm of MIC cells. RNA isolated from nuclear (Nuc) and cytoplasm (Cyto) was used to analyze the expression of MARL by semi-quntitative PCR; *n* = 3. (G) MARL were predicted to be non-coding RNAs. The RNA sequences of MARL was put into the CPC software, which was predicted to be non-coding RNAs. mmi-MAVS, *Miichthys miiuy* MAVS gene; hsa-MAVS, *Homo sapiens* MAVS gene.

To identify lncRNAs that are potentially involved in the regulation of SCRV infection, we treated miiuy croaker with SCRV for 48 h, then used RNA-seq analysis to compare lncRNA expression levels between SCRV treated and untreated spleen samples. From the deep-sequencing data, we identified 897 lncRNAs that were differentially expressed (Fig 4A). Recent studies have reported that lncRNAs can act as endogenous sponge RNAs to interact with miRNAs and influence miRNA expression [17, 21]. Given that miR-122 participates in the regulation of antiviral responses, we would like to examine whether miR-122 participates alone or acts as a member of the intricate network. Among these 897 differentially expressed lncRNAs, we identified five differentially expressed lncRNAs within the miR-122 miRNA response elements (MREs) (Fig 4B). To detect the correlation between the expression of miRNA and these candidate lncRNA, we overexpressed miR-122 and measured the expression of candidate lncRNAs through qPCR analysis. Among the five lncRNAs, LTCONS_00032236 (termed MARL) presented significantly downregulation in response to miR-122 overexpression (Fig 4C).

To confirm the RNA-seq result that MARL was significantly enriched in the treated samples, we treated miiuy croaker with SCRV and measured the expression of MARL. As shown in Fig 4D, SCRV induced a robust increase of MARL in spleen samples. To characterize the complete sequence of MARL, single-molecule full-length transcript sequencing (Iso-Seq) were used and demonstrated that the length of MARL was 1923 base pairs (bp) and locates on miiuy croaker chromosome 14 (Fig 4E). To detect the subcellular location of MARL, we conducted the RT-PCR to indicate the nuclear and cytoplasmic fractions of MARL, and found that MARL mainly expressed in the cytoplasm (Fig 4F). Consistent with MARL being a noncoding RNA, the CPC (coding potential calculator) computational algorithm [25] predicts that MARL has a very low coding potential (Fig 4G).

**Fig 5:**
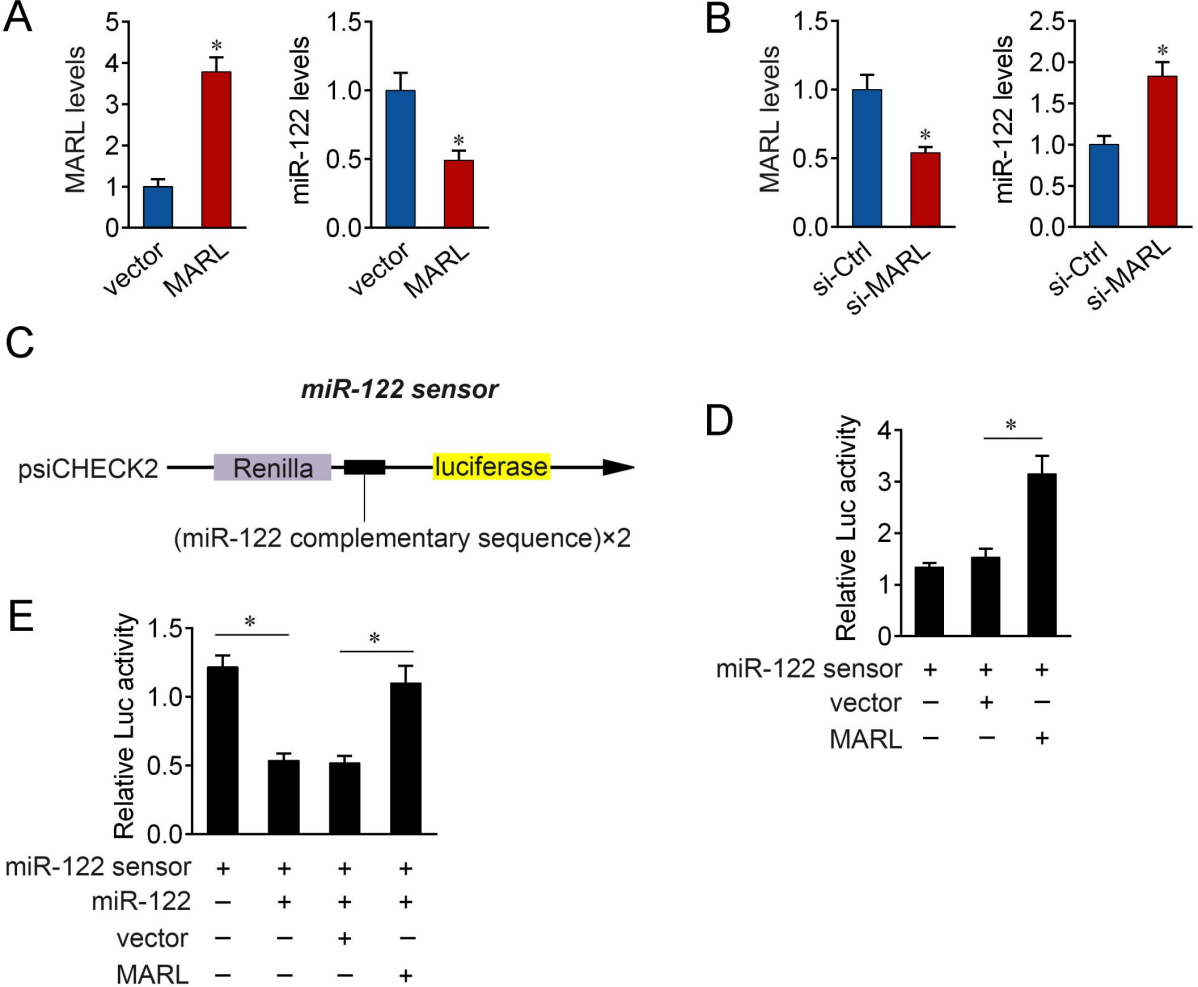
MARL regulates miR-122 expression and activity. (A) Overexpression of MARL reduces the expression of miR-122. Transfection of pcDNA3.1 vector or MARL expression plasmid into MIC cells for 48 h. The expression of MARL and miR-122 were measured by qPCR, respectively. (B) Knockdown of MARL upregulated miR-122 expression. MIC cells were transfected with MARL-specific siRNAs (si-MARL) or si-Ctrl for 48 h. The expression of MARL and miR-122 were measured by qPCR, respectively. (C) miR-122 sensor construct. The miR-122 sensor was constructed by inserting two copies of perfectly matched miR-122 fragments into psiCHECK-2 vector. (D) MARL overexpression induced the luciferase activity. MIC cells were transfected with pcDNA3.1 vector or MARL expression plasmid, together with miR-122 sensor. At 48 h post-transfection, the luciferase activity was analyzed. (E) MARL reduces miR-122 activity. MIC cells were transfected with miR-122, pcDNA3.1 vector, or MARL expression plasmid, together with miR-122 sensor for 48 h. The luciferase activity was analyzed and normalized to renilla luciferase activity. All data represented the mean ± SE from three independent triplicated experiments. *, *p* < 0.05.

To known whether MARL is involved in the regulation of miR-122 expression, we separately overexpressed or knocked down of MARL. The results showed that miR-122 expression is separately decreased or elevated in the cells on overexpression or knockdown of MARL (Fig 5A and 5B). To known whether MARL can affect miR-122 activity, we constructed a miR-122 sensor. The miR-122 sensor was constructed by inserting two copies of perfectly matched miR-122 fragments into psiCHECK-2 vector, and a reduced luciferase activity of sensor indicated the induction of miR-122 activity (Fig 5C). Our results showed that overexpression of MARL induced the luciferase activity under the transfection of miR-122 sensor. (Fig 5D). Furthermore, we found that the decreased luciferase activity induced by miR-122 was recovered when cotransfected with MARL expression plasmid, suggesting that MARL specifically sponged miR-122, thereby preventing it from inhibiting luciferase activity (Fig 5E). Taken together, these data suggest that MARL is able to regulate miR-122 expression and activity.

### MARL is able to directly bind to miR-122

**Fig 6:**
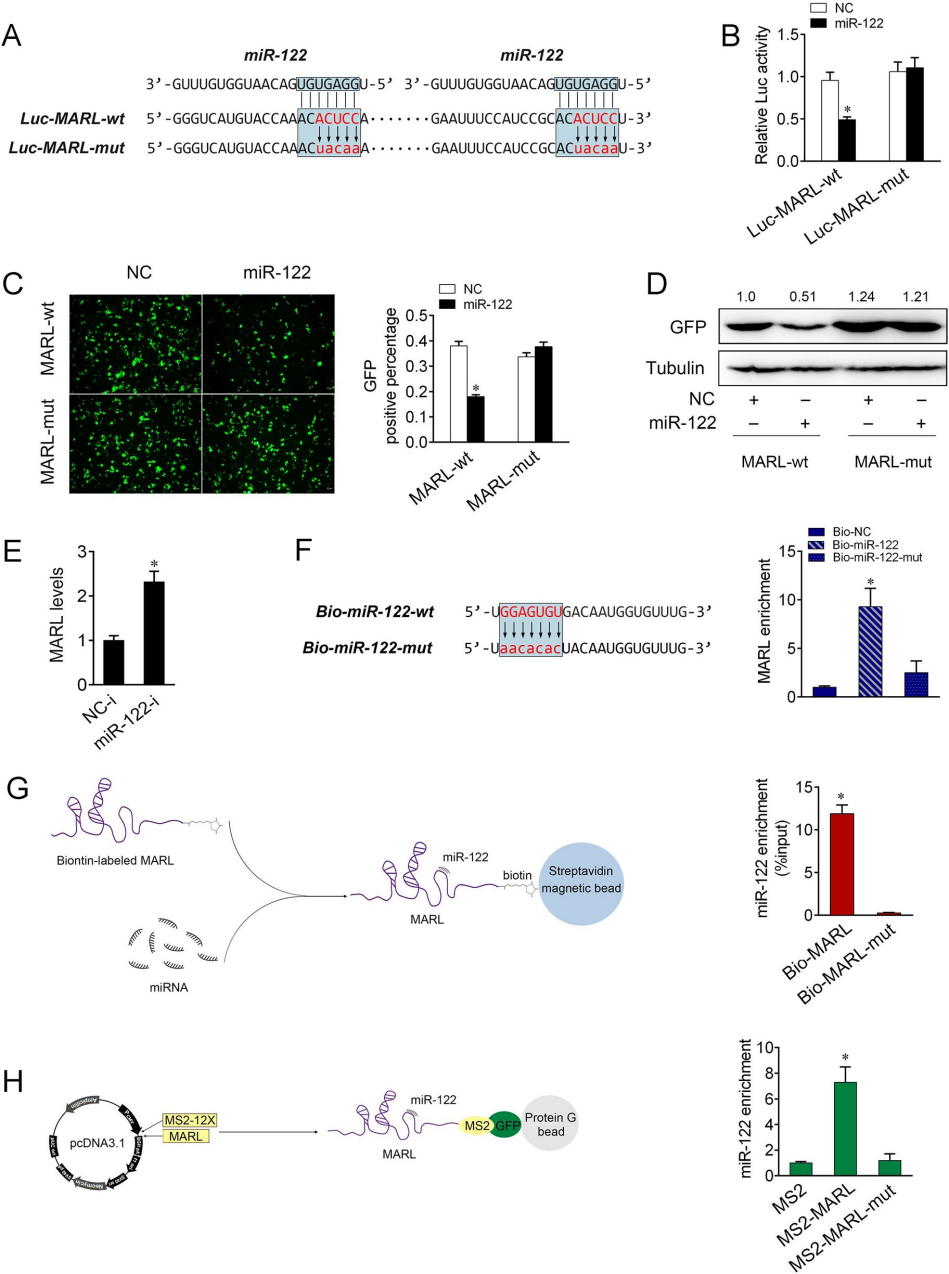
miR-122 interacts with MARL. (A) MARL sequence contains two sites complementary to miR-122. miR-122 binding sites in MARL wild-type form (Luc-MARL-wt) and the mutated form (Luc-MARL-mut) were shown. (B) EPC cells were transfected with NC or miR-122, together with Luc-MARL-wt or Luc-MARL-mut. At 48 h post-transaction, the luciferase activity was analyzed and normalized to renilla luciferase activity. (C and D) MARL could downregulate GFP expression. EPC cells were cotransfected with the wild type of mVenus-MARL or the mutated type, together with NC or miR-122. At 48 h post-transfection, the fluorescence intensity (C) and the GFP expression (D) were evaluated by enzyme-labeled instrument and western blotting, respectively. Scale bar, 20 μm; original magnification × 10. (E) MIC cells were transfected with NC-i or miR-122-i for 48 h. The expression of MARL were measured by qPCR. (F) The wild and mutated forms of biotinylated miR-122 sequence were shown (left panel). MIC cells were transfected with the biotinylated wild type of miR-122 (Bio-miR-122-wt) or the biotinylated mutated type of miR-122 (Bio-miR-122-mut) for 48 h. Cells were harvested for biotin-based pulldown assay. MARL expression were analyzed by qPCR (right panel). (G) The schematic diagram of the RNA pull down method to identify the binding between MARL and miR-122 (left panel). MIC lysates were incubated with biotin-labeled MARL and MARL-mut. miRNA real-time PCR was performed after pull down process (right panel). (H) The schematic diagram of RIP method (left panel). The qPCR results of the MS2-RIP method used to identify the binding between MARL and miR-122 in MIC cells. miRNA real-time qPCR was performed after RNA immunoprecipitation process (right panel). All data represented the mean ± SE from three independent triplicated experiments. *, *p* < 0.05.

To understand the mechanism by which MARL regulates miR-122 expression, we examined whether MARL can interact with miR-122. To this end, we analyzed the sequences of MARL and noticed that MARL contains two binding site of miR-122 (Fig 6A). We constructed MARL luciferase plasmid and the mutated form with miR-122 binding sites mutated (Fig 6A). Luciferase assays revealed that miR-122 could inhibit the luciferase activity of the wild form of MARL luciferase plasmid, but it had no effect on its mutated form (Fig 6B). Subsequently, the gradient experiments with transfection time or miR-122 dose were conducted. As shown in S3A and S3B Fig, the results indicated that miR-122 mimics produced a significantly inhibition on the luciferase activity at 48 h after transfection, which appeared to be a dose-dependent manner. For further validation, we inserted the wild or mutated form of MARL into mVenus-C1 vector, and examined whether cotransfecting with miR-122 could suppress the expression of GFP. As shown in Fig 6C and 6D, the results revealed that miR-122 could significantly inhibit GFP expression, indicating the interact between miR-122 and MARL. Furthermore, we performed qPCR analysis to detect MARL expression in cells after cotransfection of miR-122 inhibitors (Fig 6E). Collectively, these data revealed that MARL may interact with miR-122 by putative binding sites.

Further, we performed biotin-avidin pulldown experiments to examine whether miR-122 could pull down MARL. MIC cells were transfected with biotinylated miR-122 or a mutated form, then harvested for pulldown assay (Fig 6F, left panel). MARL was pulled down and analyzed by qPCR, but the introduction of mutations that disrupt base pairing between MARL and miR-122 led to the inability of miR-122 to pull down MARL, indicating that the recognition of miR-122 to MARL is in a sequence-specific manner (Fig 6F and S3C Fig). We also used inverse pulldown assay to test whether MARL could pull down miR-122. MARL and the mutated type of MARL were transcribed *in vitro*, which were labeled with biotin and then incubated with cell lysates before isolation with streptavidin agarose beads. The results from qPCR analysis revealed that miR-122 could be pulled down by biotin-labeled MARL, but not the mutated type (Fig 6G and S3D Fig). Additionally, we applied RIP experiments to test the potential direct binding between MARL and miR-122. To construct plasmids that could produce lncRNAs identified by the MS2 protein, we cloned an MS2-12X fragment into pcDNA3.1, pcDNA3.1-MARL, and the mutated type of MARL plasmids (pcDNA3.1-MARL-mut). We also constructed a GFP and MS2 gene fusion expression plasmid to produce a GFP-MS2 fusion protein that could bind with the MS2-12X fragment and be identified using an anti-GFP antibody. Hence, miRNAs that interact with MARL could be pulled down by the GFP-MS2-lncRNA compounds. The results from qPCR analysis showed that the pcDNA3.1-MARL RIP was significantly enriched for miR-122 compared to pcDNA3.1-MARL-mut or empty vector (MS2) (Fig 6H and S3E Fig).

### MARL participates in regulating the antiviral innate immunity

**Fig 7:**
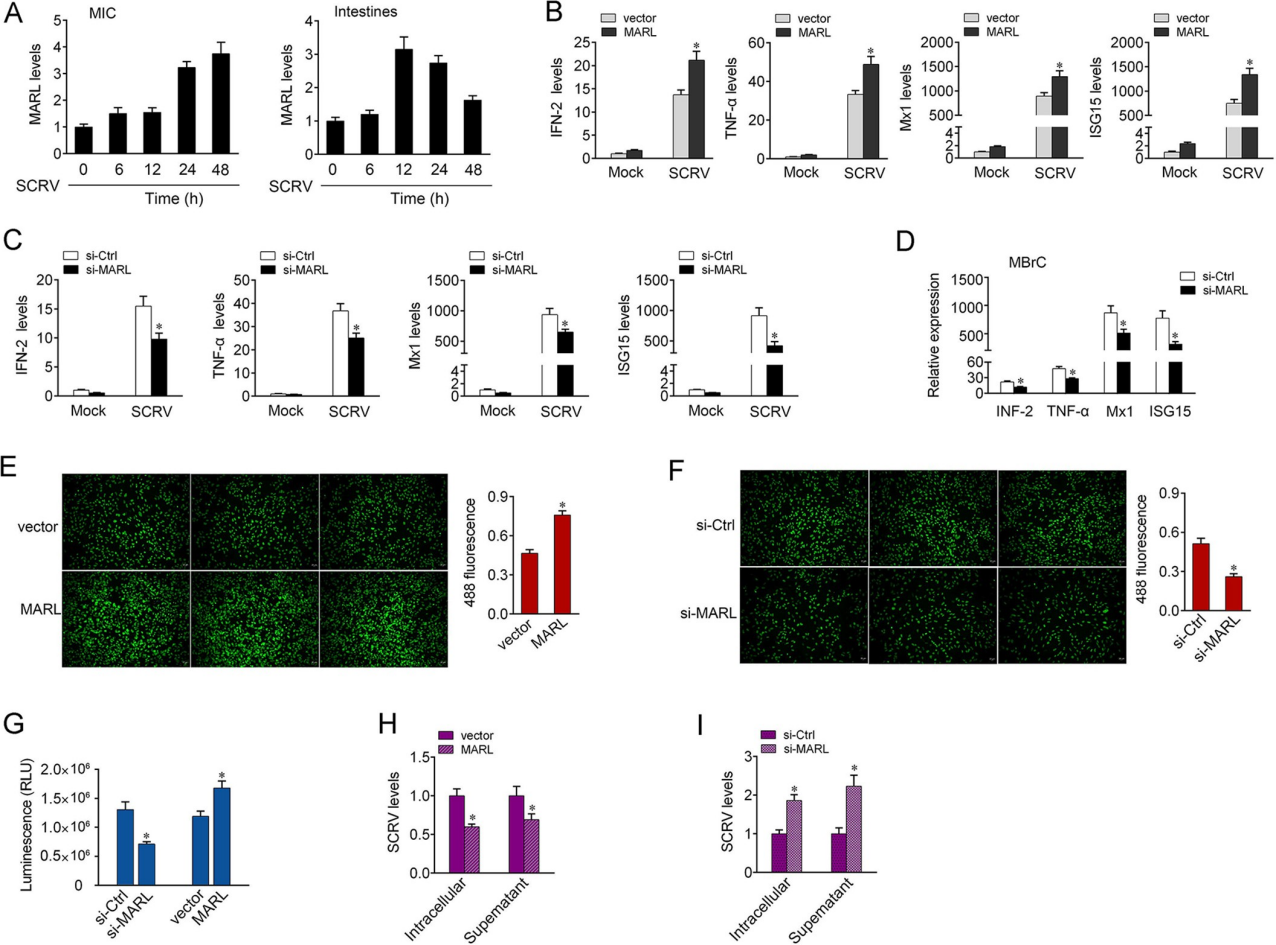
MARL enhances host antiviral responses upon SCRV infection. (A) SCRV induces an increase of MARL expression. The expression levels of MARL in MIC cells and intestine samples were measured by qPCR at indicated time after SCRV infection. (B) MIC cells were transfected with pcDNA3.1 vector or MARL expression plasmid for 48 h. The cells were treated with SCRV for 24 h. The expression of IFN-2, TNF-α, Mx1, and ISG15 were analyzed by qPCR. (C) MIC cells were transfected with si-Ctrl or si-MARL. At 48 h post-transfection, MIC cells were treated with SCRV for 24 h. The expression of IFN-2, TNF-α, Mx1, and ISG15 were analyzed by qPCR. (D) MBrC cells were transfected with si-Ctrl or si-MARL. At 48 h post-transfection, cells were then treated with SCRV for 24 h. The expression of IFN-2, TNF-α, Mx1, and ISG15 were determined by qPCR. (E) Effect of MARL on cell proliferation after SCRV infection. MIC cells were transfected with pcDNA3.1 vector or MARL expression plasmid. At 48 h post-transfection, the cells were infected with SCRV, then cell proliferation assays were measured. Scale bar, 20 μm; original magnification × 10. (F) Effect of si-MARL on cell proliferation after SCRV infection. MIC cells were transfected with si-Ctrl or si-MARL. At 48 h post-transfection, the cells were infected with SCRV, then cell proliferation assays were measured. Scale bar, 20 μm; original magnification × 10. (G) Effect of MARL on cell viability after SCRV infection. MIC cells were transfected with si-Ctrl, si-MARL, pcDNA3.1 vector or MARL for 48 h, then treated with SCRV for 24 h. Cell viability assay were measured. (H and I) MARL suppresses SCRV replication. MIC cells were transfected with pcDNA3.1 vector or MARL expression plasmid (H) and si-Ctrl or si-MARL (I) for 48 h, then infected with SCRV. The qPCR analysis was conducted for intracellular and supernatant SCRV RNA expression. All data represented the mean ± SE from three independent triplicated experiments. *, *p* < 0.05.

Because MARL can interact with miR-122, we thus wanted to measure whether MARL is able to regulate antiviral responses. We first examined the expression patterns of MARL upon SCRV infection, and the results showed that SCRV treatment led to a time-dependent elevation of MARL expression levels in MIC cells, as well as in intestinal tissues (Fig 7A). Since IFN-stimulated genes (ISGs) are important antiviral effectors, we then focused on studying the function of MARL in regulating the expression of ISGs genes. As shown in Fig 7B and 7C, overexpression of MARL induced a rising of certain gene expression, including IFN-2, TNF-α, Mx1, and ISG15 in MIC cells upon SCRV treatment, whereas knockdown of MARL reduced the expression of the indicated genes. In MBrC cells, knockdown of MARL could also significantly inhibit the expression of IFN-2, TNF-α, Mx1, and ISG15 upon SCRV infection (Fig 7D). To further explore the role of MARL in antiviral innate immunity, we examined the cell proliferation. As shown in Fig 7E and 7F, overexpression of MARL produced an increase effect on cell proliferation, but knockdown of MARL showed the opposite regulatory effect on cells after SCRV infection. Simultaneously, when we explored its effect on cell viability, we found that knockdown of MARL inhibited cell viability, while overexpression of MARL led to an increase effect on cell viability (Fig 7G). Additionally, to investigate the biological significance of MARL in SCRV-induced host cells, we examined the effect of MARL on SCRV replication. By measuring the SCRV RNA levels, we found that overexpression of MARL suppressed SCRV replication, whereas knockdown of MARL facilitated SCRV replication (Fig 7H and 7I). Collectively, these data suggest that MARL plays as a positive modulator involved in the regulation of antiviral immunity.

### MARL acts as a sponge for miR-122 to enhance MAVS expression

**Fig 8:**
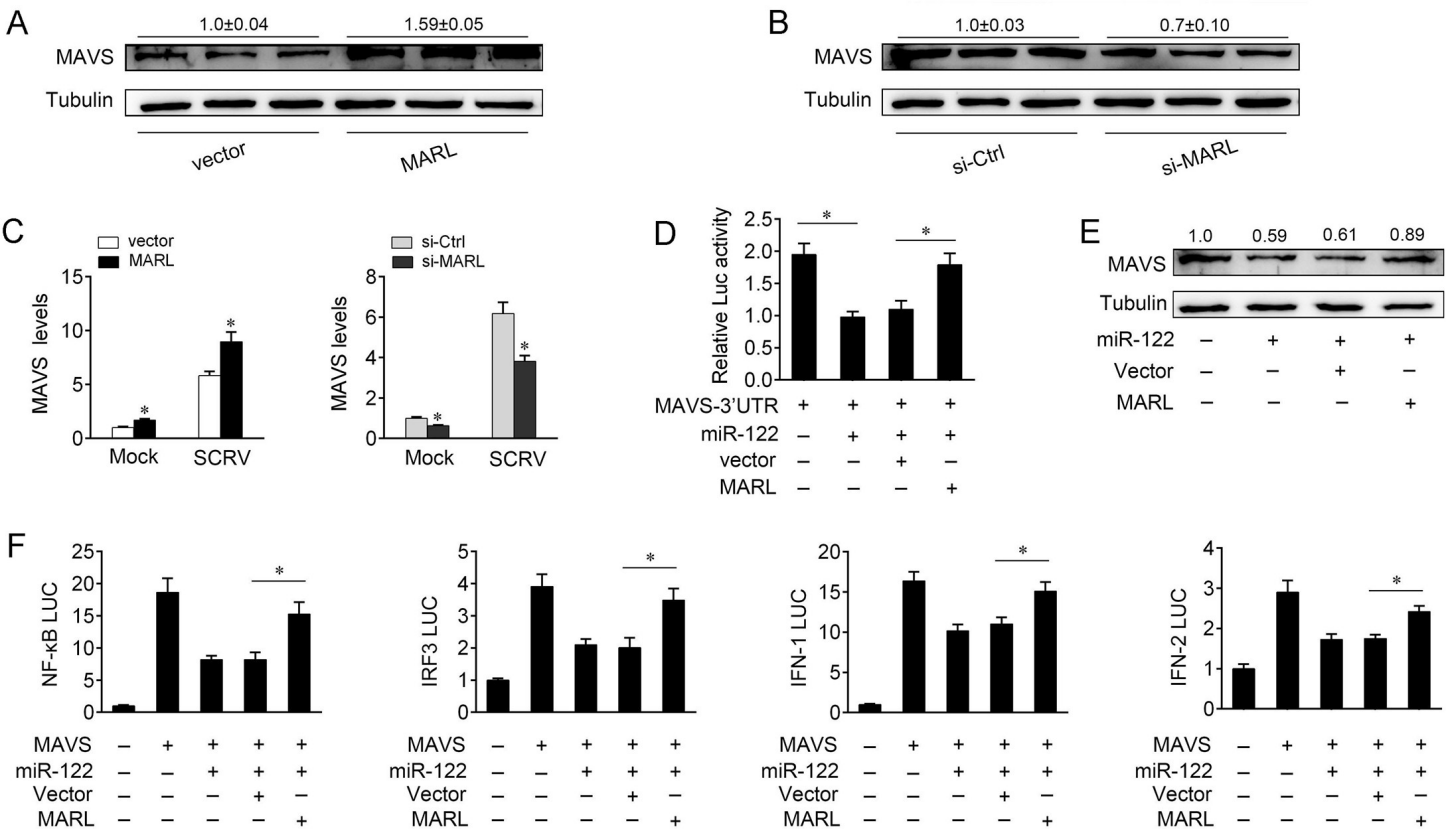
MARL acts as a sponge for miR-122 to facilitate MAVS expression. (A and B) MARL regulate the expression of MAVS. MIC cells were transfected with pcDNA3.1 vector or MARL expression plasmid (A) and si-Ctrl or si-MARL (B) for 48 h, then the MAVS expression was analyzed by western blotting. (C) MARL regulate the mRNA expression of MAVS upon SCRV expression. MIC cells were transfected with pcDNA3.1 vector or MARL expression plasmid (left panel) and si-Ctrl or si-MARL (right panel) for 48 h, then treated with SCRV for 24 h. The MAVS expression was analyzed by qPCR assays. (D) MARL counteracts the inhibitory effect of miR-122 on MAVS 3’UTR. MIC cells were transfected with NC, miR-122, pcDNA3.1 vector or MARL expression plasmid, together with MAVS 3’UTR luciferase reporter genes for 48 h. Luciferase activity was analyzed and normalized to renilla luciferase activity. (E) MARL counteracts the inhibitory effect of miR-122 on MAVS expression. MIC cells were tranfected with miR-122, NC, pcDNA3.1 vector or MARL expression plasmid, together with MAVS expression plasmid for 48 h. MAVS expression were analyzed by western blotting. (F) MARL counteracts the negative effect of miR-122 on the luciferase activities of NF-κB, IRF3, IFN-1, and IFN-2 reporter genes. MIC cells were cotransfected with pRL-TK Renilla luciferase plasmid, luciferase reporter genes, pcDNA3.1 vector or MARL expression plasmid, together with MAVS expression plasmid and miR-122 mimics for 48 h. The luciferase activity was measured and normalized to renilla luciferase activity. All data represented the mean ± SE from three independent triplicated experiments. *, *p* < 0.05.

Given that MARL interacts with miR-122 and miR-122 targets MAVS and regulates its expression, we then tested whether MARL was able to regulate MAVS. Overexpression of MARL resulted in the upregulation of MAVS expression, whereas knockdown of MARL reduced MAVS expression (Fig 8A and 8B). Further, the function of MARL on the mRNA expression of MAVS have been measured in cells upon SCRV treatment. As shown in Fig 8C, overexpression of MARL led to an increasing in mRNA levels of MAVS in cells treated with SCRV. In contrast, knockdown of MARL reduced MAVS expression upon SCRV infection. We then tested whether MARL regulates MAVS expression through miR-122. To this end, we contransfected with MAVS 3’UTR, together with miR-122 or MARL expression plasmid. Luciferase assays showed that MARL could counteract the inhibitory effect of miR-122 on MAVS 3’UTR (Fig 8D and S4A Fig). Strikingly, MARL could also counteract that effect of miR-122 on MAVS protein expression (Fig 8E and S4B Fig). These results demonstrate that MARL regulates MAVS expression through miR-122. Given that MAVS, as well as miR-122 participates in the activation of NF-κB, IRF3, IFN-1, and IFN-2 reporter genes (Figs 1B and 3C), we further examined the function role of MARL in regulating these reporter genes. Luciferase assays showed that MARL could counteract the negative effect of miR-122 on the luciferase activities of NF-κB, IRF3, IFN-1, and IFN-2 reporter genes (Fig 8F and S4C Fig). Taken together, the data suggest that MARL appears to serve as a sponge for miR-122 to promote MAVS expression, thereby regulating antiviral responses.

### The ceRNA network of regulating MAVS is widely found in teleost fish

**Fig 9:**
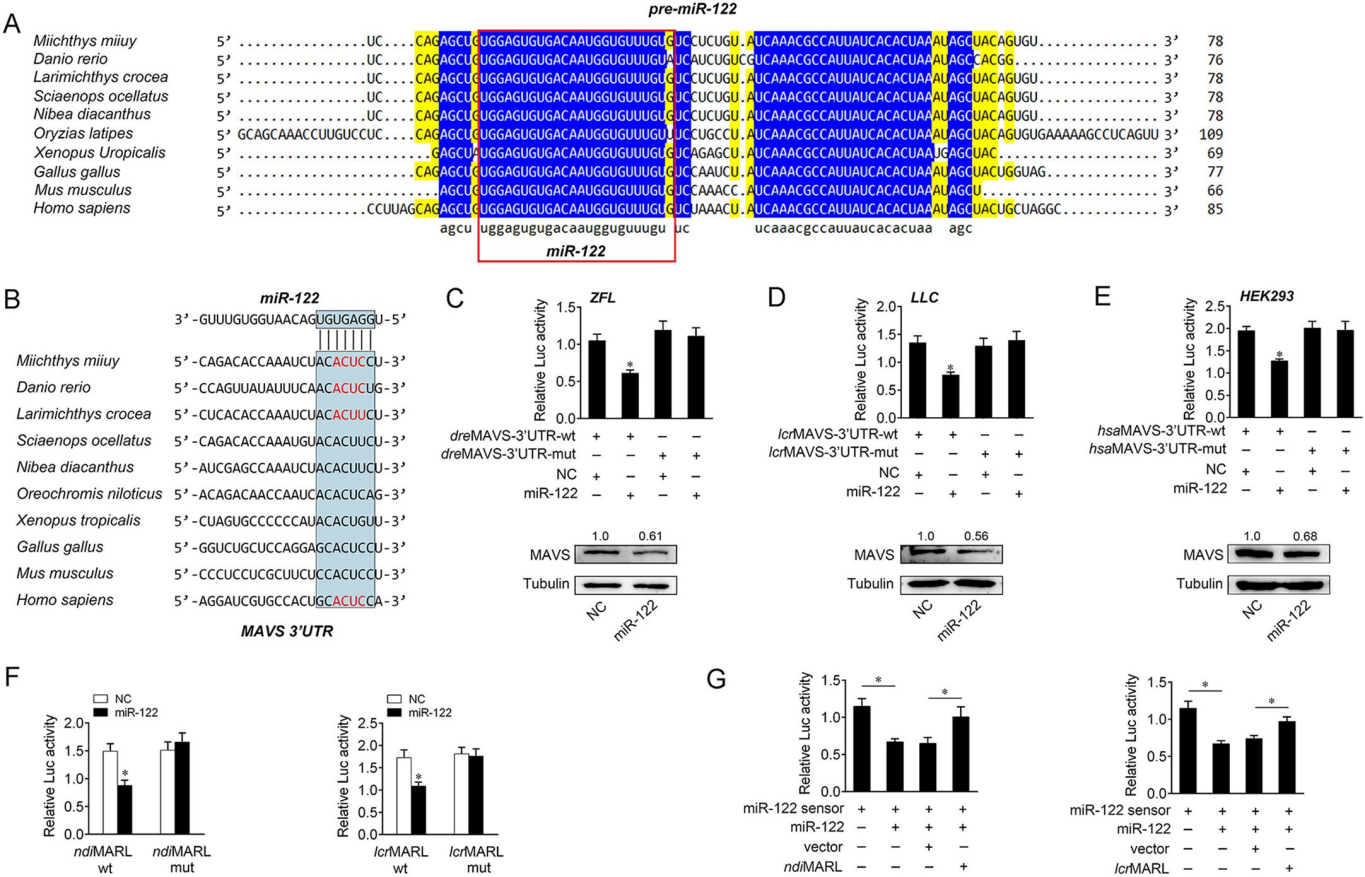
The ceRNA network of regulating MAVS is widely found in other species. (A) Sequence alignment of pre–miR-122 from various vertebrate species. Mature miR-122 sequences are shown in boxes. (B) Putative miR-122-binding site of MAVS 3’UTR among different vertebrate species. (C) miR-122 target *D*. *rerio* MAVS 3’UTR and regulates its expression. ZFL cells were transfected with NC or miR-122, along with the wild-type of *D*. *rerio* MAVS 3’UTR (*dre*MAVS-3’UTR-wt) or the mutant-type (*dre*MAVS-3’UTR-mut). The luciferase activity was measured and normalized to renilla luciferase activity (upper panel). ZFL cells were transfected with NC or miR-122 for 48 h. MAVS expression were analyzed by western blotting (lower panel). (D) miR-122 target *L*. *crocea* MAVS 3’UTR and regulates its expression. LLC cells were transfected with NC or miR-122, along with the wild-type of *L*.*crocea* MAVS-3’UTR (*lcr*MAVS-3’UTR-wt) or the mutant-type (*lcr*MAVS-3’UTR-mut). The luciferase activity was measured and normalized to renilla luciferase activity (upper panel). LLC cells were transfected with NC or miR-122 for 48 h. MAVS expression were analyzed by western blotting (lower panel). (E) miR-122 target *H*. *Sapiens* MAVS 3’UTR and regulates its expression. HEK293 cells were transfected with NC or miR-122, along with the wild-type of *H*. *sapiens* MAVS-3’UTR (*hsa*MAVS-3’UTR-wt) or the mutant-type (*hsa*MAVS-3’UTR-mut). The luciferase activity was measured and normalized to renilla luciferase activity (upper panel). HEK293 cells were transfected with NC or miR-122 for 48 h. MAVS expression were analyzed by western blotting (lower panel). (F) The finding that miR-122 regulating the luciferase activity of MARL is also examined in *N*. *diacanthus* and *L*. *crocea*. NLC cells were transfected with NC or miR-122, together with the wild type or mutated type of *N*. *diacanthus* MARL, then luciferase activity was analyzed and normalized to renilla luciferase activity (left panel). LLC cells were transfected with NC or miR-122, together with the wild type or mutated type of as well as *L*. *crocea* MARL, then luciferase activity was analyzed and normalized to renilla luciferase activity (right panel). (G) MARL reduces miR-122 activity in *N*. *diacanthus* and *L*. *crocea*. NLC cells or LLC cells were respectively transfected with *N*. *diacanthus* or *L*. *crocea* MARL expression plasmid, together with miR-122 sensor, miR-122 and pcDNA3.1 vector for 48 h. The luciferase activity was analyzed and normalized to renilla luciferase activity. All data represented the mean ± SE from three independent triplicated experiments. *, *p* < 0.05.

To address the generality of our findings, we first examined the sequence alignment of pre-miR-122 from different vertebrate species. Interestingly, as shown in Fig 9A, mature miR-122 displayed a high conservation from mammals to fish. Further, the miR-122-binding site in MAVS 3’UTR also displayed a high conservation from mammals to fish (Fig 9B). To obtain the direct evidence that miR-122 could target MAVS 3’UTR across species, luciferase reporter genes were generated by cloning MAVS 3’UTR of zebrafish (*Danio rerio*), large yellow croaker (*Larimichthys crocea*), and human (*Homo sapiens*) into pmirGLO vector, within the mutant devoid of miR-122 binding site as a control. Strikingly, miR-122 mimics were sufficient to decrease luciferase activities when respectively cotransfected with the wild types of *D*. *rerio* MAVS 3’UTR, *L*. *crocea* MAVS 3’UTR, and *H*. *sapiens* MAVS 3’UTR, whereas it showed no effect on the luciferase activity of cells transfected with their mutant-types (Fig 9C–9E, upper panel). Further, we have also proved that miR-122 target MAVS gene in the indicated species (Fig 9C–9E, lower panel). These results indicate that miR-122 could target MAVS gene in other vertebrates, including humans, which verify that miR-122 is highly conserved among different vertebrate groups, and its function is also conserved to some extent.

Additionally, we also verified the findings that miR-122 regulating long noncoding RNA MARL also exist in other species. First, we examined the sequence alignment of MARL among different species. Strikingly, the sequence of MARL is highly conserved among different fish species. Particularly, MARL in these different species presents highly conserved in the binding sites of miR-122 (S5 Fig). Then, to examine whether MARL in other species could also interact with miR-122, we produced luciferase constructs of *Nibea diacanthus* and *L*. *crocea* MARL, and their mutated forms with miR-122 binding sites mutated. Luciferase assays reveled that miR-122 could suppress the luciferase activity of the wild form of MARL luciferase plasmid in both species, but it had no effect on mutated forms (Fig 9F). Furthermore, to test whether *N*. *diacanthus* and *L*. *crocea* MARL can affect miR-122 activity, we conducted luciferase assays and found that both *N*. *diacanthus* and *L*. *crocea* MARL could counteract the inhibitory effect of miR-122 on miR-122 sensor (Fig 9G). These results indicate that MARL may act as endogenous sponge RNA to interact with miR-122 among different teleost fish. However, the corresponding sequence of MARL has not found in mammals, indicating the function of MARL is relatively conserved and may not widely exist in vertebrates.

## Discussion

Host defense against viral invasion requires induction of appropriate immune responses. To achieve the appropriate immune response, the antiviral signaling pathways are tightly controlled by a multistep regulatory mechanism and distinct genes. In other side, viruses have developed a myriad of regulation strategies for their evading and subverting of host defenses. Thus, various layers of regulators and mechanisms networks are needed to ensure maintenance of viral clearance and host preservation. Herein, we reported an interaction network regulating teleost MAVS-mediated antiviral signaling pathways. We found that fish MAVS acts as a crucial signaling molecule in the infection of SCRV, which mediated both the NF-κB and IRF3 activation and lead to type I IFNs and inflammatory cytokine production. miR-122 can reduce the expression of MAVS and suppress MAVS-mediated antiviral responses, which may help viruses evade host antiviral responses. We further proved that lncRNA MARL acts as endogenous sponge RNA to interact with miR-122 and facilitate MAVS expression, thus enhancing the antiviral signaling pathways. In short, MARL can counteract an increasing effect of miR-122 on SCRV replication, thus maintaining the stable of antiviral responses and ensuring appropriate inflammatory responses.

The host signaling protein MAVS is essential to drive antiviral innate immunity in response to RNA virus infection [26]. MAVS-mediated antiviral signaling pathways initiates after RIG-I and MDA5 sense viral RNA. This initiation of signaling drives interactions between the RLRs, MAVS, and corresponding proteins and organelles, which mediates the activation of NF-κB and IRF3 and leads to type I IFNs production. Because the essential role of MAVS in the antiviral innate immunity, the mechanisms and regulations of MAVS-mediated signaling have been extensively studied. It has been reported that several regulatory proteins, such as NLRX1 [27], RNF5 [28], MFN1 [29], MFN2 [30] can negatively or positively regulate the MAVS-mediated antiviral signal transduction. Similar to mammals, fish possess conserved immune-relevant genes and a series of signaling events in response to invading pathogens. For instance, teleost fish could initiate the evolutionarily conserved interferon system in responds to RNA virus infection [31]. Fish genomes have the orthologous genes involved in the RLR signaling pathway, such as RIG-I, MDA-5, MAVS, MITA, TBK-1 and IRF3/7 [24, 32]. However, whether teleost fish utilize a conserved signaling pathway involving in MAVS-mediated IFN response and the related regulatory networks remains elusive. Here, we extended the notion that fish MAVS is involved in the RLR-triggered IFN signaling and provided evidence that similar to mammal MAVS, fish MAVS mediates the activation of NF-κB and IRF3 and leads to type I IFNs production in response to RNA virus infection. Further investigations showed that ncRNAs, including miR-122 and MARL, plays critical regulatory roles in MAVS-mediated singling pathway.

miRNAs function primarily by binding to the 3’UTR of target mRNAs to achieve posttranscriptional regulation of gene expression. In mammals, miRNAs have been regarded as important and versatile modulators of innate immunity and intricate networks of host-pathogen interactions. For example, miR-29b has been shown as a potent regulator of JEV-induced neuroinflammation by the targeting of TNFAIP3 in microglia cells [12]. microRNA-146a feedback inhibits RIG-I-dependent type I IFNs production by targeting TRAF6, IRAK1, and IRAK2 in macrophages [33]. From the first report of miRNAs in zebrafish [34, 35], the role of miRNAs as fine-tuning regulators of different biological processes have been clarified in teleost fish. Among them, some miRNAs have been proposed constitute key switches for activating or inhibiting of immune responses. Teleost pol-miR-731, upregulated by megalocytivirus, has been examined to involve in virus-induced type I interferon response [36]. Rhabdovirus-inducible miR-210 has been reported to modulate antiviral innate immune response via targeting STING/MITA in miiuy croaker [15]. Miiuy croaker miR-3570 has been showed to inhibit the production of type I IFN and inflammatory cytokine by targeting MAVS, thereby promoting viral replication [16]. Individual miRNAs have been thought to regulate multiple targets using different binding regions, meanwhile, one gene can be regulated by multiple miRNAs. Thus, miRNAs and mRNAs can build complicated networks. In the present study, miR-122 was proved to be a novel miRNA targeting MAVS in miiuy croaker. miR-122 negatively regulates MAVS expression and suppresses MAVS-mediated IFNs production, thereby promoting SCRV replication. The negative regulation mechanism may be a strategy of virus for their survival by escaping the antiviral immune response of host. Given the important roles of the MAVS-mediated signaling pathway in the innate antiviral immune response, identifying more miRNAs that can regulate IFNs production will be an interesting and vital important future work.

It is well known that the genomes of eukaryote encode a surprisingly large number of non-coding transcripts. As reported, around two-thirds of mammalian transcripts are non-coding [37]. Non-coding RNAs (ncRNAs), such as miRNAs, lncRNAs, and circular RNAs have recently gained significant attention in gene regulation. Generally, miRNAs are highly conserved among different vertebrate groups, and its functions are also conserved to some extent. For instance, the conservative discovery of let-7 from human to nematodes aroused great interest in miRNAs [38] Subsequent comparative analysis has played a crucial role in identifying miRNA genes, predicting miRNA targets in mRNAs, and revealing features that are important for miRNA biogenesis. In this study, our present work reveals that miR-122 displays a high conservation from mammals to fish (Fig 9A). Further investigations showed that the function of miR-122 that targets MAVS and regulates its expression exist in several fish species, as well as in human, which verify that miR-122 is highly conserved among different vertebrate groups, and its function is also conserved to some extent (Fig 9C–9E). This findings may provide new insights into understanding intricate miRNA networks in vertebrates. With regarding to lncRNAs, they usually lack strong conservation, and many well-described mammals lncRNAs, like Xist, are poorly conserved [39]. Remarkably, recent studies have revealed that lncRNAs present evolutionary characteristics of functionality; these lncRNAs could evolve under moderate but detectable negative selective pressure, accumulating fewer substitutions than expected for neutrally evolving sequences [40]. This evolutionary feature is very important for preserving their function, and the conserved non-coding RNAs are expected to facilitate biological processes that are common to many different lineages [40]. In the present study, we examined the sequence of MARL and found the full length of MARL is conserved across several fish species, including *M*. *miiuy*, *L*. *crocea*, *N*. *diacanthus*, and *Sciaenops ocellatus* (S5 Fig). As comparative analysis of genes across species can be a powerful tool for studying their functions, the highly conservation of MARL sequence indicated the conservation of their function, suggesting an essential and irreplaceable role of lncRNA in biological processes of teleost fish.

**Fig 10:**
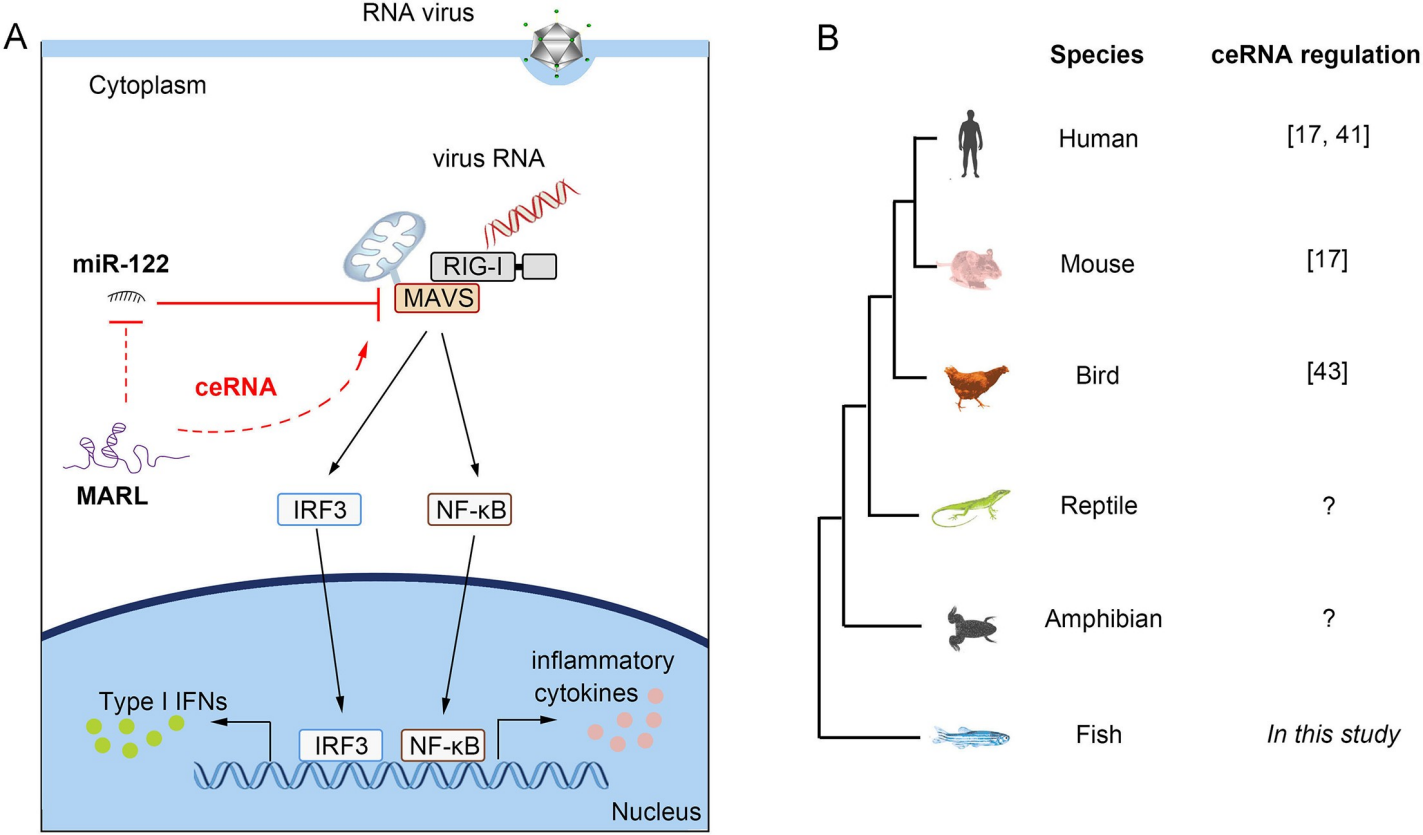
The mechanism graph of the regulatory network and function of MAVS. (A) Fish MAVS could induce the innate antiviral responses through recruiting NF-κB and IRF3 to trigger IFNs activation upon RNA viral infection. miR-122 targets MAVS and represses MAVS-mediated antiviral responses, thereby promoting viral replication. MARL act as a molecular sponge regulating miR-122 to enhance MAVS expression, thereby maintaining the stable of antiviral responses and ensuring appropriate inflammatory responses. (B) The ceRNA regulatory networks may exist wildly in vertebrate species.

Growing number of reports suggest that lncRNAs can operate as ceRNAs to regulate protein-coding genes in mammals. An example of such regulation is exemplified by HULC, an lncRNA upregulated in liver cancer, whose upregulated expression is in part to its inhibitory effects on the expression and activity of miR-372 [41]. Similar report suggest that a muscle-specific lncRNA, linc-MD1, governs the time of muscle differentiation by acting as a ceRNAs in mouse and human myoblasts [17]. Meanwhile, researchers showed that H19 acts as an endogenous sponge to regualte let-7 availability and inhibits muscle differentiation via antagonizing let-7 [42]. More recently, studies suggest a ceRNA regulatory network exists in birds [43]. Ma et al. revealed that lncRNA-Six1 functions as a ceRNAs by sponging miR-1611 to activate Six1 expression and fiber type switching in chicken myogenesis. However, it is still unclear whether ceRNA regulatory networks exist in reptiles, amphibians or lower vertebrate, like fish. Here, we showed that a lncRNA MARL, which was upregulated upon SCRV infection, can modulate MAVS expression through competitively sponging miR-122 in lower vertebrate, miiuy croaker (Fig 10A). This result is the first study for understanding of molecular regulation of lncRNA in teleost fish, which will provide theoretical support for lncRNA being responsible for regulating protein-encoding genes in lower vertebrates. As shown above, although it has not been reported that ceRNA regulatory networks exist in reptiles and amphibians, we speculate that ceRNA regulatory networks exist widely among all vertebrate groups as the discovery of ceRNA in fish in our study (Fig 10B).

In summary, we reported a regulatory network for fish MAVS in response to RNA virus infection. We observed that fish MAVS plays vital roles in antiviral innate immunity. Similar to mammals MAVS, MAVS in teleost fish mediates the activation of NF-κB and IRF3, leading to type I IFNs production. miR-122 plays a negative role, while MARL exhibited a positive regulatory role in the MAVS-mediated antiviral responses. We further identified that MARL could act as ceRNA for miR-122 to relieve its repressive effects on MAVS expression, thus inhibiting virus replication. Our findings suggest the critical role of lncRNAs in operating fish biological processes, and this is the first study to show ceRNA networks existing in lower vertebrates, which will benefit for understanding vertebrate immunology and the evolution of immune systems among vertebrates.

## Methods

### Ethics statement

All animal experimental procedures were performed in accordance with the National Institutes of Health’s Guide for the Care and Use of Laboratory Animals, and the experimental protocols were approved by the Research Ethics Committee of Shanghai Ocean University (No. SHOU-DW-2018-047).

### Sample and challenge

Miiuy croaker (∼ 50 g) was obtained from Zhoushan Fisheries Research Institute, Zhejiang Province, China. Fish was acclimated in aerated seawater tanks at 25°C for six weeks before experiments. *Siniperca chuatsi rhabdovirus* (SCRV) challenge was performed as follows. Briefly, fish was challenged with 200 μl SCRV at a multiplicity of infection (MOI) of 5 through intraperitoneal. As a comparison, 200 μl of physiological saline was used to challenge the individuals. Afterwards, fishes were respectively sacrificed at different time point and the intestinal tissues were collected for RNA extraction.

### Cell culture and treatment

*M*. *miiuy* intestinal cells (MIC), and *M*. *miiuy* brain cells (MBrC), *L*. *crocea* liver cells (LLC), and *N*. *diacanthus* liver cells (NLC) were cultured in L-15 medium (HyClone) supplemented with 15% fetal bovine serum (FBS; Gibco), 100 U/ml penicillin, and 100μg/ml streptomycin at 26°C. Zebrafish liver cells (ZFL) were cultured in 50% L-15 medium (HyClone), 35% DMEM-HG (HyClone), and 15% Ham’s F12 medium (HyClone) supplemented with 0.15 g/l sodium bicarbonate (Sigma-Aldrich), 15 mM HEPES (Sigma-Aldrich), and 10% FBS at 28°C in 5% CO_2_. EPC cells were maintained in medium 199 (Invitrogen) supplemented with 10% FBS, 100 U/ml penicillin, and 100 mg/ml streptomycin at 28°C in 5% CO_2_. HEK293 cells were cultured in DMEM (HyClone) supplemented with 10% FBS, 100 U/ml penicillin, and 100 mg/ml streptomycin at 37°C in 5% CO_2_. For stimulation experiments, MIC cells and MBrC that were susceptible to SCRV infection were challenged with SCRV at a multiplicity of infection (MOI) of 5 and harvested at different times for RNA extraction.

### Sequencing analysis and lncRNAs identification

To identify lncRNAs that are potentially involved in the regulation of SCRV infection, we treated miiuy croaker with SCRV, then used Illumina HiSeq 2500 platform to compare lncRNA expression levels between SCRV treated and untreated spleen samples. Briefly, all transcripts longer than 200 bp were subjected to protein coding potential evaluation by CPC [25], CNCI [44] and CPAT [45] software and Pfam [46] database which distinguish coding and noncoding transcripts with high accuracy. The transcripts were then screened as lncRNAs with Cuffmerge Software, and the conditions were as follows: the number of exon ≥ 2, length > 200 bp, FPKM ≥ 0.5, and to eliminate overlapping and coding potential transcripts with known coding transcripts [47]. Overall, a total of 8942 lncRNAs have been identified. After testing differentially expressed transcripts, 897 lncRNAs has shown differentially expression, and MARL is one of the significantly ones.

To characterize the complete sequence of lncRNAs, single-molecule full-length transcript sequencing (Iso-Seq) were used. Briefly, ten tissues of miiuy croaker, including the liver, spleen, kidney, intestine, muscle, brain, heart, fin, skin, and stomach were mixed and divided into two groups for constructing SMRAT Library, respectively. After combining the data, 73,347 full-length transcripts have been identified. Then four software or database, including CPC, CNCI, CPAT, and Pfam were used to discard sequences with coding potential, and a total of 13091 full-length lncRNAs were obtained. The length of MARL was obtained by searching the full-length transcripts.

### Plasmids construction

To construct the MAVS 3’UTR reporter vector, the 3’UTR region of *M*. *miiuy* MAVS gene (GenBank accession no. MF871620), as well as *D*. *rerio*, *L*. *crocea*, and *H*. *sapiens* MAVS 3’UTR (Ensemble database), were amplified using PCR and cloned into pmirGLO luciferase reporter vector (Promega). To construct MARL luciferase genes, the sequences of MARL in *M*. *miiuy*, *N*. *diacanthus*, and *L*. *crocea* were cloned into pmirGLO luciferase reporter vector, respectively. The mutated forms with point mutations in the miR-122 binding site were synthesized using Mut Express II Fast Mutagenesis Kit V2 with specific primers (S1 Table). Meanwhile, the sequences of MAVS 3’UTR and MARL were inserted into the mVenus-C1 (Invitrogen), which included the sequence of enhanced GFP. To construct the MAVS expression plasmid, the full length of coding sequence region and 3’UTR of MAVS gene were amplified by specific primer pairs and cloned into pcDNA3.1 vector (Invitrogen). Also, MARL expression plasmids were constructed by cloning MARL sequence region of *M*. *miiuy*, *N*. *diacanthus*, and *L*. *crocea* into pcDNA3.1 vector. To build pcDNA3.1-MS2, the MS2-12X fragment was inserted into the *BamH* I and *EcoR* V restriction sites of pcDNA3.1 vector, and then the MARL was amplified and cloned into pcDNA3.1-MS2. The mutated forms with point mutations in the miR-122 binding site were synthesized using Mut Express II Fast Mutagenesis Kit V2 with specific primers (S1 Table). A miR-122 sensor was created by inserting two consecutive miR-122 complementary sequences into psiCHECK vector (Promega). The correct construction of the plasmids was verified by Sanger sequencing and extracted through EndoFree Plasmid DNA Miniprep Kit (Tiangen Biotech).

### Cell transfection

Transient transfection of cells with miRNA mimic, miRNA inhibitor or siRNA was performed in 24-well plates using Lipofectamine^TM^ RNAiMAX (Invitrogen), and cells were transfected with DNA plasmids was performed using Lipofectamine^TM^ 3000 (Invitrogen) according to the manufacturer’s instructions. For functional analyses, the expression plasmid (500 ng per well) or empty plasmid (500 ng per well) and miRNA mimics (100 nM), miRNA inhibitor (100nM) or siRNA (100nM) were transfected into cells in culture medium and then harvested for further detection. For luciferase experiments, miRNA mimics (100 nM) or miRNA inhibitor (100nM) and pmirGLO (500 ng per well) containing the wild or mutated plasmid of MAVS 3’UTR or MARL were transfected into cells.

### RNA extract and quantitative real-time PCR

The viral RNA in the intracellular and supernatant was extracted by using the Body Fluid Viral DNA/RNA Miniprep Kit (Axygen). For the isolation and purification of both cytoplasmic and nuclear RNA from MIC cells the Norgen’s Cytoplasmic & Nuclear RNA Purification Kit (Norgen Biotek, Cat.21000) has been used according to the manufacturer’s instructions. Total RNA was isolated with TRIzol Reagent (Invitrogen) and the cDNA was synthesized using the FastQuant RT Kit (Tiangen) which includes DNase treatment of RNA to eliminate genomic contamination. The expression patterns of each gene were performed by using SYBR Premix Ex TaqTM (Takara). The small RNA was extracted by using miRcute miRNA Isolation Kit (Tiangen), and miRcute miRNA FirstStrand cDNA Synthesis Kit (Tiangen) was applied to reverse transcription of miRNAs. The expression analysis of miR-122 was executed by using the miRcute miRNA qPCR Detection Kit (Tiangen). Real-time PCR was performed in a Applied Biosystems QuantStudio 3 (Thermo Fisher Scientific). GAPDH and 5.8S rRNA were employed as endogenous controls for mRNA/lncRNA and miRNA, respectively. Primer sequences are displayed in S1 Table.

### RNA oligoribonucleotides

The miR-122 mimics are synthetic double-stranded RNAs (dsRNAs) with stimulating naturally occurring mature miRNAs. The miR-122 mimics sequence was, 5’-UGGAGUGUGACAAUGGUGUUUG-3’. The negative control mimics was 5’-UUCUCCGAACGUGUCACGUTT-3’. miRNA inhibitors are synthetic single-stranded RNAs (ssRNAs) that sequester intracellular miRNAs and block their activity in the RNA interfering pathway. The miR-122 inhibitors sequence was 5’-CAAACACCAUUGUCACACUCCA-3’. The negative control inhibitors was 5’-CAGUACUUUUGUGUAGUACAA-3’.

The MAVS RNA interference sequence was 5’- GAUGAACGUGGUGCAGAUATT-3’. The RNA interference sequence for MARL was 5’-AUGACAUCACGCCUCCAAATT-3’. The scrambled control RNA sequences were 5’- UUCUCCGAACGUGUCACGUTT-3’.

### Dual-luciferase reporter assays

The MARL wild type and the mutant devoid of miR-122 binding site was contransfected with miR-122 mimics into EPC cells. At 48 h post-transfection, reporter luciferase activities were measured using the Dual-Luciferase reporter assay system (Promega). To determine the functional regulation of MAVS or MARL, MIC cells were cotransfected MAVS expression plasmid or MARL expression plasmid, together with NF-κB, IRF3, IFN-1, and IFN-2 luciferase reporter gene plasmids, pRL-TK Renilla luciferase plasmid, either miR-122 mimics or negative controls. At 48 h post-transfection, the cells were lysed for reporter activity using the Dual-Luciferase reporter assay system (Promega). miR-122 sensor was cotransfected with miR-122 mimics or MARL expression plasmid into MIC cells. At 48 h post-transfection, the cells were lysed for reporter activity. All the luciferase activity values were achieved against the renilla luciferase control. Transfection of each construct was performed in triplicate in each assay. Ratios of renilla luciferase readings to firefly luciferase readings were taken for each experiment, and triplicates were averaged.

### Western blotting

Cellular lysates were generated by using 1 × SDS-PAGE loading buffer. Proteins were extracted from cells and measured with the BCA Protein Assay kit (Vazyme), then subjected to SDS-PAGE (10%) gel and transferred to PVDF (Millipore) membranes by semidry blotting (Bio-Rad Trans Blot Turbo System). The membranes were blocked with 5% BSA. Protein was blotted with different antibodies. The antibody against MAVS was diluted at 1: 500 (Abcam); anti-Flag and anti-Tubulin monoclonal antibody were diluted at 1: 2,000 (Sigma); the anti-GFP monoclonal antibody was diluted at 1: 1,000 (Sigma); and HRP-conjugated anti-rabbit IgG or anti-mouse IgG (Abbkine) at 1: 5,000. The results were the representative of three independent experiments. The immunoreactive proteins were detected by using WesternBright^TM^ ECL (Advansta). The digital imaging was performed with a cold CCD camera.

### RNA pulldown assay

MARL and MARL-mut with miR-122 binding sites mutated were transcribed *in vitro*. The two transcripts were biotin-labeled with the T7 RNA polymerase and Biotin RNA Labeling Mix (Roche), treated with RNase-free DNase I, and purified with an RNeasy Mini Kit (Qiagen). The whole-cell lysates from MIC cells (~1.0 × 10^7^) was incubated with purified biotinylated transcripts for 1 h at 25°C. The complexes were isolated by streptavidin agarose beads (Invitrogen). RNA was extracted from the remaining beads and qPCR was used to evaluate the expression levels of miRNAs.

To conduct pulldown assay with biotinylated miRNA, MIC cells was harvested at 48 h after transfection, then incubated on ice for 30 min in lysis buffer (20 mM Tris, pH 7.5, 200mM NaCl, 2.5 mM MgCl_2_, 1mM DTT, 60 U/ml Superase-In, 0.05% Igepal, protease inhibitors). The lysates were precleared by centrifugation for 5 min, and 50 μl of the sample was aliquoted for input. The remaining lysates were incubated with M-280 streptavidin magnetic beads (Sigma). To prevent non-specific binding of RNA and protein complexes, the beads were coated with RNase-free BSA and yeast tRNA (both from Sigma). The beads were incubated for 4 h at 4°C, washed twice with ice-cold lysis buffer, three times with the low salt buffer (0.1% SDS, 1% Trition X-100, 2 mM EDTA, 20 mM Tris-HCl pH 8.0 and 150 mM NaCl) and once with the high salt buffer (0.1% SDS, 1% Trition X-100, 2 mM EDTA, 20 mM Tris-HCl pH 8.0 and 500 mM NaCl) [48]. RNA was extracted from the remaining beads with TRIzol Reagent (Invitrogen) and evaluated by qPCR.

### RNA immunoprecipitation

MIC cells (~2.0 × 10^7^) were cotransfection with pcDNA3.1-MS2, pcDNA3.1-MS2-MARL, pcDNA3.1-MS2-MARL-mut, pcDNA3.1-MS2-MAVS-3’UTR, pcDNA3.1-MS-MAVS-3’UTR-mut or pMS2-GFP (Addgene). To construct plasmids that could produce lncRNAs or MAVS-3’UTR identified by the MS2 protein, an MS2-12X fragment was cloned into pcDNA3.1, pcDNA3.1-MARL, the mutated type of MARL plasmid or pcDNA3.1-MAVS-3’UTR and the mutated type of MAVS-3’UTR plasmid. Furthermore, a GFP and MS2 gene fusion expression plasmid was also constructed to produce a GFP-MS2 fusion protein that could bind with the MS2-12X fragment and be identified using an anti-GFP antibody. After 48 h transfection, the MIC cells were used in RIP assays via the Magna RIP^TM^ RNA-Binding Protein Immunoprecipitation Kit (Millipore) and an anti-GFP antibody (Abcam) following the manufacturer’s protocol. RNA was extracted from the remaining beads and qPCR was used to evaluate the expression levels of miRNAs.

### Cell viability and proliferation

Cell viability was measured at 72 h after transfection in SCRV-treated MIC with CellTiter-Glo Luminescent Cell Viability assays (Promega) according to the manufacturer’s instructions. Cell proliferation assays were performed with BeyoClick EdU cell Proliferation Kit with Alexa Fluor 488 (Beyotime) following the manufacturer’s instructions. All the experiments were performed in triplicate.

### Statistical analysis

Data are expressed as the mean ± SE from at least three independent triplicated experiments. Student’s t-test was used to evaluate the data. The relative gene expression data was acquired using the 2 ^ΔΔCT^ method, and comparisons between groups were analyzed by one-way analysis of variance (ANOVA) followed by Duncan’s multiple comparison tests [49]. A value of *p* < 0.05 was considered significant.

## Supporting information

S1 Table PCR primer information in this study. (DOCX)

S1 Fig miR-122 target fish MAVS. (A) The time gradient experiment was conducted for transfection of miR-122 mimics. Luciferase activity was normalized to renilla luciferase activity. (B) The miR-122 (0, 30, 60, and 90 nM) together with NC (90, 60, 30, and 0 nM) were cotransfected with MAVS-3’UTR-wt into EPC cells. At 48 h post-transfection, the luciferase activity was determined. Luciferase activity was normalized to renilla luciferase activity. (C and D) miR-122 could downregulate GFP expression. EPC cells were cotransfected with the wild type of mVenus-MAVS-3’UTR or the mutated type of mVenus-MAVS-3’UTR, together with NC or miR-122. At 48 h post-transfection, the fluorescence intensity (C) and the GFP expression levels (D) were evaluated by enzyme-labeled instrument and western blotting, respectively. Scale bar, 20 μm; original magnification × 10. (E) The schematic diagram of RIP method to identify the binding between MAVS-3’UTR and miR-122. (F) The qPCR results of the MS2-RIP method used to identify the binding between MAVS-3’UTR and miR-122 in MIC cells. The qPCR for miR-122, as well as negative control miR-217, was performed after RIP process. (G) miR-122 regulate MAVS expression. EPC cells were cotransfected with MAVS expression plasmid, along with miR-122 or NC. At 48 h post-transfection, MAVS expression were determined by western blotting. All data represented the mean ± SE from three independent triplicated experiments. *, *p* < 0.05.
(TIF)

S2 Fig SCRV induces the expression of miR-122. The expression levels of miR-122 in MIC cells (A) and intestine samples (B) were measured by qPCR at 24 h after SCRV infection. All data represented the mean ± SE from three independent triplicated experiments. *, *p* < 0.05.
(TIF)

S3 Fig miR-122 regulates the luciferase activity of MARL. (A) The time gradient experiment was conducted for transfection. (B) The miR-122 (0, 30, 60, and 90 nM) together with NC (90, 60, 30, and 0 nM) were cotransfected with Luc-MARL-wt into EPC cells. At 48 h post-transfection, the luciferase activity was determined. Luciferase activity was normalized to renilla luciferase activity. (C) MIC cells were transfected with the biotinylated wild type of miR-122 (Bio-miR-122-wt) or the biotinylated mutated type of miR-122 (Bio-miR-122-mut) for 48 h. Cells were harvested for biotin-based pulldown assay. The expression of negative control, non-targeted lncRNAs (LTCONS_00021456) was analyzed by qPCR. (D) MIC lysates were incubated with biotin-labeled MARL and MARL-mut. The qPCR for negative control, other non-interacted miRNAs (miR-217) was performed after pull down process. (E) The qPCR results of the MS2-RIP method was conducted to test the expression of negative control, other non-interacted miRNAs (miR-217). All data represented the mean ± SE from three independent triplicated experiments. *, *p* < 0.05.
(TIF)

S4 Fig MARL affect the MAVS-mediated signaling pathways. (A) MARL overexpression induced the luciferase activity under the transfection of wild-type MAVS 3’UTR. MIC cells were transfected with pcDNA3.1 vector or MARL expression plasmid, together with MAVS 3’UTR luciferase reporter genes for 48 h. Luciferase activity was analyzed and normalized to renilla luciferase activity. (B) MARL affect the expression of endogenous MAVS. MIC cells were tranfected with pcDNA3.1 vector or MARL expression plasmid for 48 h. MAVS expression were analyzed by western blotting. (C) MARL affects MAVS-mediated signaling. MIC cells were cotransfected with pRL-TK Renilla luciferase plasmid, luciferase reporter genes, pcDNA3.1 vector or MARL expression plasmid, together with MAVS expression plasmid for 48 h. The luciferase activity was measured and normalized to renilla luciferase activity. All data represented the mean ± SE from three independent triplicated experiments. *, *p* < 0.05.
(TIF)

S5 Fig The sequence of MARL gene in different fish species. miR-122 binding sites are shown in boxes.
(TIF)
