# Correction: Long noncoding RNA MARL regulates antiviral responses through suppression miR-122-dependent MAVS downregulation in lower vertebrates

**DOI:** 10.1371/journal.ppat.1013059

**Published:** 2025-04-07

**Authors:** The PLOS Pathogens Editors

Following publication of this article [1], questions were raised about similarity between this work and other studies by the same group, including about similarities in data reported for efficiency of miRNA knockdown by different oligonucleotide inhibitors. An investigation by Shanghai Ocean University found that the data reported in [1] are reliable and valid, and that similarities may have arisen because screening to identify functional molecules was carried out on a large scale simultaneously, with strict standards and thresholds set for taking forward further investigation of specific molecules.

The *PLOS Pathogens* Editors consider the above issue resolved, and issue this Correction to address additional items as follows:

Contrary to the Data Availability statement accompanying this article [1], the complete underlying data were not made available at the time of publication. The available individual-level quantitative data are provided in S1 File with this notice. Raw qPCR data are no longer available. The available underlying image data are provided in S2 File with this notice.

There is an error in Fig 1. The incorrect image was used in the left-hand si-Ctrl panel of Fig 1G, which is a duplicate of the left-hand MARL panel of Fig 7E; a revised Fig 1 is provided in which this panel is replaced with the correct image from the original experiment, which has been verified in the investigation by Shanghai Ocean University.

**Fig 1:**
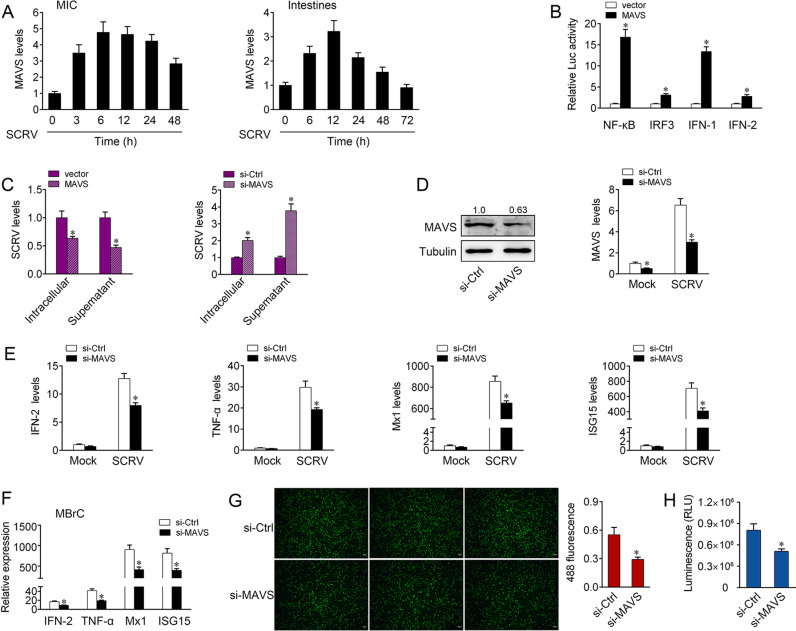
Fish MAVS suppresses antiviral responses upon SCRV infection. (A) SCRV induces an increase of MAVS expression. The expression levels of MAVS in MIC cells and intestine samples were measured by qPCR at indicated time after SCRV infection. (B) MAVS is able to activate NF-κB, IRF3, IFN-1, and IFN-2 signaling. MIC cells were transfected with pRL-TK Renilla luciferase plasmid, luciferase reporter genes, together with MAVS expression plasmid. At 48 h post-transaction, the luciferase activity was measured and normalized to renilla luciferase activity. (C) Fish MAVS suppresses SCRV replication. MIC cells were transfected with pcDNA3.1 vector or MAVS expression plasmid and control siRNA (si-Ctrl) or MAVS-specific siRNA (si-MAVS) for 48 h, then infected with SCRV. The qPCR analysis was conducted for intracellular and supernatant SCRV RNA expression. (D) Knockdown of MAVS attenuates the expression of endogenous MAVS. MIC cells were transfected with si-Ctrl or si-MAVS for 48 h, then the expression levels of MAVS were determined by western blotting and qPCR assays, respectively. (E) Knockdown of MAVS attenuates the expression of antiviral genes. MIC cells were transfected with si-Ctrl or si-MAVS. At 48 h post-transfection, cells were then treated with SCRV for 24 h. The expression of IFN-2, TNF-α, Mx1, and ISG15 were determined by qPCR. (F) MBrC cells were transfected with si-Ctrl or si-MAVS. At 48 h post-transfection, cells were then treated with SCRV for 24 h. The expression of IFN-2, TNF-α, Mx1, and ISG15 were determined by qPCR. (G and H) Effect of MAVS knockdown on cell proliferation and viability after SCRV infection. MIC cells were transfected with either si-MAVS or si-Ctrl. At 48 h post-transfection, the cells were infected with SCRV for 24 h, then cell proliferation assay (G) and cell viability assay (H) were measured. Scale bar, 20 μm; original magnification × 10. All data represented the mean ± SE from three independent triplicated experiments. *, *p* < 0.05.

There is an error in Fig 6. The Tubulin western blot panel in Fig 6D is incorrect and is a duplicate of the Tubulin panel in Figure 5F of [2]. A revised Fig 6 is provided in which the Tubulin panel is replaced with the correct western blot image from the original experiment, which has been verified in the investigation by Shanghai Ocean University.

**Fig 6:**
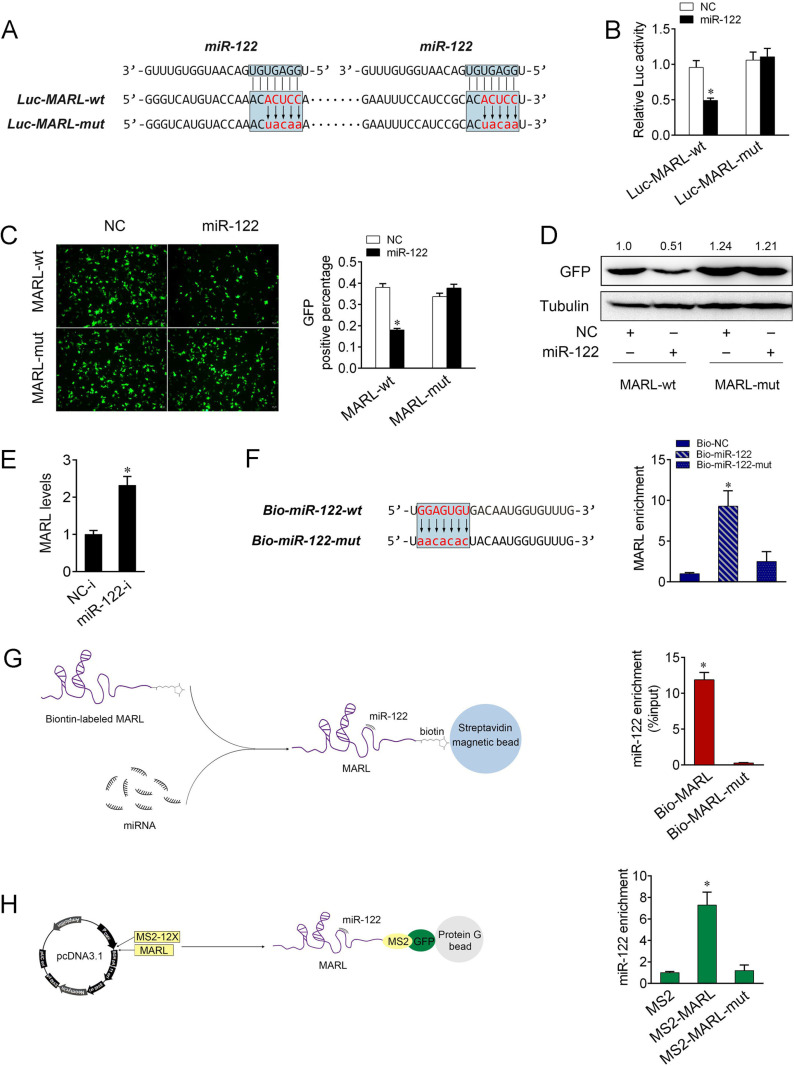
miR-122 interacts with MARL. (A) MARL sequence contains two sites complementary to miR-122. miR-122 binding sites in MARL wild-type form (Luc-MARL-wt) and the mutated form (Luc-MARL-mut) were shown. (B) EPC cells were transfected with NC or miR-122, together with Luc-MARL-wt or Luc-MARL-mut. At 48 h post-transaction, the luciferase activity was analyzed and normalized to renilla luciferase activity. (C and D) MARL could downregulate GFP expression. EPC cells were cotransfected with the wild type of mVenus-MARL or the mutated type, together with NC or miR-122. At 48 h post-transfection, the fluorescence intensity (C) and the GFP expression (D) were evaluated by enzyme-labeled instrument and western blotting, respectively. Scale bar, 20 μm; original magnification × 10. (E) MIC cells were transfected with NC-i or miR-122-i for 48 h. The expression of MARL were measured by qPCR. (F) The wild and mutated forms of biotinylated miR-122 sequence were shown (left panel). MIC cells were transfected with the biotinylated wild type of miR-122 (Bio-miR-122-wt) or the biotinylated mutated type of miR-122 (Bio-miR-122-mut) for 48 h. Cells were harvested for biotin-based pulldown assay. MARL expression were analyzed by qPCR (right panel). (G) The schematic diagram of the RNA pull down method to identify the binding between MARL and miR-122 (left panel). MIC lysates were incubated with biotin-labeled MARL and MARL-mut. miRNA real-time PCR was performed after pull down process (right panel). (H) The schematic diagram of RIP method (left panel). The qPCR results of the MS2-RIP method used to identify the binding between MARL and miR-122 in MIC cells. miRNA real-time qPCR was performed after RNA immunoprecipitation process (right panel). All data represented the mean ± SE from three independent triplicated experiments. *, *p* < 0.05.

Editorial assessment of the underlying blot images identified that the published western blot panels in Fig 8E show tubulin loading control bands taken from different lanes compared to the corresponding MAVS bands. The corresponding author stated that the control and experimental blots were generated from different gels that differed in sample loading order.

The ethical approval document issued by the Research Ethics Committee of Shanghai Ocean University has been provided for editorial review, and a representative of Shanghai Ocean University has confirmed that the procedures reported in [1] are in accordance with this ethical approval and conducted within its period of validity.

## Supporting information

S1 File Individual-level quantitative data underlying charts and graphs.
(ZIP)

S2 File Underlying image data for Figures 1-9, S1, and S4.
(ZIP)
