# Supplementary material for: Long noncoding RNA MARL regulates antiviral responses through suppression miR-122-dependent MAVS downregulation in lower vertebrates
Source: PLoS Pathog. 2020 Jul 17;16(7):e1008670. doi: 10.1371/journal.ppat.1008670 (PMC7390449; doi:10.1371/journal.ppat.1008670)
Supplement: S1 Table — (DOCX) [file ppat.1008670.s001.docx]

**Table S1** PCR primer information in this study.

| **Primer name** | **Sequences (5’-3’)** |
| --- | --- |
| MAVS-qRT-F | AGGCACCAACAATTCCAG |
| MAVS-qRT-R | ACGGAGCAGGCTTCACTT |
| IFN-2-qRT-F | GCTCTGCCTTCCCTGCTA |
| IFN-2-qRT-R | CAGTTGACTCCGCCCTCT |
| TNF-α-qRT-F | GTTTGCTTGGTACTGGAATGG |
| TNF-α-qRT-R | TGTGGGATGATGATCTGGTTG |
| Mx1-qRT-F | GCTGCTTGTTTACTCCCA |
| Mx1-qRT-R | ACCTGCATCATCTCCCTC |
| ISG15-qRT-F | TGAACGGACAGAAGACGC |
| ISG15-qRT-R | TGAGGAATACCTGCATGG |
| MARL-qRT-F | GTCTAAGGCTTCGTATCGA |
| MARL-qRT-R | ATGACAACACGCTGACAAT |
| LTCONS_00025446-qRT-F | TACCGCCTTACACG |
| LTCONS_00025446-qRT-R | TACCGCCTTACACG |
| LTCONS_00034464-qRT-F | ATAGGTGTTCACGGTAGTTG |
| LTCONS_00034464-qRT-R | CATGTTGATGGGTTGGAG |
| LTCONS_00041598-qRT-F | GAATACCGCCTTACACGC |
| LTCONS_00041598-qRT-R | CAAATCACCGTGTCCC |
| LTCONS_00049719-qRT-F | TGCCACAACAATCAG |
| LTCONS_00049719-qRT-R | CCTTCTACAGCGTCTC |
| LTCONS_00021456-qRT-R | TGTCTTCAACTGCCCTTCC |
| LTCONS_00021456-qRT-R | ATCCTCCAAATGGTTCCAC |
| SCRV-qRT-F | GGGCTGGATGATAGACGATTG |
| SCRV-qRT-R | TGGCGGAGGTGCTTGATATGG |
| miR-122-qRT-F | CGAGTGGAGTGTGACAATGG |
| miR-122-qRT-F | CAGTTTTTTTTTTTTTTTCAAAC |
| miR-217-qRT-F | AGTACTGCATCAGGAACTGA |
| miR-217-qRT-F | CCAGTTTTTTTTTTTTTTTGCCAATC |
| 5.8S rRNA-qRT-F | AACTCTTAGCGGTGGATCA |
| 5.8S rRNA-qRT-R | GTTTTTTTTTTTTTTTGCCGAGTG |
| GAPDH-qRT-F | ACCTTCACTCCTCCATCTT |
| GAPDH-qRT-R | AGGTCACAGACACGGTTG |
| U6-qRT-F | TGCGAGTAGCAGACCA |
| U6-qRT-R | CACGAGACCGAAACAC |
| Tubulin-qRT-F | TACAGGTTCAGGGATGG |
| Tubulin-qRT-R | TGGTCAGCTTCAGGGT |
| MAVS-pcDNA3.1-HindIIIF | GACGATGACGACAAGAAGCTTTCGTCTGCCAAAGACAAACTGTA |
| MAVS-pcDNA3.1-EcoRIR | TGATGGATATCTGCAGAATTCCAGCCTCTGTCCTGTCTACTTCATG |
| MARL-pcDNA3.1-F | ACTATAGGGAGACCCAAGCTTAGGGGTCATGTACCAAACACTCC |
| MARL-pcDNA3.1-R | TGATGGATATCTGCAGAATTCACTTGCTAACAATGTTATCACTCTCTGTT |
| *Ndi*MARL-pcDNA3.1-HindIII-F | CCCAAGCTTGGGTCATGTACCAAAC |
| *Ndi*MARL-pcDNA3.1-EcoRI-R | CCGGAATTCGTCCAGAAATAGAGGC |
| *Lcr*MARL-pcDNA3.1-HindII-F | CCCAAGCTTGGGTCATGTACCAAAC |
| *Lcr*MARL-pcDNA3.1-EcoRI-R | CCGGAATTCGTCCAGAAATAGAGGC |
| MAVS-3’UTR-WT-NheIF | CTAGCTAGCCGTATGGTGCCTTATTG |
| MAVS-3’UTR-WT-XbaIR | TGCTCTAGACAGCCTCTGTCCTGTCTACT |
| MAVS-3’UTR-mut-F | CCAAATCTACCACACTGATTGTTAAGTTCTGGGTTGAGAT |
| MAVS-3’UTR-mut-R | TCAGTGTGGTAGATTTGGTGTCTGCTCGATCGG |
| *Dre*MAVS-3’UTR-WT-SacIF | CGCGAGCTCTGAGAATGTCTAACAGGCAC |
| *Dre*MAVS-3’UTR-WT-XbaIR | TGCTCTAGAAAGAACAGGAACAATCAAGA |
| *Dre*MAVS-3’UTR-mut-F | TTCAACCACATGGGTTCCTTGGTGGAAAACACA |
| *Dre*MAVS-3’UTR-mut-R | GGAACCCATGTGGTTGAAATATAACTGGGTAAACTGGC |
| *Lcr*MAVS-3’UTR-WT-XhoIF | CCGCTCGAGACCCTCCAGACCTTTGAT |
| *Lcr*MAVS-3’UTR-WT-SalIR | GTCGACGTCGACGCTCCTCGTTAATCCTCA |
| *Lcr*MAVS-3’UTR-mut-F | CCAAATCTACCACACTGATTTTAAGTTCTGGGTTCAGATC |
| *Lcr*MAVS-3’UTR-mut-R | TCAGTGTGGTAGATTTGGTGTGAGCTCGATCGG |
| *hsa*MAVS-3’UTR-WT-SacIF | CGAGCTCTGGCATTTACCAAGGG |
| *hsa*MAVS-3’UTR-WT-XhoIR | CCGCTCGAGGGCACAATCTCGGCTC |
| *hsa*MAVS-3’UTR-mut-F | CTTTTTCCACACACTGCCCTAGGGGAGTTCAGC |
| *hsa*MAVS-3’UTR-mut-R | GGCAGTGTGTGGAAAAAGCTGTAGCAGCCAAGG |
| MAVS-3’UTR-mVenus-HindIIIF | CCCAAGCTTGCTAGCGTATGGTGCCTTATTGG |
| MAVS-3’UTR-mVenus-BamHIR | CGCGGATCCCAGCCTCTGTCCTGTCTACT |
| MAVS-3’UTR-mVenus-mut-F | CCAAATCTACCACACTGATTGTTAAGTTCTGGGTTGAGAT |
| MAVS-3’UTR-mVenus-mut-R | TCAGTGTGGTAGATTTGGTGTCTGCTCGATCGG |
| MARL-pmirGLO-F | AACGAGCTCGCTAGCCTCGAGAGGGGTCATGTACCAAACACTCC |
| MARL-pmirGLO-R | CTTGCATGCCTGCAGGTCGACACTTGCTAACAATGTTATCACTCTCTGTT |
| MARL-pmirGLO-mut1-F | CCAAACTACAAACTAGAATGGTCAATGTGTGTTCACA |
| MARL-pmirGLO-mut1-R | TCTAGTTTGTAGTTTGGTACATGACCCCCTCG |
| MARL-pmirGLO-mut2-F | CCGCACTACAATACAGCTTGTGCATTTCTGTTTATCA |
| MARL-pmirGLO-mut2-R | GCTGTATTGTAGTGCGGATGGAAATTCCCTTC |
| *Ndi*MARL-pmirGLO-SacI-F | CGAGCTCGGGTCATGTACCAAAC |
| *Ndi*MARL-pmirGLO-XhoI-R | CCGCTCGAGGTCCAGAAATAGAGGC |
| *Ndi*MARL-pmirGLO-mut1-F | CCAAACTACAAACTAGAATGGTCAATGTGTGTTCACA |
| *Ndi*MARL-pmirGLO-mut1-R | TCTAGTTTGTAGTTTGGTACATGACCCCTCGAG |
| *Ndi*MARL-pmirGLO-mut2-F | CCGCACTACAATACAGCTTGTGCGTTTCTGTTTATC |
| *Ndi*MARL-pmirGLO-mut2-R | GCTGTATTGTAGTGCGGATGGAAATTCCTTCA |
| *Lcr*MARL-pmirGLO-SacI-F | CGAGCTCGGGTCATGTACCAAAC |
| *Lcr*MARL-pmirGLO-XhoI-R | CCGCTCGAGGTCCAGAAATAGAGGC |
| *Lcr*MARL-pmirGLO-mut1-F | CCAAACTACAAACTAGAATGGTCAATGTGTGTTCACA |
| *Lcr*MARL-pmirGLO-mut1-R | TCTAGTTTGTAGTTTGGTACATGACCCCTCTCG |
| *Lcr*MARL-pmirGLO-mut2-F | CCGCACTACAATACAGCTTGTGCGTTTCTGTTTATC |
| *Lcr*MARL-pmirGLO-mut2-R | GCTGTATTGTAGTGCGGATGGAAATTCCCTTC |
| MARL-mVenus-F | TCAGATCTCGAGCTCAAGCTTAGGGGTCATGTACCAAACACTCC |
| MARL-mVenus-R | CGGGCCCGCGGTACCGTCGACACTTGCTAACAATGTTATCACTCTCTGTT |
| miR-122-5p sensor-XhoIF | TCGAGACCTCACACTGTTACCACAAACACCTCACACTGTTACCACAAACGC |
| miR-122-5p sensor-NotIR | GGCCGCGTTTGTGGTAACAGTGTGAGGTGTTTGTGGTAACAGTGTGAGGTC |
| MARL-T7-F | TAATACGACTCACTATAGGGAGGGGTCATGTACCAAACACTCC |
| MARL-T7-R | ACTTGCTAACAATGTTATCACTCTCTGTT |
| pcDNA3.1-MS2-MARL-F | ACTATAGGGAGACCCAAGCTTAGGGGTCATGTACCAAACACTCC |
| pcDNA3.1-MS2-MARL-R | GCGGCCGTTACTAGTGGATCCACTTGCTAACAATGTTATCACTCTCTGTT |
| pcDNA3.1-MS2-MARL-mut1-F | CCAAACTACAAACTAGAATGGTCAATGTGTGTTCACA |
| pcDNA3.1-MS2-MARL-mut1-R | TCTAGTTTGTAGTTTGGTACATGACCCCCTCG |
| pcDNA3.1-MS2-MARL-mut2-F | CCGCACTACAATACAGCTTGTGCATTTCTGTTTATCA |
| pcDNA3.1-MS2-MARL-mut2-R | GCTGTATTGTAGTGCGGATGGAAATTCCCTTC |
| pcDNA3.1-MS2-MAVS-3’UTR-F | ACTATAGGGAGACCCAAGCTTGAAGATCAGATTAGGCTTTAAGGCTG |
| pcDNA3.1-MS2-MAVS-3’UTR-R | GCGGCCGTTACTAGTGGATCCGCAGGAATGAAATACAGTATTTATTAGTGT |
| pcDNA3.1-MS2 MAVS-3’UTR-mut-F | CCAAATCTACCACACTGATTGTTAAGTTCTGGGTTGAGAT |
| pcDNA3.1-MS2 MAVS-3’UTR-mut-R | TCAGTGTGGTAGATTTGGTGTCTGCTCGATCGG |
