# Supplementary figures and images for: Long noncoding RNA MARL regulates antiviral responses through suppression miR-122-dependent MAVS downregulation in lower vertebrates
Source: PLoS Pathog. 2020 Jul 17;16(7):e1008670. doi: 10.1371/journal.ppat.1008670 (PMC7390449; doi:10.1371/journal.ppat.1008670)

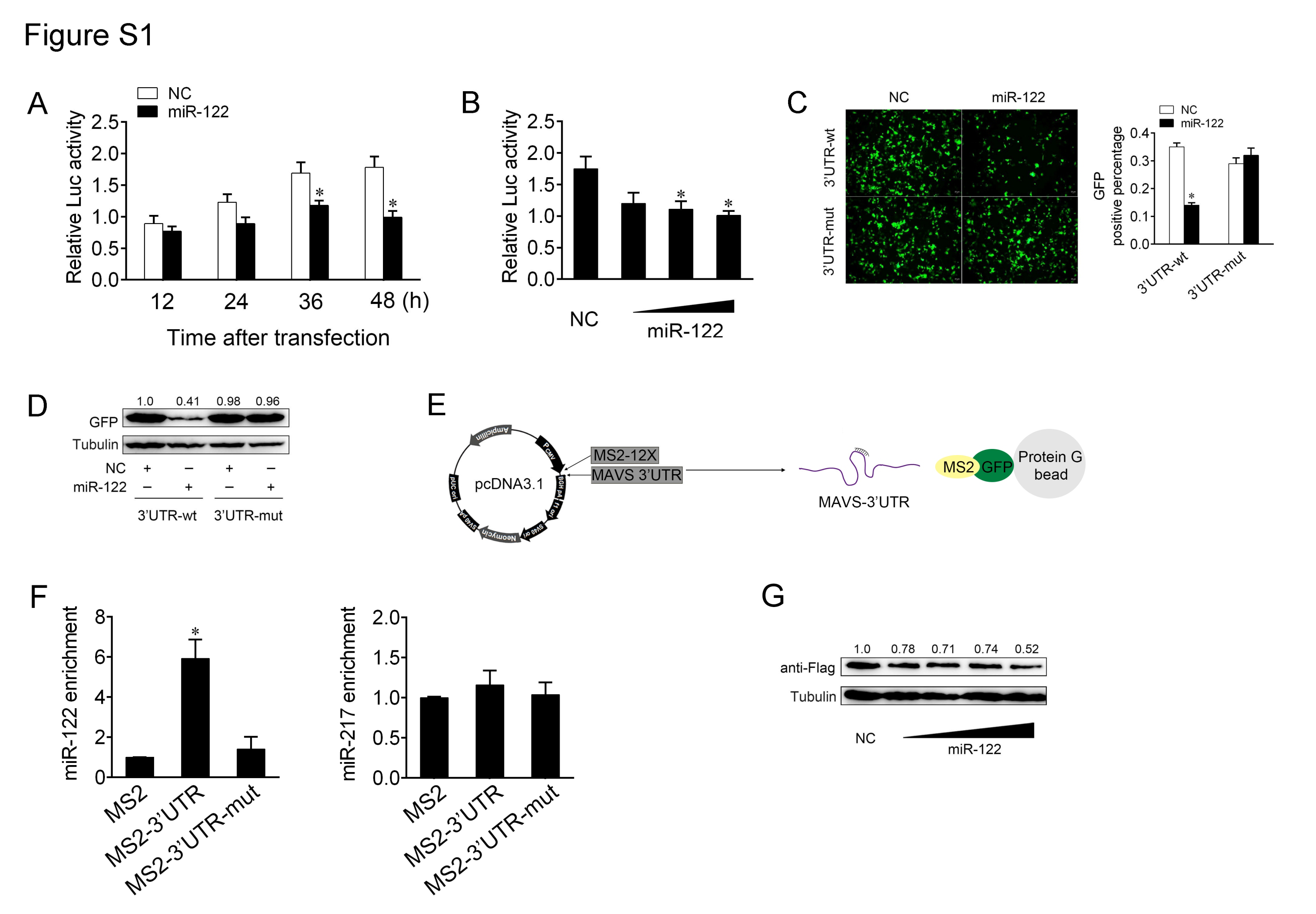

Supplement: S1 Fig — (A) The time gradient experiment was conducted for transfection of miR-122 mimics. Luciferase activity was normalized to renilla luciferase activity. (B) The miR-122 (0, 30, 60, and 90 nM) together with NC (90, 60, 30, and 0 nM) were cotransfected with MAVS-3’UTR-wt into EPC cells. At 48 h post-transfection, the luciferase activity was determined. Luciferase activity was normalized to renilla luciferase activity. (C and D) miR-122 could downregulate GFP expression. EPC cells were cotransfected with the wild type of mVenus-MAVS-3’UTR or the mutated type of mVenus-MAVS-3’UTR, together with NC or miR-122. At 48 h post-transfection, the fluorescence intensity (C) and the GFP expression levels (D) were evaluated by enzyme-labeled instrument and western blotting, respectively. Scale bar, 20 μm; original magnification × 10. (E) The schematic diagram of RIP method to identify the binding between MAVS-3’UTR and miR-122. (F) The qPCR results of the MS2-RIP method used to identify the binding between MAVS-3’UTR and miR-122 in MIC cells. The qPCR for miR-122, as well as negative control miR-217, was performed after RIP process. (G) miR-122 regulate MAVS expression. EPC cells were cotransfected with MAVS expression plasmid, along with miR-122 or NC. At 48 h post-transfection, MAVS expression were determined by western blotting. All data represented the mean ± SE from three independent triplicated experiments. *, p < 0.05. (TIF) [file ppat.1008670.s002.tif]

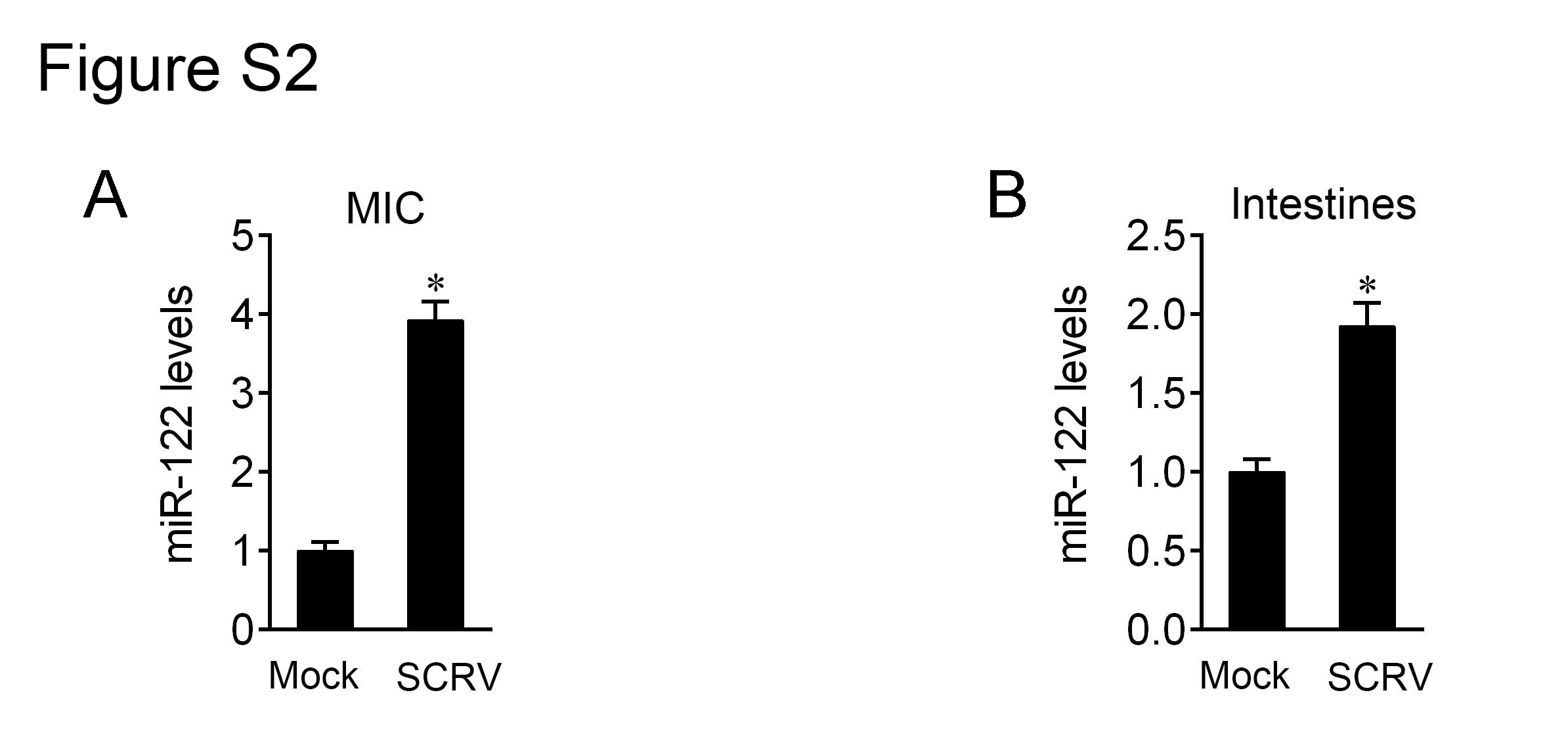

Supplement: S2 Fig — The expression levels of miR-122 in MIC cells (A) and intestine samples (B) were measured by qPCR at 24 h after SCRV infection. All data represented the mean ± SE from three independent triplicated experiments. *, p < 0.05. (TIF) [file ppat.1008670.s003.tif]

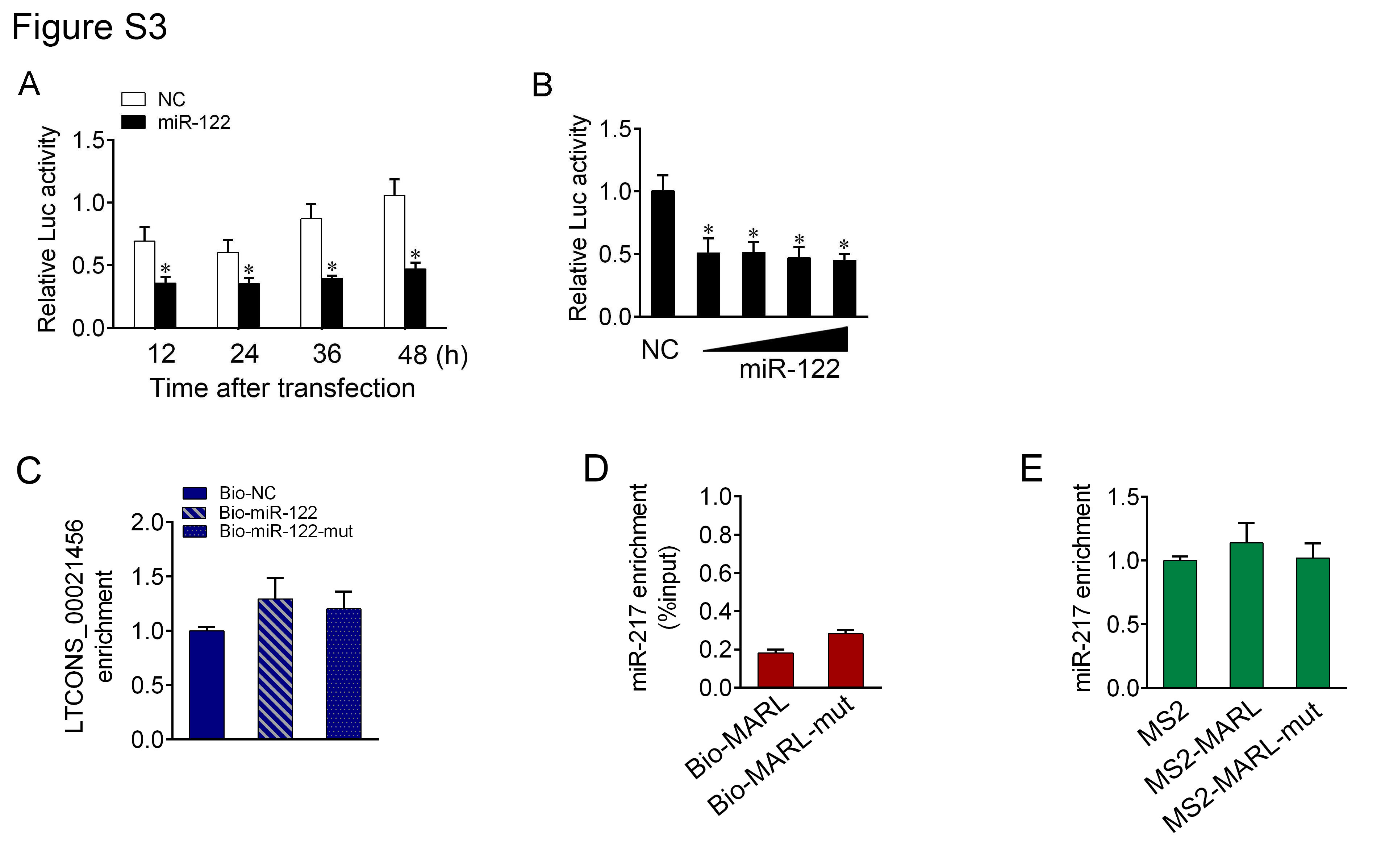

Supplement: S3 Fig — (A) The time gradient experiment was conducted for transfection. (B) The miR-122 (0, 30, 60, and 90 nM) together with NC (90, 60, 30, and 0 nM) were cotransfected with Luc-MARL-wt into EPC cells. At 48 h post-transfection, the luciferase activity was determined. Luciferase activity was normalized to renilla luciferase activity. (C) MIC cells were transfected with the biotinylated wild type of miR-122 (Bio-miR-122-wt) or the biotinylated mutated type of miR-122 (Bio-miR-122-mut) for 48 h. Cells were harvested for biotin-based pulldown assay. The expression of negative control, non-targeted lncRNAs (LTCONS_00021456) was analyzed by qPCR. (D) MIC lysates were incubated with biotin-labeled MARL and MARL-mut. The qPCR for negative control, other non-interacted miRNAs (miR-217) was performed after pull down process. (E) The qPCR results of the MS2-RIP method was conducted to test the expression of negative control, other non-interacted miRNAs (miR-217). All data represented the mean ± SE from three independent triplicated experiments. *, p < 0.05. (TIF) [file ppat.1008670.s004.tif]

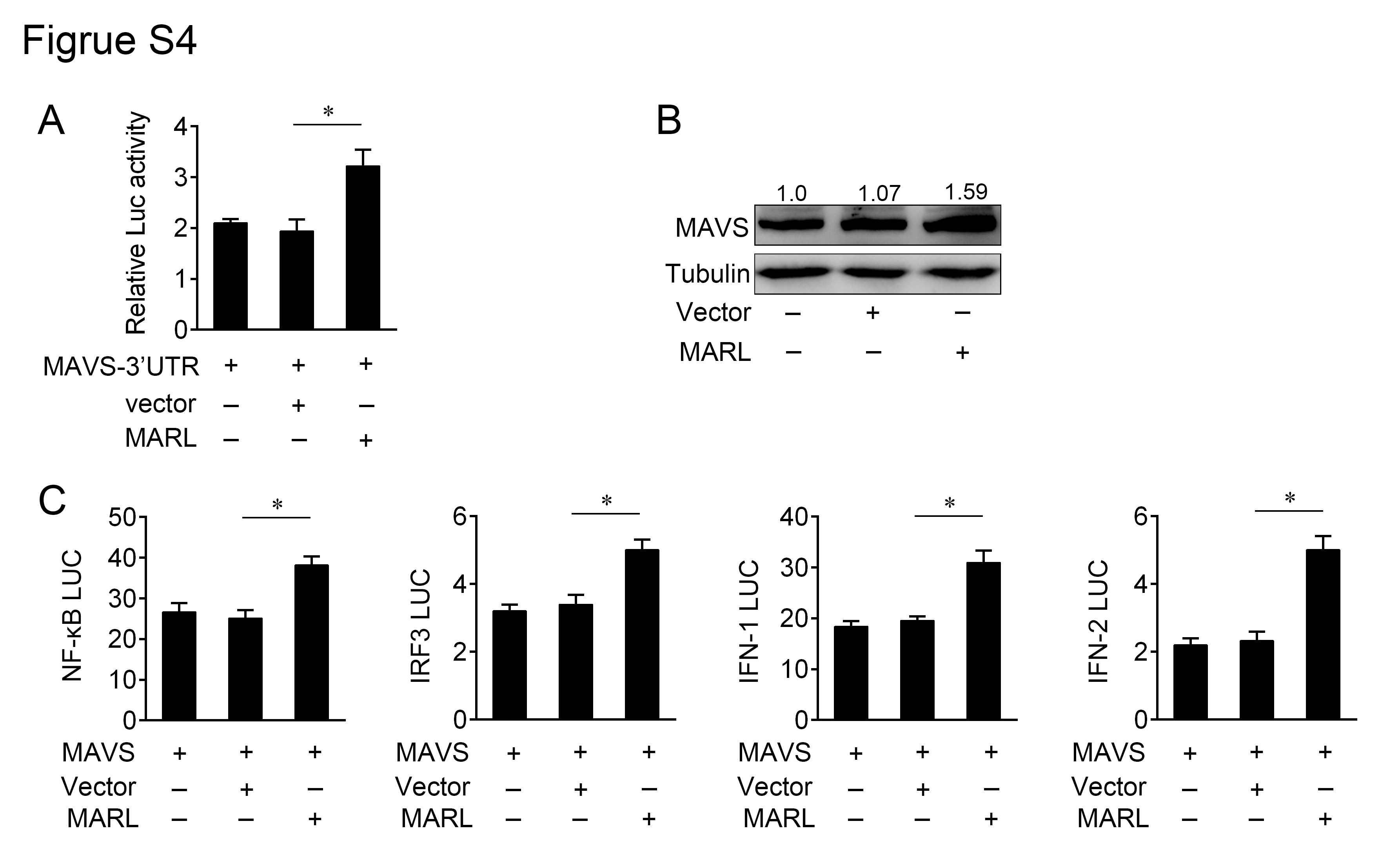

Supplement: S4 Fig — (A) MARL overexpression induced the luciferase activity under the transfection of wild-type MAVS 3’UTR. MIC cells were transfected with pcDNA3.1 vector or MARL expression plasmid, together with MAVS 3’UTR luciferase reporter genes for 48 h. Luciferase activity was analyzed and normalized to renilla luciferase activity. (B) MARL affect the expression of endogenous MAVS. MIC cells were tranfected with pcDNA3.1 vector or MARL expression plasmid for 48 h. MAVS expression were analyzed by western blotting. (C) MARL affects MAVS-mediated signaling. MIC cells were cotransfected with pRL-TK Renilla luciferase plasmid, luciferase reporter genes, pcDNA3.1 vector or MARL expression plasmid, together with MAVS expression plasmid for 48 h. The luciferase activity was measured and normalized to renilla luciferase activity. All data represented the mean ± SE from three independent triplicated experiments. *, p < 0.05. (TIF) [file ppat.1008670.s005.tif]

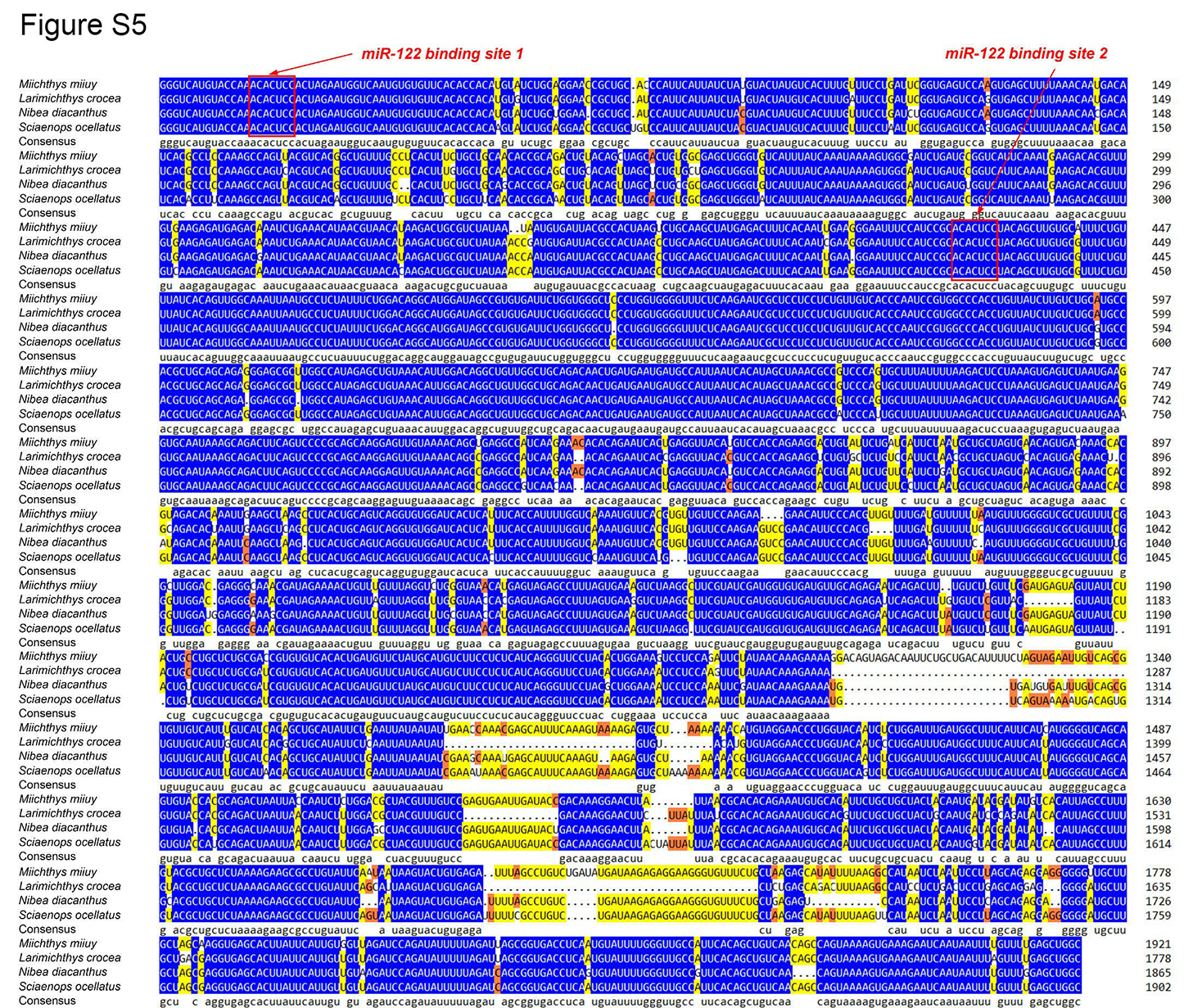

Supplement: S5 Fig — miR-122 binding sites are shown in boxes. (TIF) [file ppat.1008670.s006.tif]
