# Supplementary figures and images for: Correction: Long noncoding RNA MARL regulates antiviral responses through suppression miR-122-dependent MAVS downregulation in lower vertebrates
Source: PLoS Pathog. 2025 Apr 7;21(4):e1013059. doi: 10.1371/journal.ppat.1013059 (PMC11975088; doi:10.1371/journal.ppat.1013059)

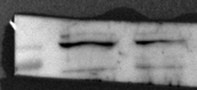

Supplement: S2 File — Underlying image data for Figures 1-9, S1, and S4. (ZIP) [file ppat.1013059.s002.zip › S2 File/Figure1-Detailed raw data/Figure1D/MAVS.tif]

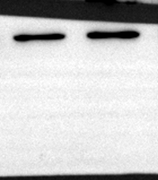

Supplement: S2 File — Underlying image data for Figures 1-9, S1, and S4. (ZIP) [file ppat.1013059.s002.zip › S2 File/Figure1-Detailed raw data/Figure1D/Tubulin.tif]

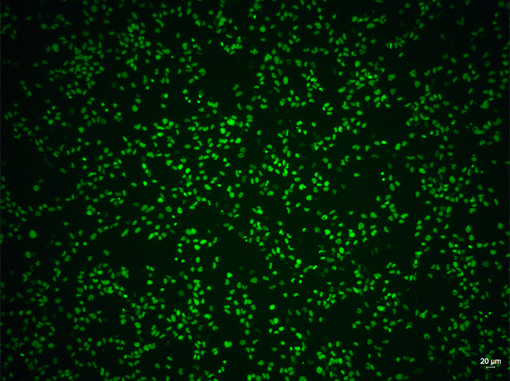

Supplement: S2 File — Underlying image data for Figures 1-9, S1, and S4. (ZIP) [file ppat.1013059.s002.zip › S2 File/Figure1-Detailed raw data/New Figure 1G/si-Ctrl-1.tif]

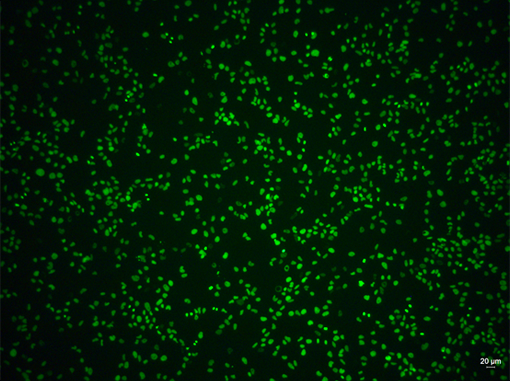

Supplement: S2 File — Underlying image data for Figures 1-9, S1, and S4. (ZIP) [file ppat.1013059.s002.zip › S2 File/Figure1-Detailed raw data/New Figure 1G/si-Ctrl-2.tif]

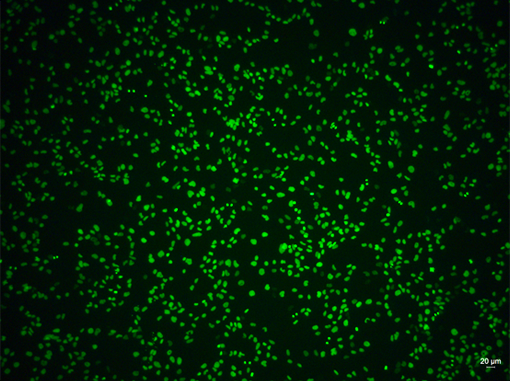

Supplement: S2 File — Underlying image data for Figures 1-9, S1, and S4. (ZIP) [file ppat.1013059.s002.zip › S2 File/Figure1-Detailed raw data/New Figure 1G/si-Ctrl-3.tif]

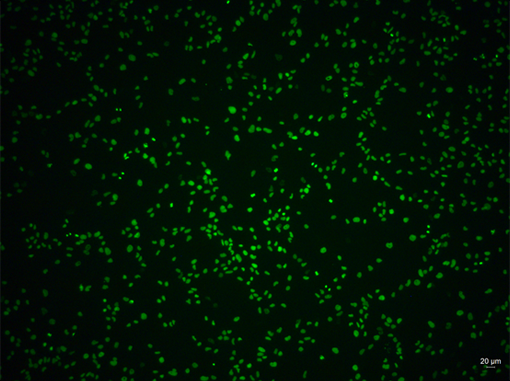

Supplement: S2 File — Underlying image data for Figures 1-9, S1, and S4. (ZIP) [file ppat.1013059.s002.zip › S2 File/Figure1-Detailed raw data/New Figure 1G/si-MAVS-1.tif]

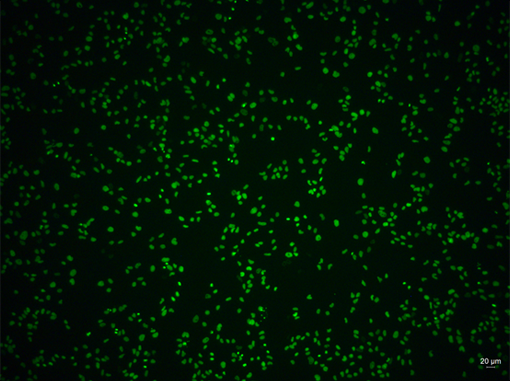

Supplement: S2 File — Underlying image data for Figures 1-9, S1, and S4. (ZIP) [file ppat.1013059.s002.zip › S2 File/Figure1-Detailed raw data/New Figure 1G/si-MAVS-2.tif]

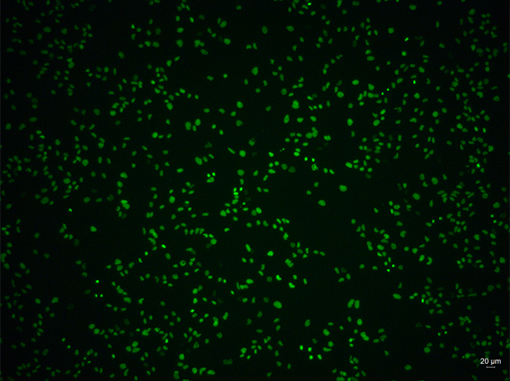

Supplement: S2 File — Underlying image data for Figures 1-9, S1, and S4. (ZIP) [file ppat.1013059.s002.zip › S2 File/Figure1-Detailed raw data/New Figure 1G/si-MAVS-3.tif]

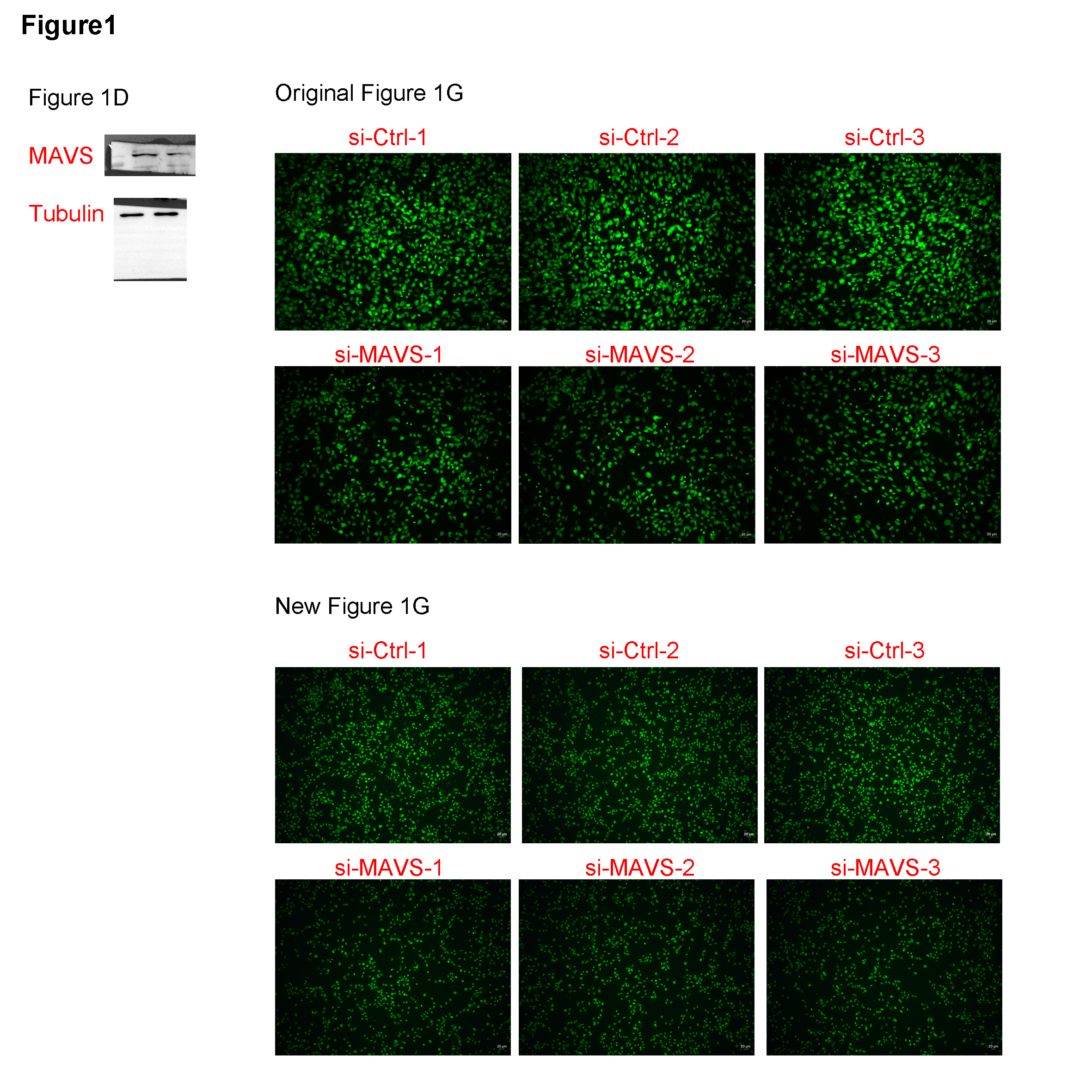

Supplement: S2 File — Underlying image data for Figures 1-9, S1, and S4. (ZIP) [file ppat.1013059.s002.zip › S2 File/Figure1.tif]

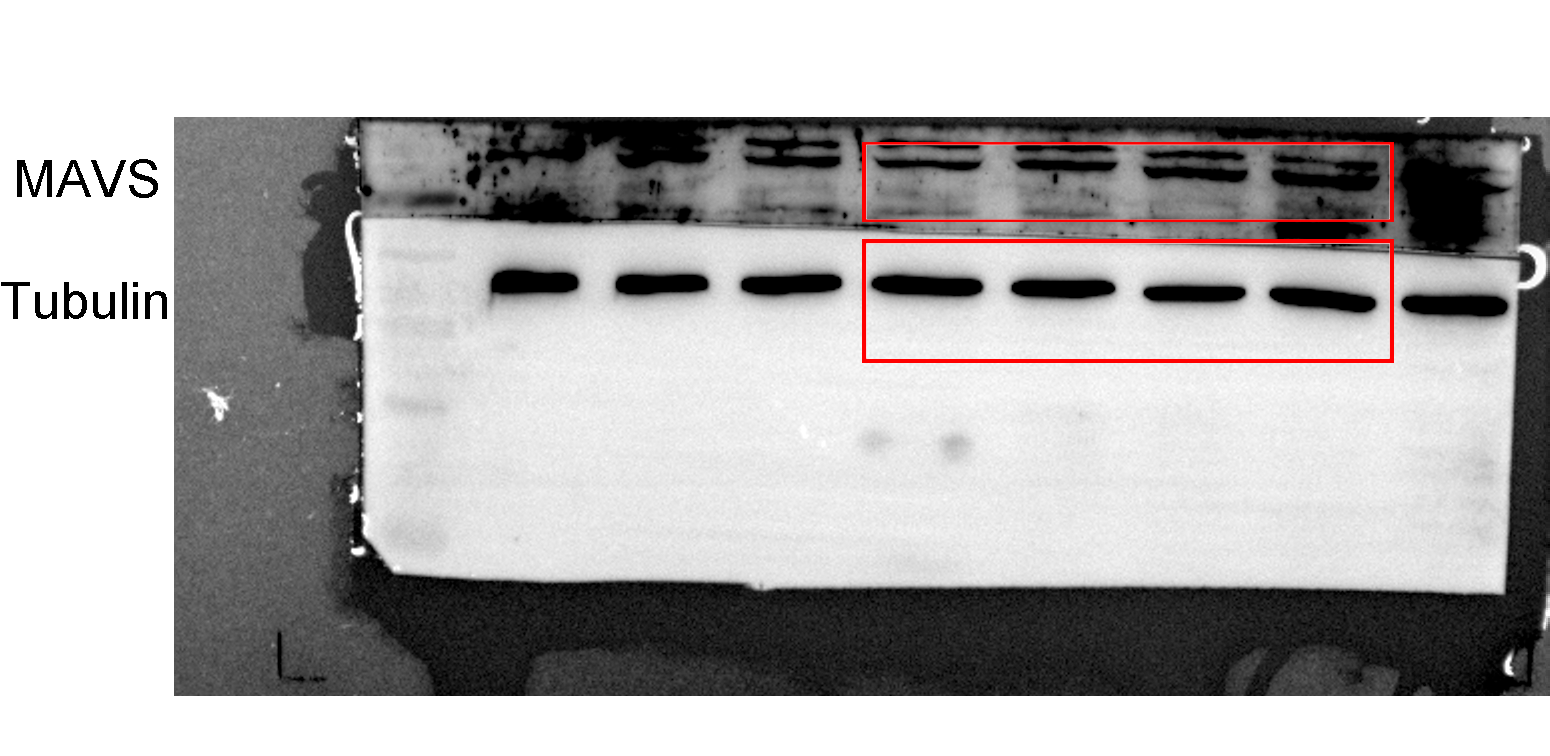

Supplement: S2 File — Underlying image data for Figures 1-9, S1, and S4. (ZIP) [file ppat.1013059.s002.zip › S2 File/Figure2-Detailed raw data/Figure2C/Figure2C-miR-122-i.tif]

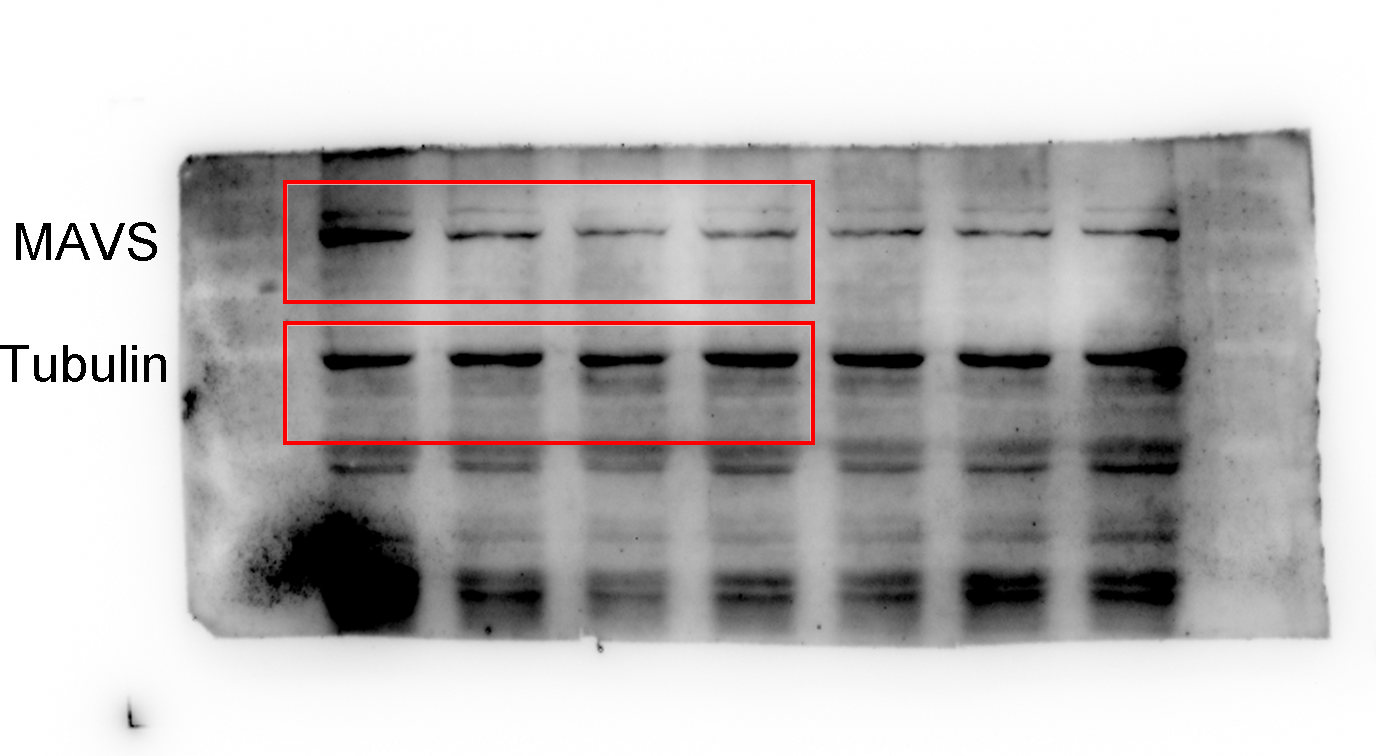

Supplement: S2 File — Underlying image data for Figures 1-9, S1, and S4. (ZIP) [file ppat.1013059.s002.zip › S2 File/Figure2-Detailed raw data/Figure2C/Figure2C-miR-122.tif]

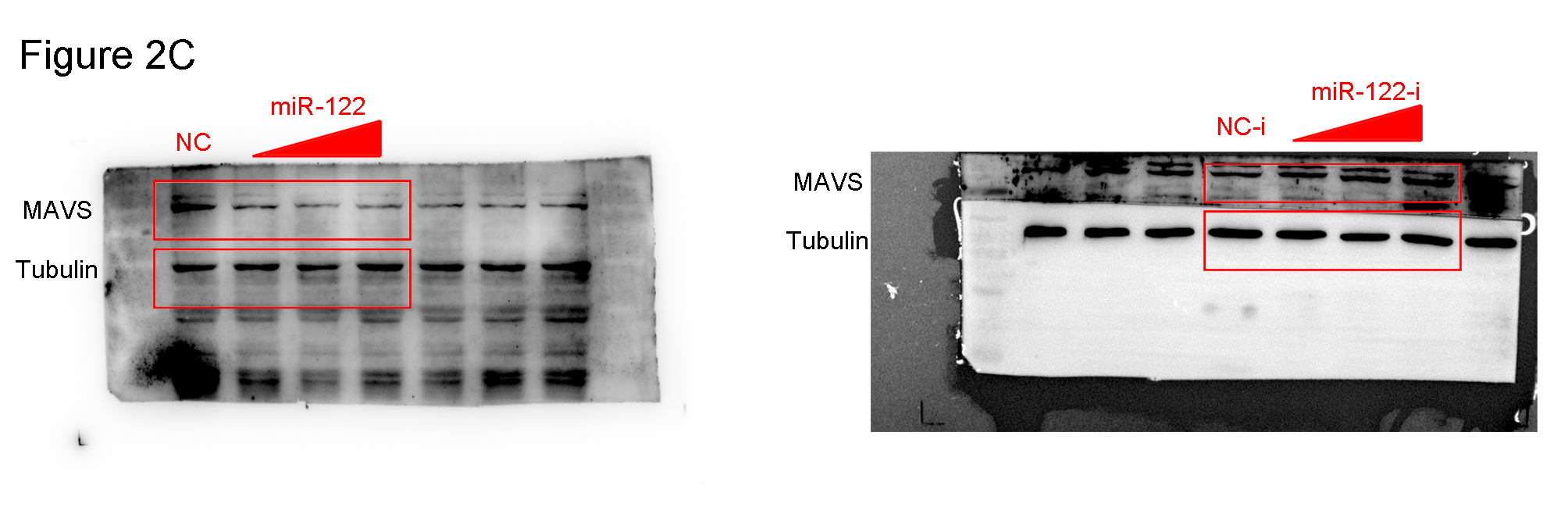

Supplement: S2 File — Underlying image data for Figures 1-9, S1, and S4. (ZIP) [file ppat.1013059.s002.zip › S2 File/Figure2.tif]

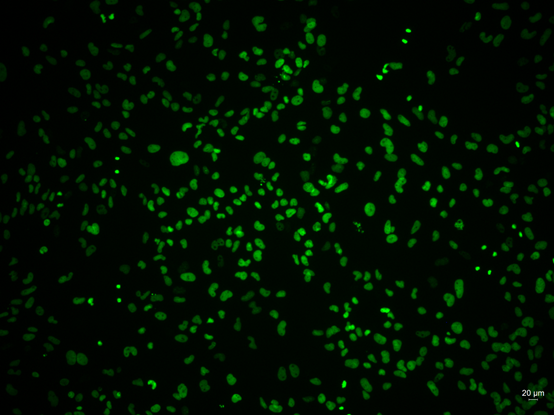

Supplement: S2 File — Underlying image data for Figures 1-9, S1, and S4. (ZIP) [file ppat.1013059.s002.zip › S2 File/Figure3/Figure 3G/miR-122-1.tif]

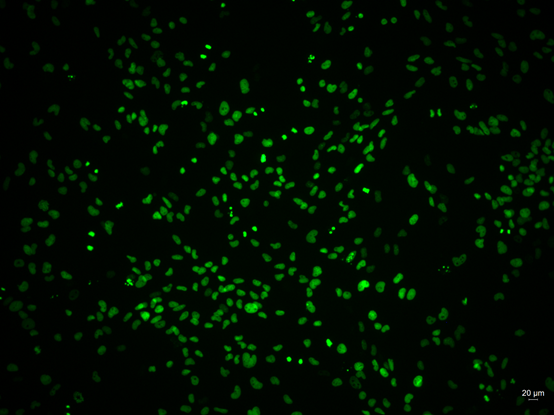

Supplement: S2 File — Underlying image data for Figures 1-9, S1, and S4. (ZIP) [file ppat.1013059.s002.zip › S2 File/Figure3/Figure 3G/miR-122-2.tif]

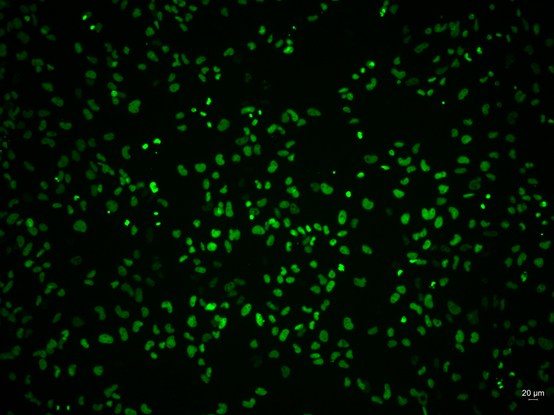

Supplement: S2 File — Underlying image data for Figures 1-9, S1, and S4. (ZIP) [file ppat.1013059.s002.zip › S2 File/Figure3/Figure 3G/miR-122-3.tif]

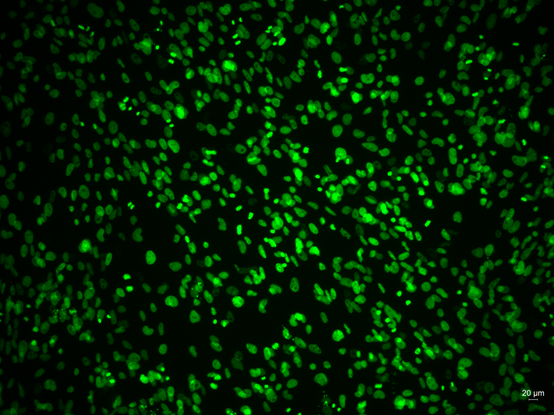

Supplement: S2 File — Underlying image data for Figures 1-9, S1, and S4. (ZIP) [file ppat.1013059.s002.zip › S2 File/Figure3/Figure 3G/NC-1.tif]

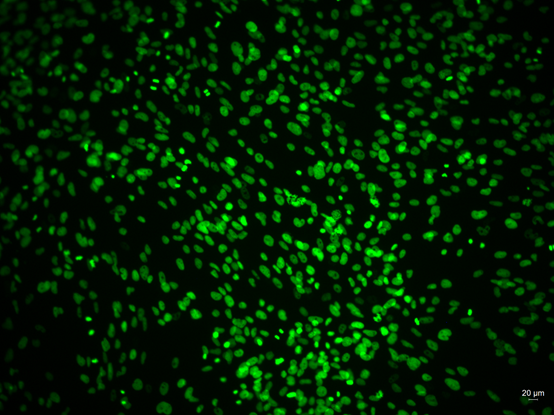

Supplement: S2 File — Underlying image data for Figures 1-9, S1, and S4. (ZIP) [file ppat.1013059.s002.zip › S2 File/Figure3/Figure 3G/NC-2.tif]

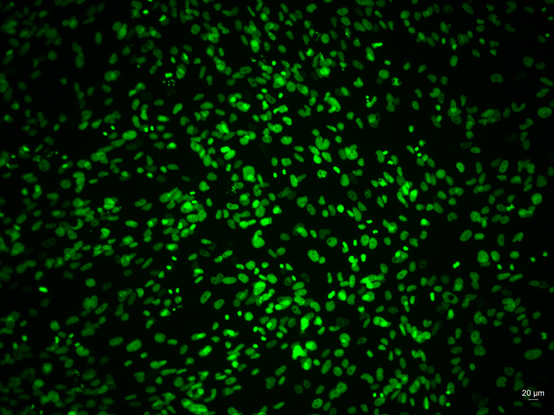

Supplement: S2 File — Underlying image data for Figures 1-9, S1, and S4. (ZIP) [file ppat.1013059.s002.zip › S2 File/Figure3/Figure 3G/NC-3.tif]

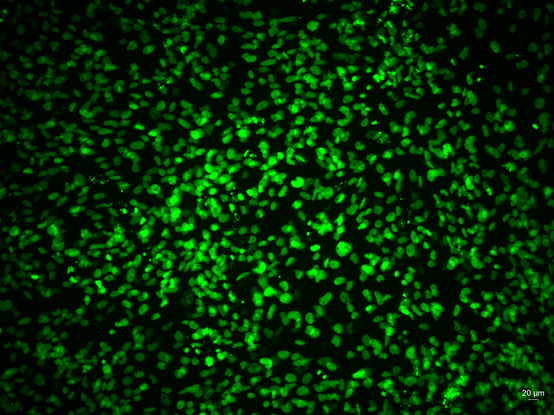

Supplement: S2 File — Underlying image data for Figures 1-9, S1, and S4. (ZIP) [file ppat.1013059.s002.zip › S2 File/Figure3/Figure 3H/miR-122-i-1.tif]

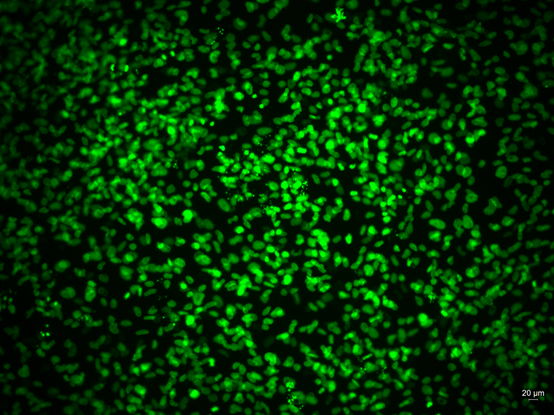

Supplement: S2 File — Underlying image data for Figures 1-9, S1, and S4. (ZIP) [file ppat.1013059.s002.zip › S2 File/Figure3/Figure 3H/miR-122-i-2.tif]

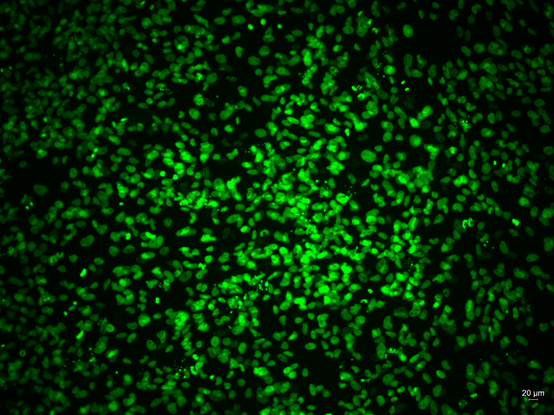

Supplement: S2 File — Underlying image data for Figures 1-9, S1, and S4. (ZIP) [file ppat.1013059.s002.zip › S2 File/Figure3/Figure 3H/miR-122-i-3.tif]

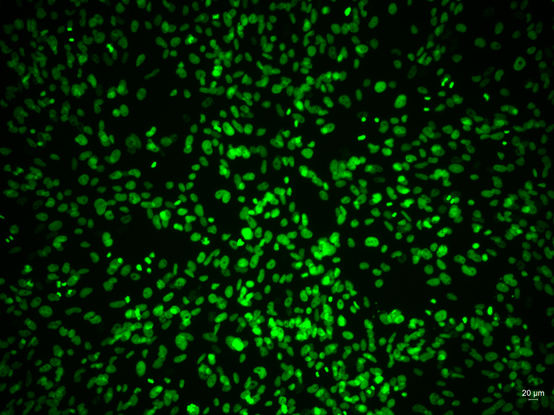

Supplement: S2 File — Underlying image data for Figures 1-9, S1, and S4. (ZIP) [file ppat.1013059.s002.zip › S2 File/Figure3/Figure 3H/NC-i-1.tif]

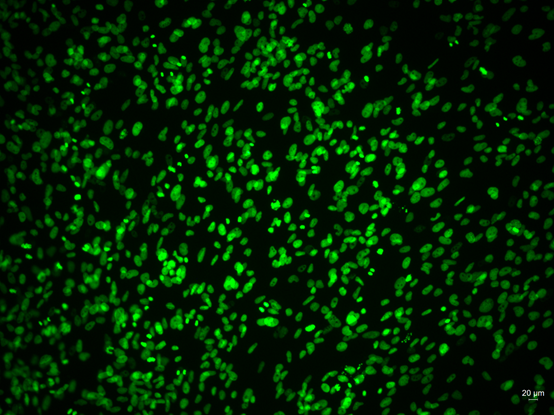

Supplement: S2 File — Underlying image data for Figures 1-9, S1, and S4. (ZIP) [file ppat.1013059.s002.zip › S2 File/Figure3/Figure 3H/NC-i-2.tif]

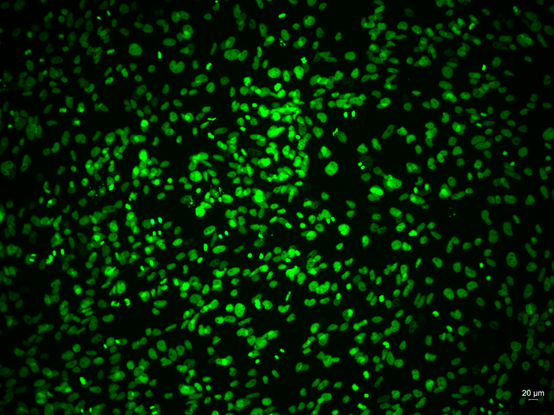

Supplement: S2 File — Underlying image data for Figures 1-9, S1, and S4. (ZIP) [file ppat.1013059.s002.zip › S2 File/Figure3/Figure 3H/NC-i-3.tif]

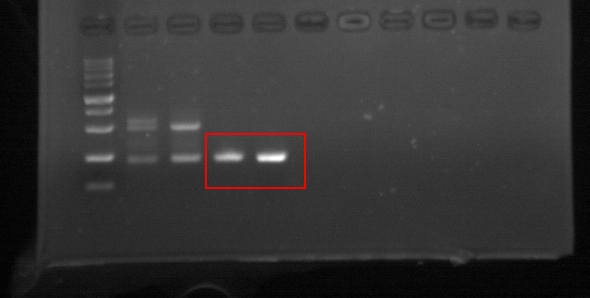

Supplement: S2 File — Underlying image data for Figures 1-9, S1, and S4. (ZIP) [file ppat.1013059.s002.zip › S2 File/Figure4-Detailed raw data/Figure4F/Figure4F-MARL.tif]

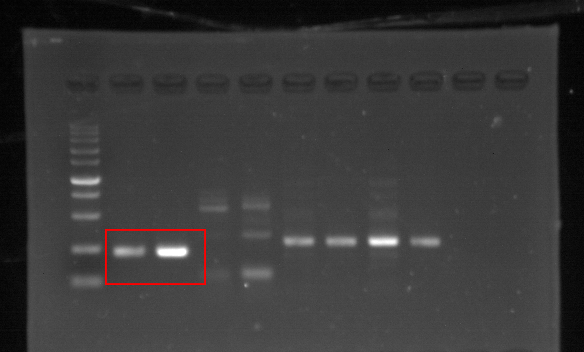

Supplement: S2 File — Underlying image data for Figures 1-9, S1, and S4. (ZIP) [file ppat.1013059.s002.zip › S2 File/Figure4-Detailed raw data/Figure4F/Figure4F-Tubulin.tif]

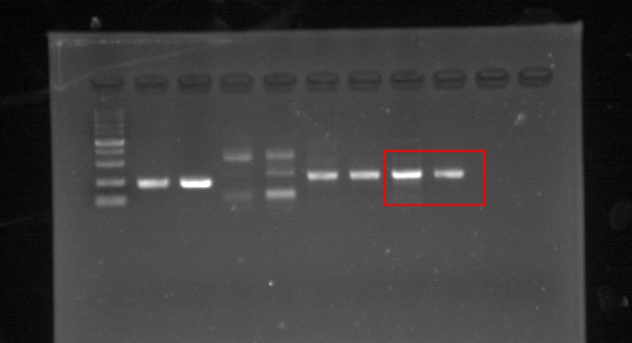

Supplement: S2 File — Underlying image data for Figures 1-9, S1, and S4. (ZIP) [file ppat.1013059.s002.zip › S2 File/Figure4-Detailed raw data/Figure4F/Figure4F-U6.tif]

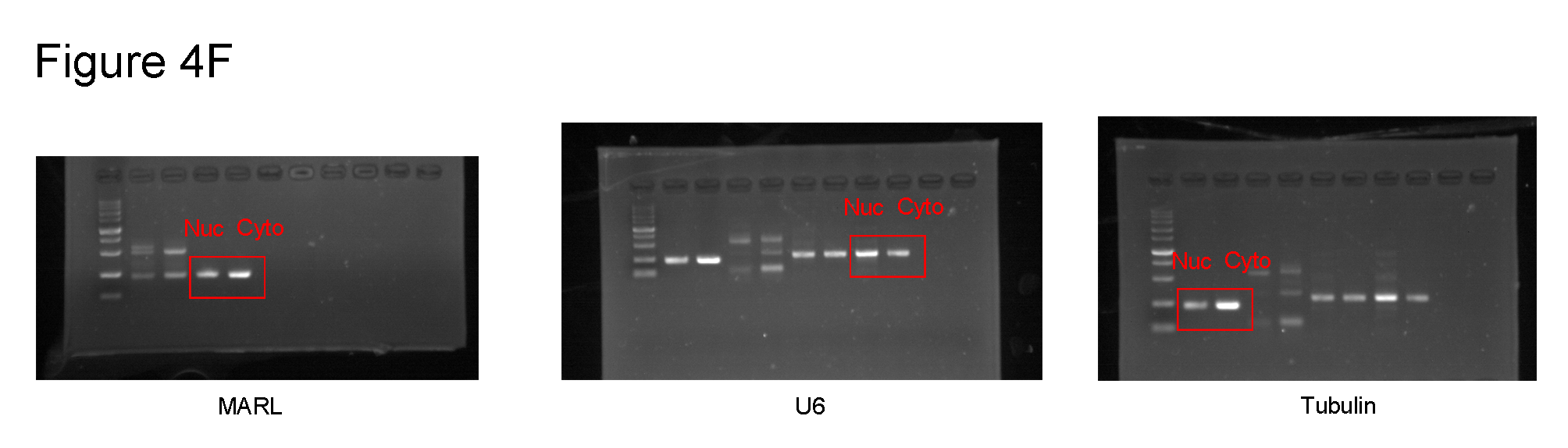

Supplement: S2 File — Underlying image data for Figures 1-9, S1, and S4. (ZIP) [file ppat.1013059.s002.zip › S2 File/Figure4.tif]

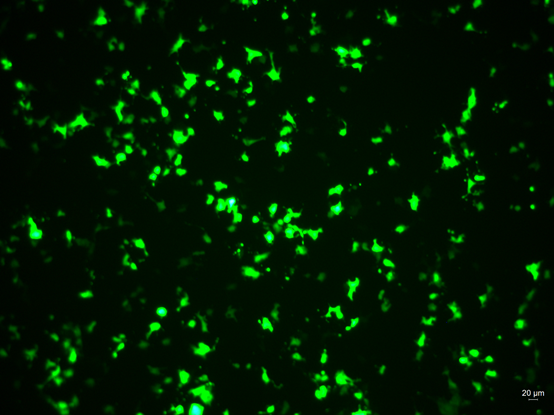

Supplement: S2 File — Underlying image data for Figures 1-9, S1, and S4. (ZIP) [file ppat.1013059.s002.zip › S2 File/Figure6-Detailed raw data/Figure6C/Figure6C-MARL-mut-miR-122.tif]

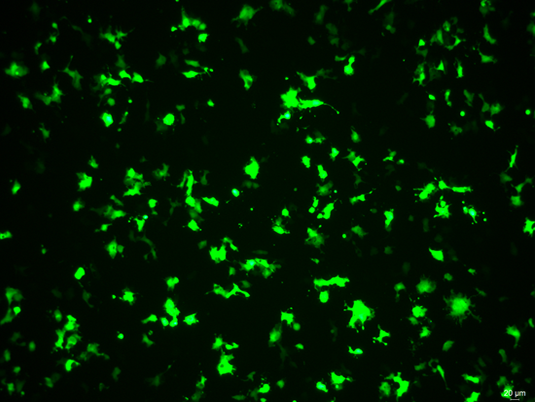

Supplement: S2 File — Underlying image data for Figures 1-9, S1, and S4. (ZIP) [file ppat.1013059.s002.zip › S2 File/Figure6-Detailed raw data/Figure6C/Figure6C-MARL-mut-NC.tif]

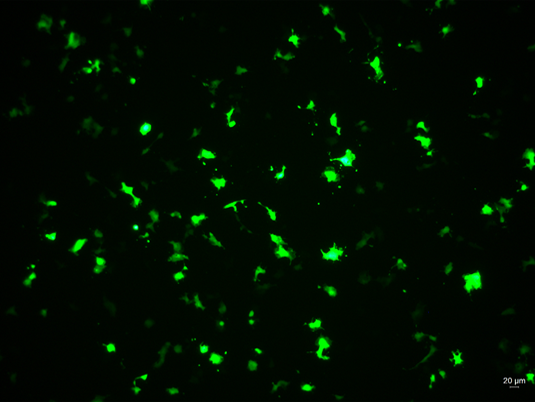

Supplement: S2 File — Underlying image data for Figures 1-9, S1, and S4. (ZIP) [file ppat.1013059.s002.zip › S2 File/Figure6-Detailed raw data/Figure6C/Figure6C-MARL-WT-miR-122.tif]

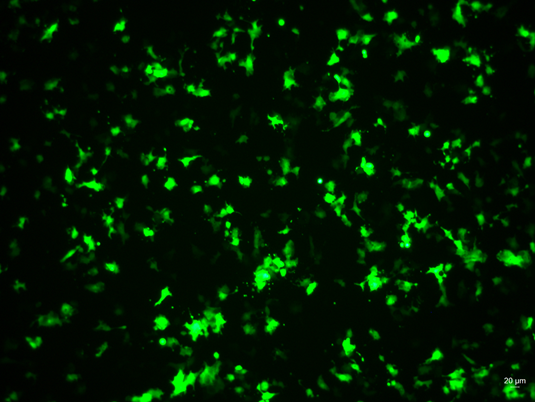

Supplement: S2 File — Underlying image data for Figures 1-9, S1, and S4. (ZIP) [file ppat.1013059.s002.zip › S2 File/Figure6-Detailed raw data/Figure6C/Figure6C-MARL-WT-NC.tif]

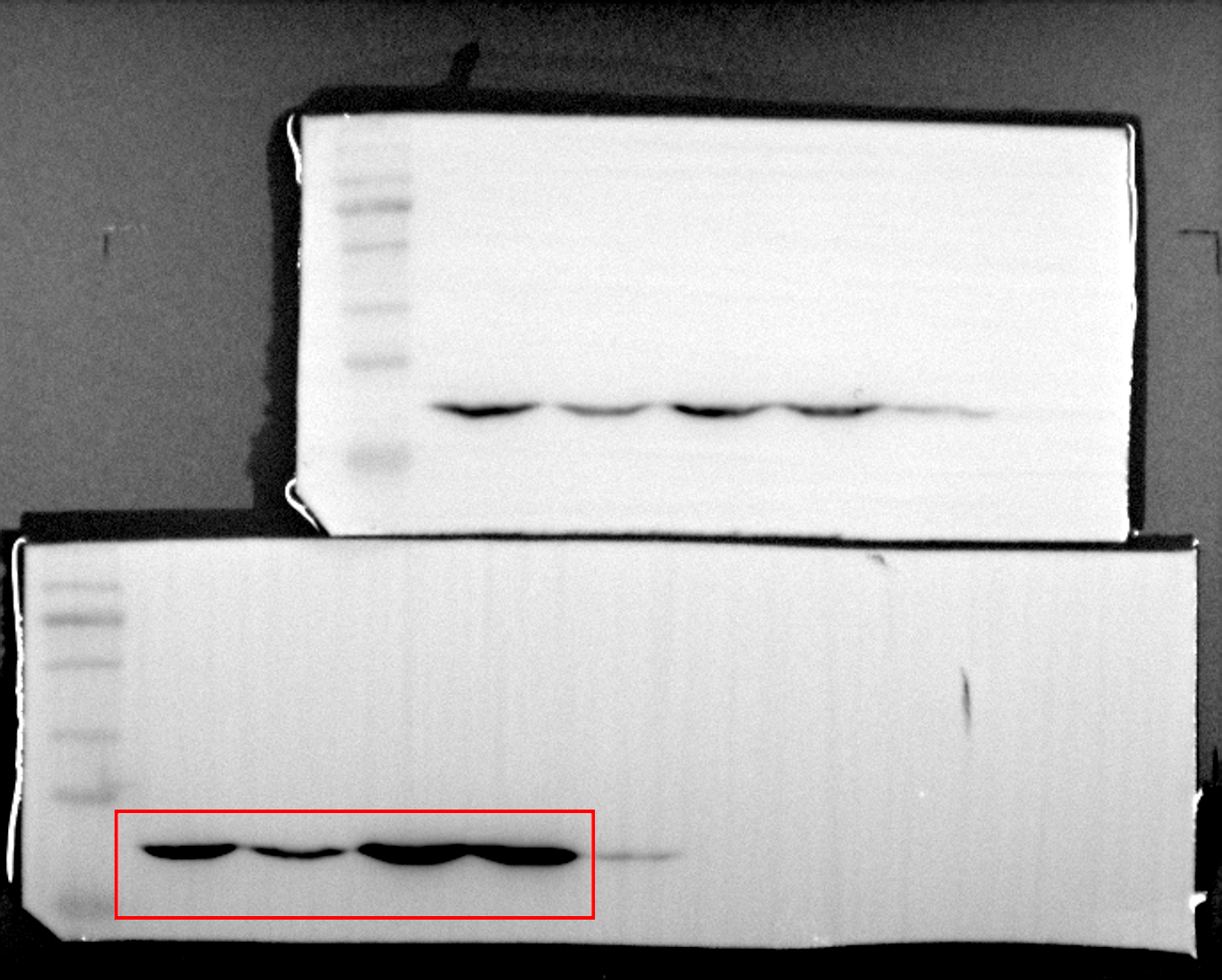

Supplement: S2 File — Underlying image data for Figures 1-9, S1, and S4. (ZIP) [file ppat.1013059.s002.zip › S2 File/Figure6-Detailed raw data/Figure6D/Figure6D-GFP.tif]

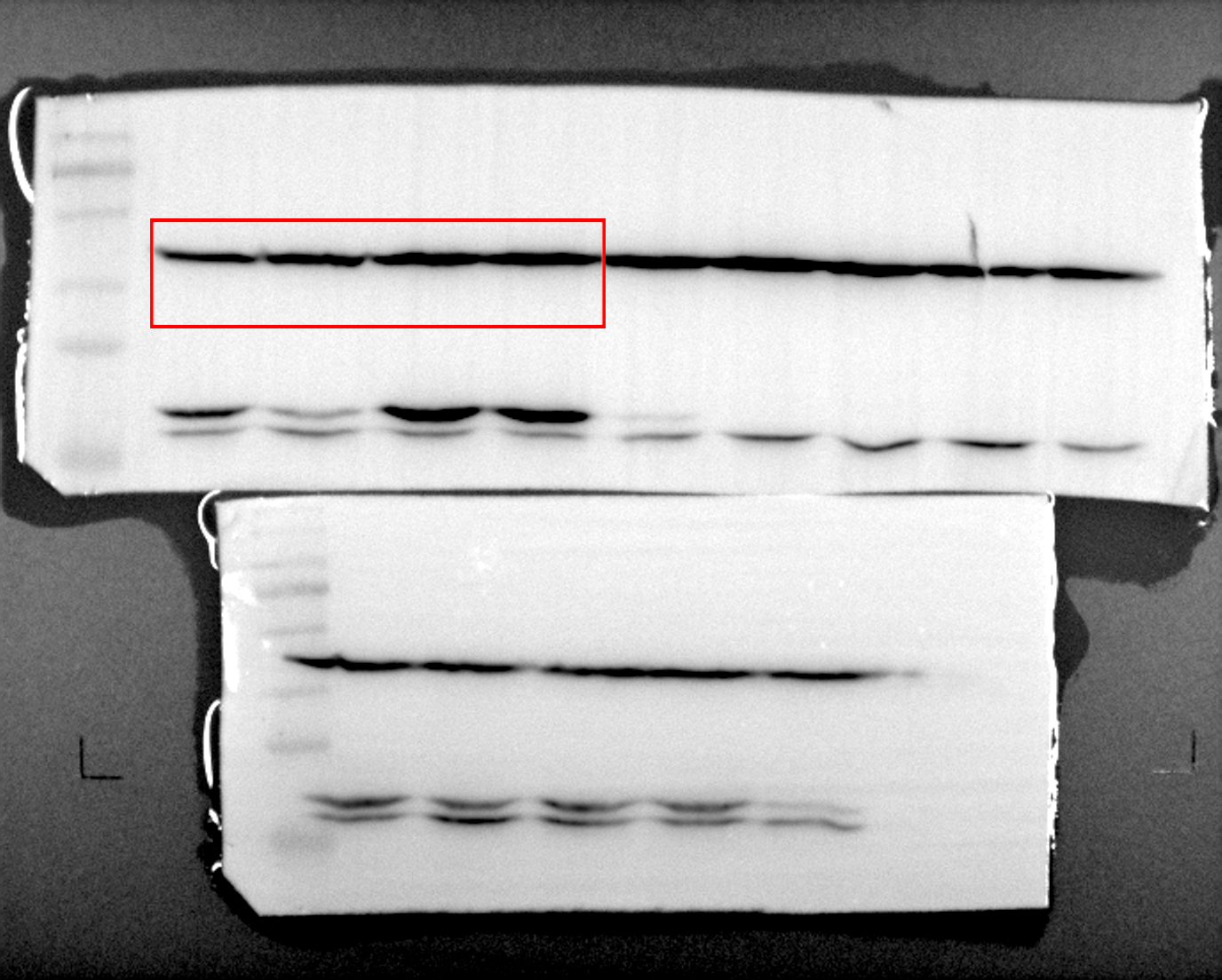

Supplement: S2 File — Underlying image data for Figures 1-9, S1, and S4. (ZIP) [file ppat.1013059.s002.zip › S2 File/Figure6-Detailed raw data/Figure6D/Figure6D-Tubulin.tif]

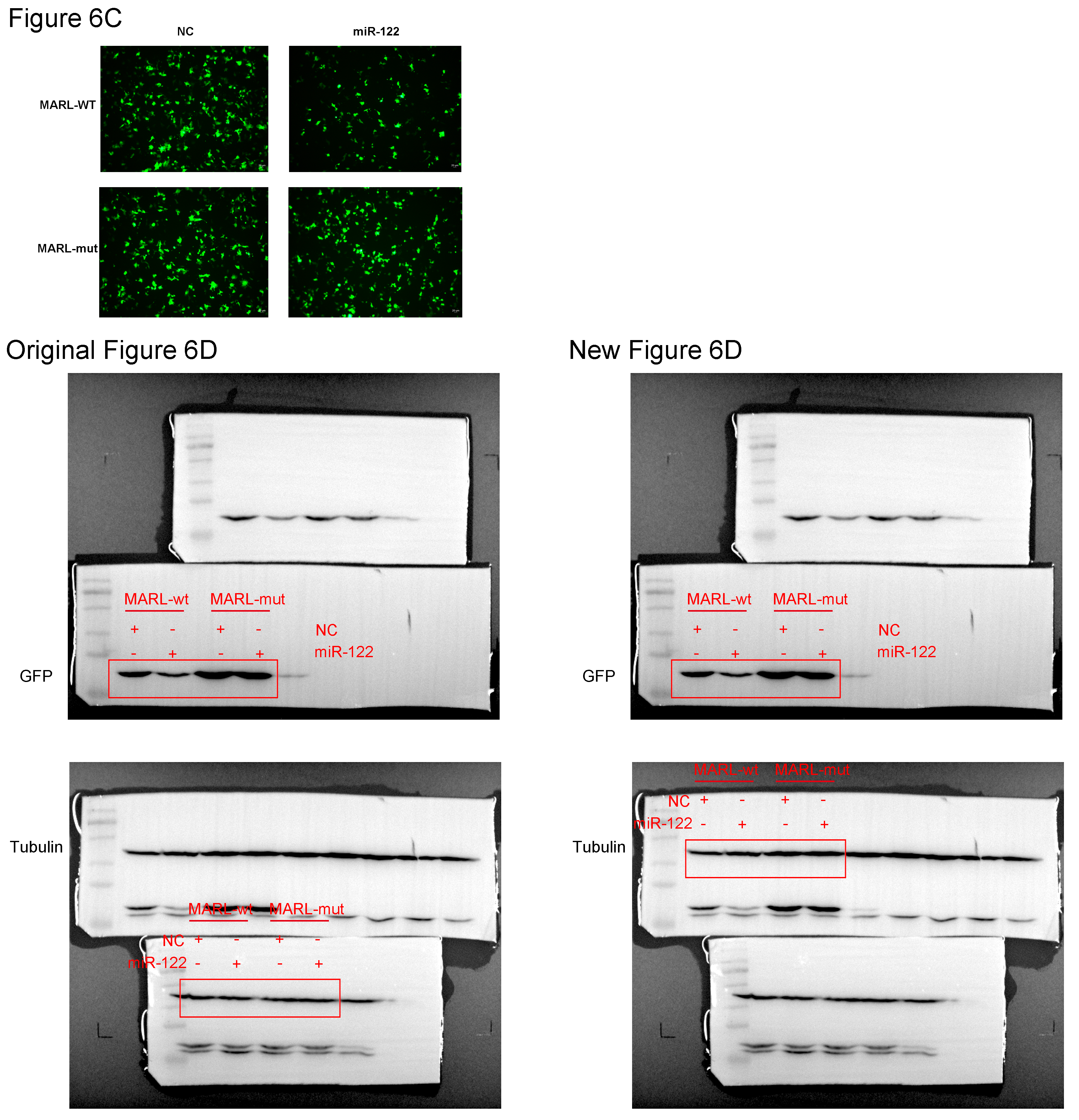

Supplement: S2 File — Underlying image data for Figures 1-9, S1, and S4. (ZIP) [file ppat.1013059.s002.zip › S2 File/Figure6.tif]

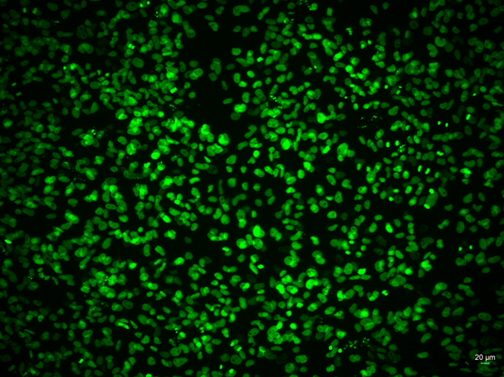

Supplement: S2 File — Underlying image data for Figures 1-9, S1, and S4. (ZIP) [file ppat.1013059.s002.zip › S2 File/Figure7-Detailed raw data/Figure7E/MARL-1.tif]

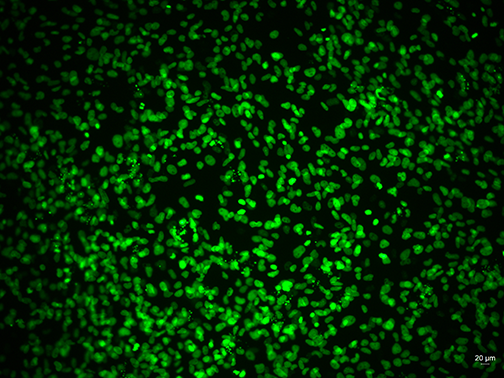

Supplement: S2 File — Underlying image data for Figures 1-9, S1, and S4. (ZIP) [file ppat.1013059.s002.zip › S2 File/Figure7-Detailed raw data/Figure7E/MARL-2.tif]

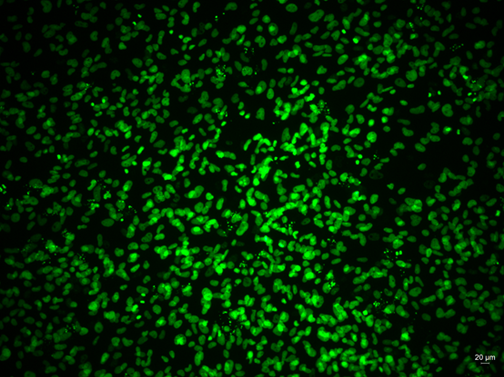

Supplement: S2 File — Underlying image data for Figures 1-9, S1, and S4. (ZIP) [file ppat.1013059.s002.zip › S2 File/Figure7-Detailed raw data/Figure7E/MARL-3.tif]

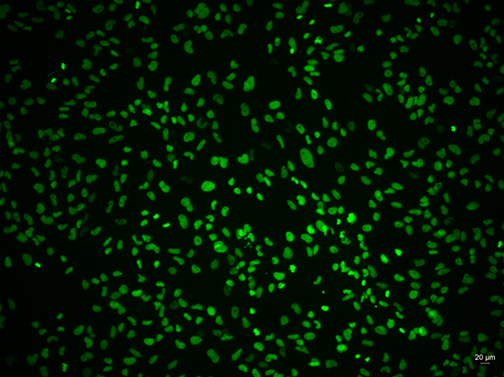

Supplement: S2 File — Underlying image data for Figures 1-9, S1, and S4. (ZIP) [file ppat.1013059.s002.zip › S2 File/Figure7-Detailed raw data/Figure7E/vector-1.tif]

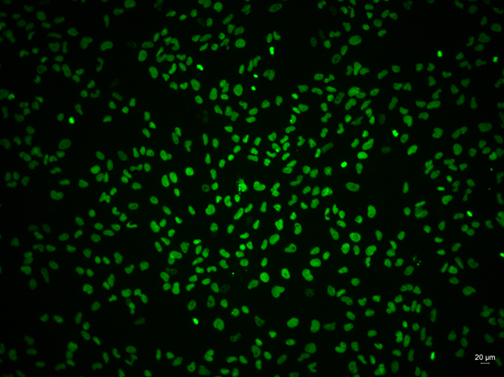

Supplement: S2 File — Underlying image data for Figures 1-9, S1, and S4. (ZIP) [file ppat.1013059.s002.zip › S2 File/Figure7-Detailed raw data/Figure7E/vector-2.tif]

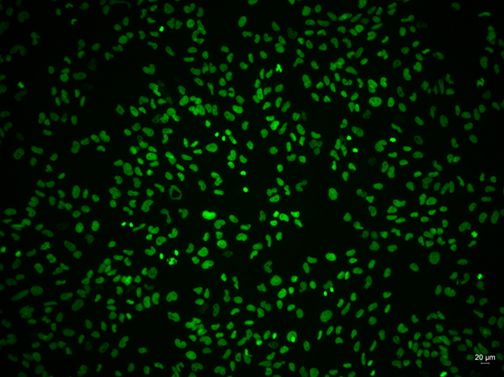

Supplement: S2 File — Underlying image data for Figures 1-9, S1, and S4. (ZIP) [file ppat.1013059.s002.zip › S2 File/Figure7-Detailed raw data/Figure7E/vector-3.tif]

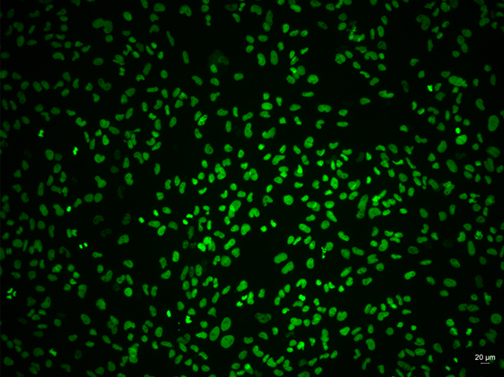

Supplement: S2 File — Underlying image data for Figures 1-9, S1, and S4. (ZIP) [file ppat.1013059.s002.zip › S2 File/Figure7-Detailed raw data/Figure7F/si-Ctrl-1.tif]

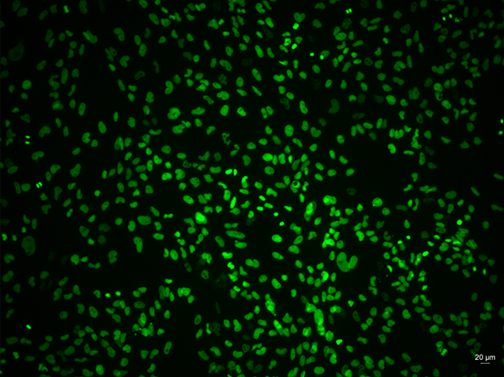

Supplement: S2 File — Underlying image data for Figures 1-9, S1, and S4. (ZIP) [file ppat.1013059.s002.zip › S2 File/Figure7-Detailed raw data/Figure7F/si-Ctrl-2.tif]

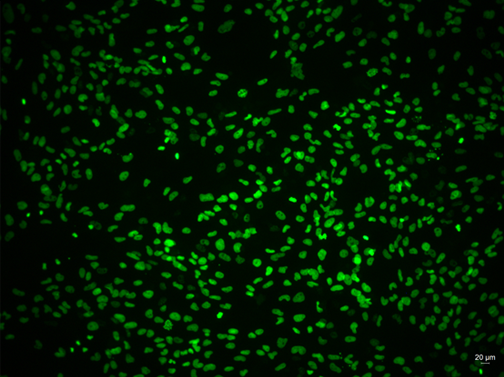

Supplement: S2 File — Underlying image data for Figures 1-9, S1, and S4. (ZIP) [file ppat.1013059.s002.zip › S2 File/Figure7-Detailed raw data/Figure7F/si-Ctrl-3.tif]

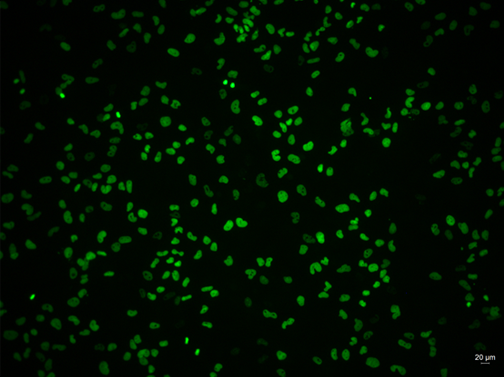

Supplement: S2 File — Underlying image data for Figures 1-9, S1, and S4. (ZIP) [file ppat.1013059.s002.zip › S2 File/Figure7-Detailed raw data/Figure7F/si-MARL-1.tif]

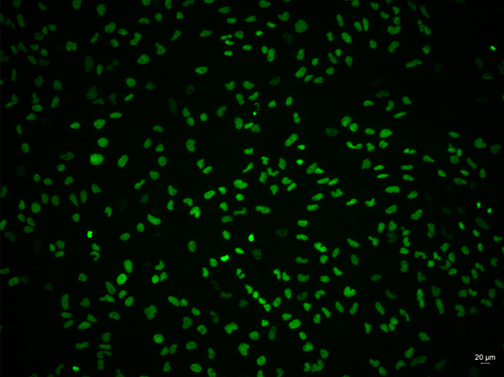

Supplement: S2 File — Underlying image data for Figures 1-9, S1, and S4. (ZIP) [file ppat.1013059.s002.zip › S2 File/Figure7-Detailed raw data/Figure7F/si-MARL-2.tif]

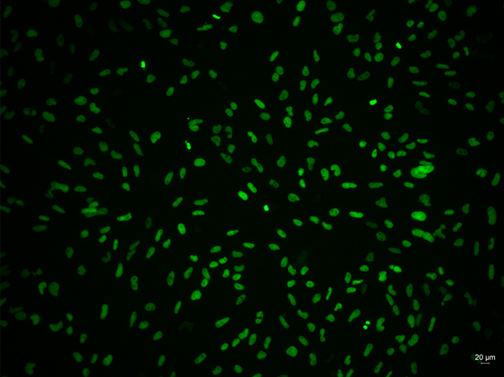

Supplement: S2 File — Underlying image data for Figures 1-9, S1, and S4. (ZIP) [file ppat.1013059.s002.zip › S2 File/Figure7-Detailed raw data/Figure7F/si-MARL-3.tif]

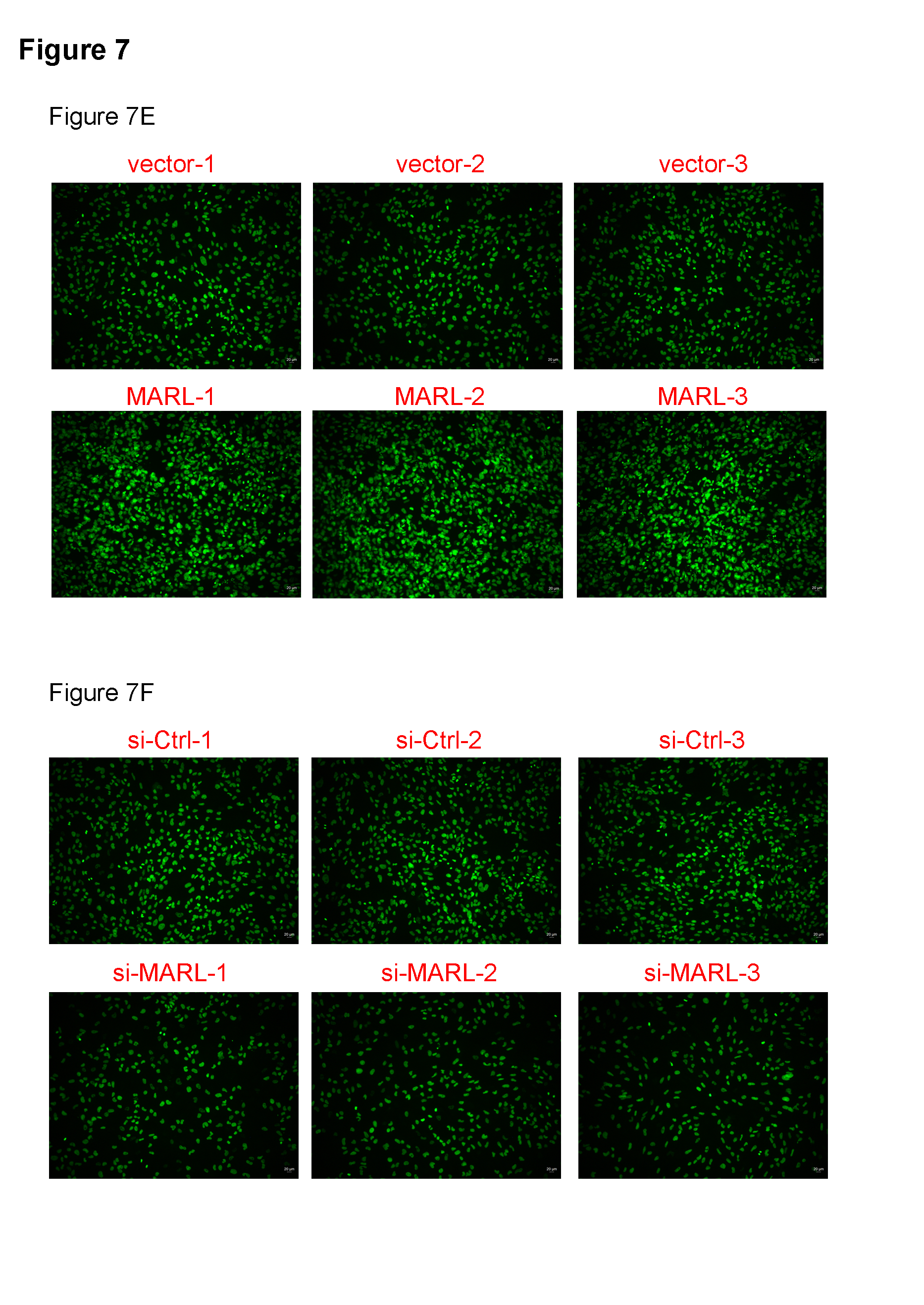

Supplement: S2 File — Underlying image data for Figures 1-9, S1, and S4. (ZIP) [file ppat.1013059.s002.zip › S2 File/Figure7.tif]

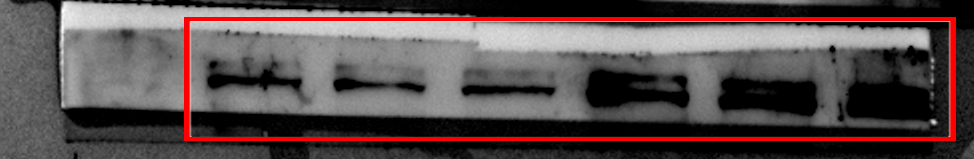

Supplement: S2 File — Underlying image data for Figures 1-9, S1, and S4. (ZIP) [file ppat.1013059.s002.zip › S2 File/Figure8-Detailed raw data/Figure8A/Figure8A-MAVS.tif]

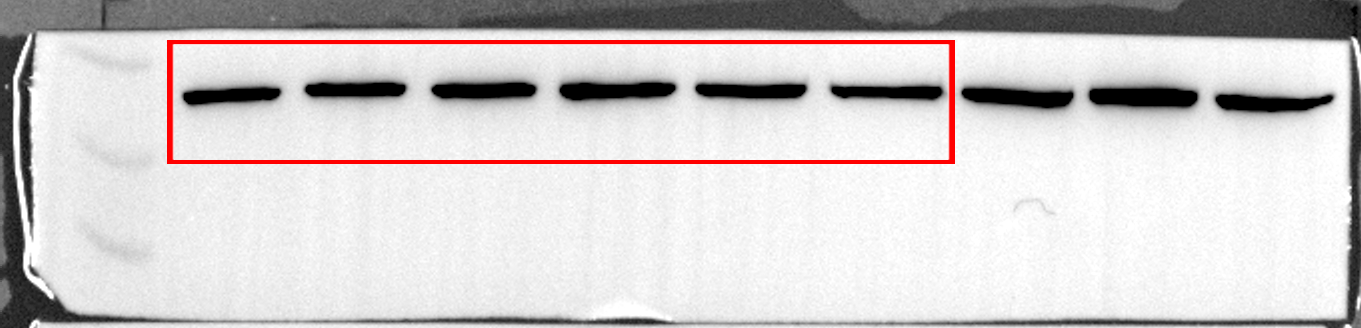

Supplement: S2 File — Underlying image data for Figures 1-9, S1, and S4. (ZIP) [file ppat.1013059.s002.zip › S2 File/Figure8-Detailed raw data/Figure8A/Figure8A-Tubulin.tif]

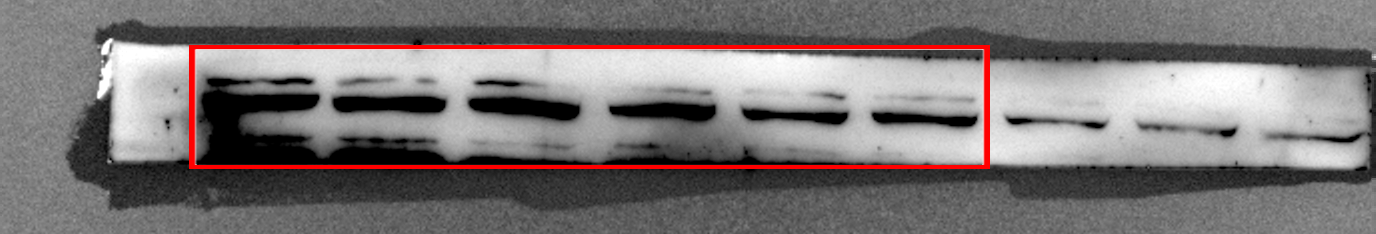

Supplement: S2 File — Underlying image data for Figures 1-9, S1, and S4. (ZIP) [file ppat.1013059.s002.zip › S2 File/Figure8-Detailed raw data/Figure8B/Figure8B-MAVS.tif]

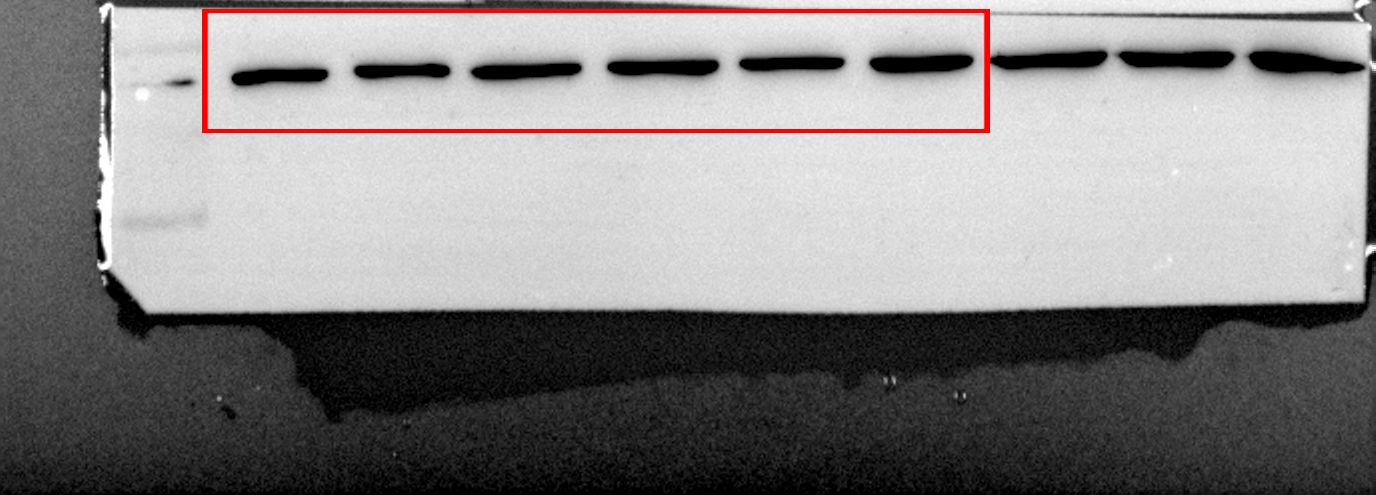

Supplement: S2 File — Underlying image data for Figures 1-9, S1, and S4. (ZIP) [file ppat.1013059.s002.zip › S2 File/Figure8-Detailed raw data/Figure8B/Figure8B-Tubulin.tif]

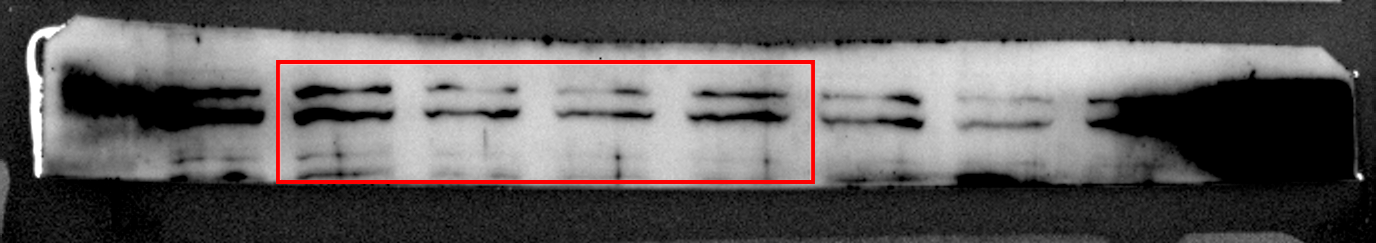

Supplement: S2 File — Underlying image data for Figures 1-9, S1, and S4. (ZIP) [file ppat.1013059.s002.zip › S2 File/Figure8-Detailed raw data/Figure8E/Figure8E-MAVS.tif]

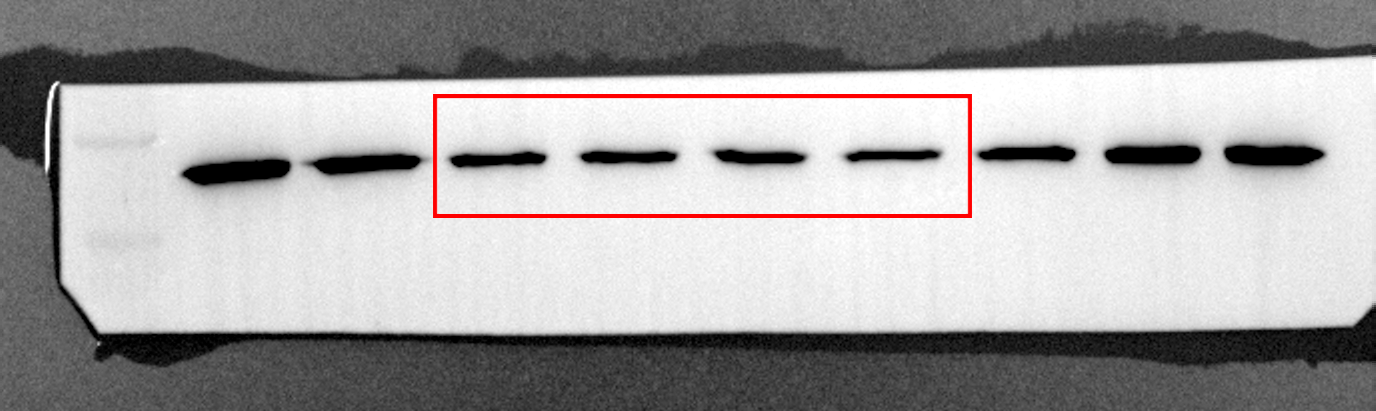

Supplement: S2 File — Underlying image data for Figures 1-9, S1, and S4. (ZIP) [file ppat.1013059.s002.zip › S2 File/Figure8-Detailed raw data/Figure8E/Figure8E-Tubulin.tif]

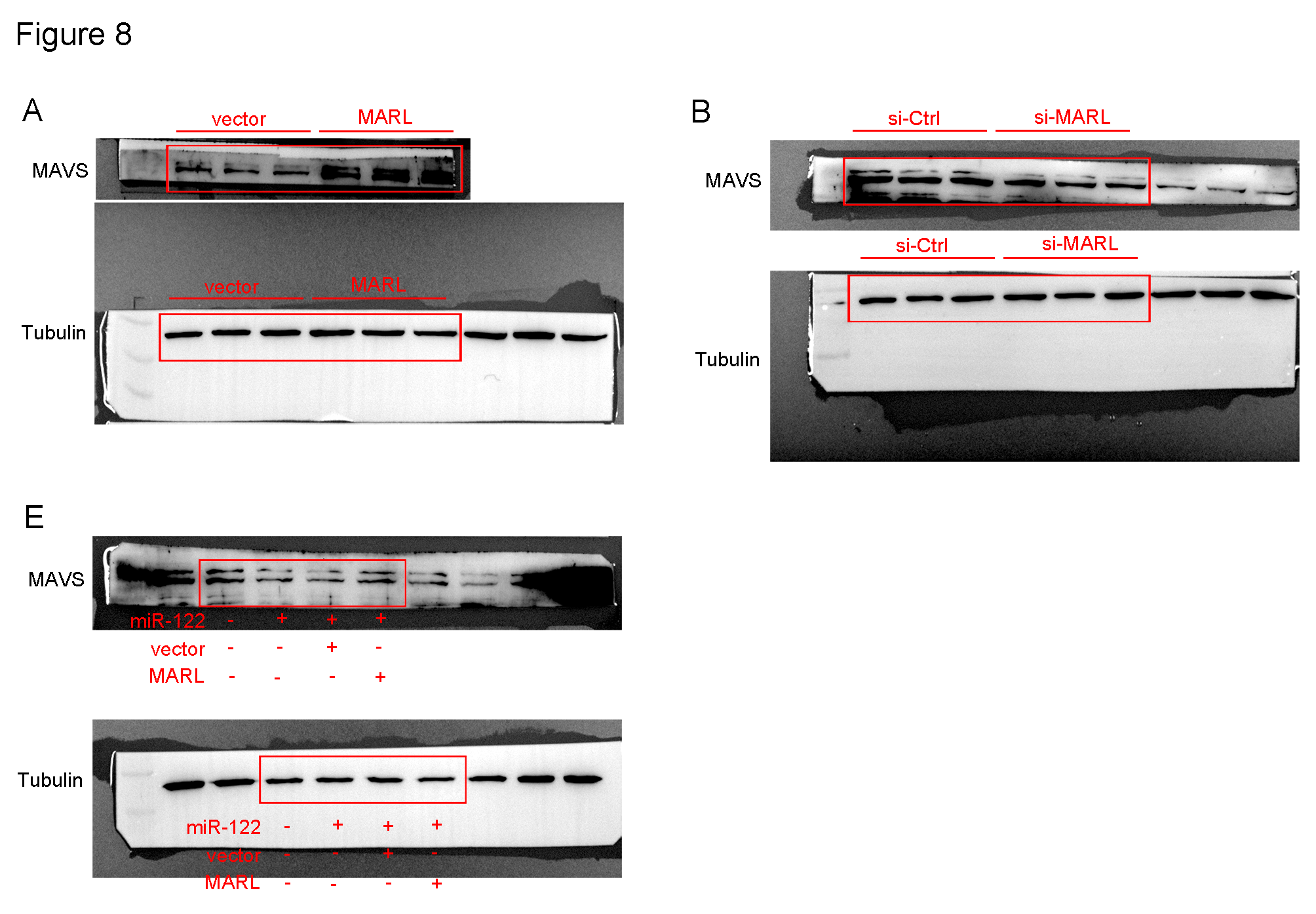

Supplement: S2 File — Underlying image data for Figures 1-9, S1, and S4. (ZIP) [file ppat.1013059.s002.zip › S2 File/Figure8.tif]

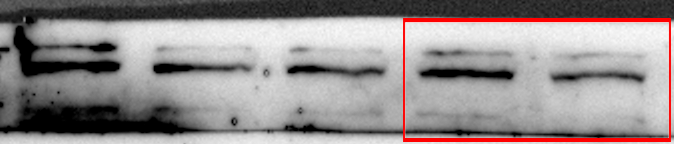

Supplement: S2 File — Underlying image data for Figures 1-9, S1, and S4. (ZIP) [file ppat.1013059.s002.zip › S2 File/Figure9-Detailed raw data/Figure9C/Figure9C-MAVS.tif]

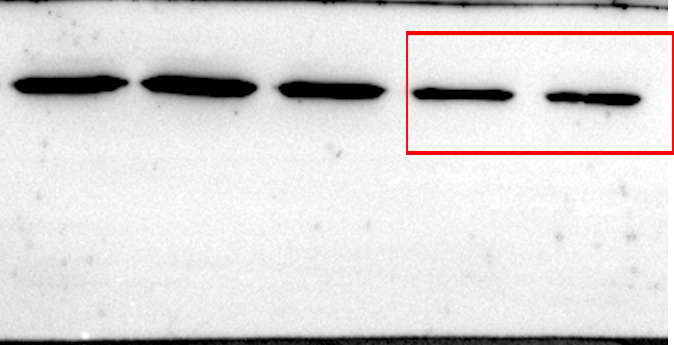

Supplement: S2 File — Underlying image data for Figures 1-9, S1, and S4. (ZIP) [file ppat.1013059.s002.zip › S2 File/Figure9-Detailed raw data/Figure9C/Figure9C-Tubulin.tif]

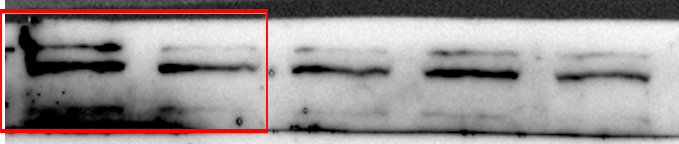

Supplement: S2 File — Underlying image data for Figures 1-9, S1, and S4. (ZIP) [file ppat.1013059.s002.zip › S2 File/Figure9-Detailed raw data/Figure9D/Figure9D-MAVS.tif]

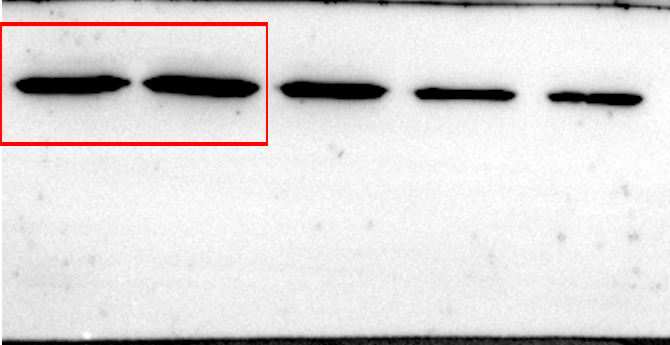

Supplement: S2 File — Underlying image data for Figures 1-9, S1, and S4. (ZIP) [file ppat.1013059.s002.zip › S2 File/Figure9-Detailed raw data/Figure9D/Figure9D-Tubulin.tif]

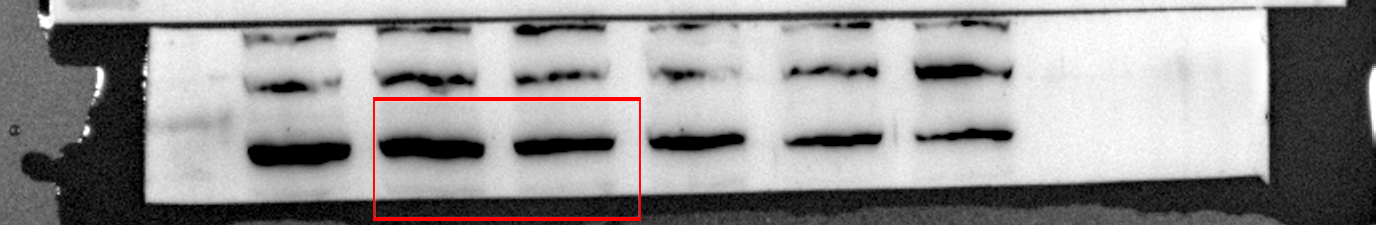

Supplement: S2 File — Underlying image data for Figures 1-9, S1, and S4. (ZIP) [file ppat.1013059.s002.zip › S2 File/Figure9-Detailed raw data/Figure9E/Figure9E-MAVS.tif]

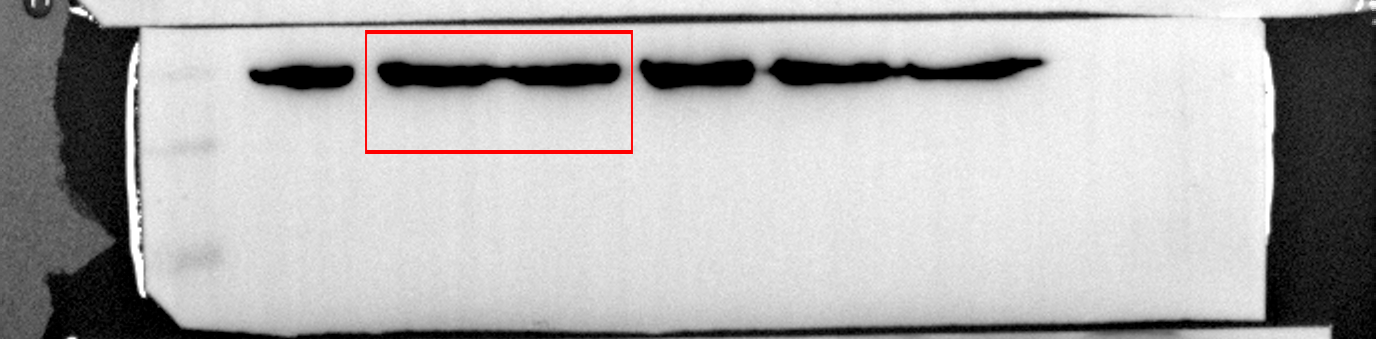

Supplement: S2 File — Underlying image data for Figures 1-9, S1, and S4. (ZIP) [file ppat.1013059.s002.zip › S2 File/Figure9-Detailed raw data/Figure9E/Figure9E-Tubulin.tif]

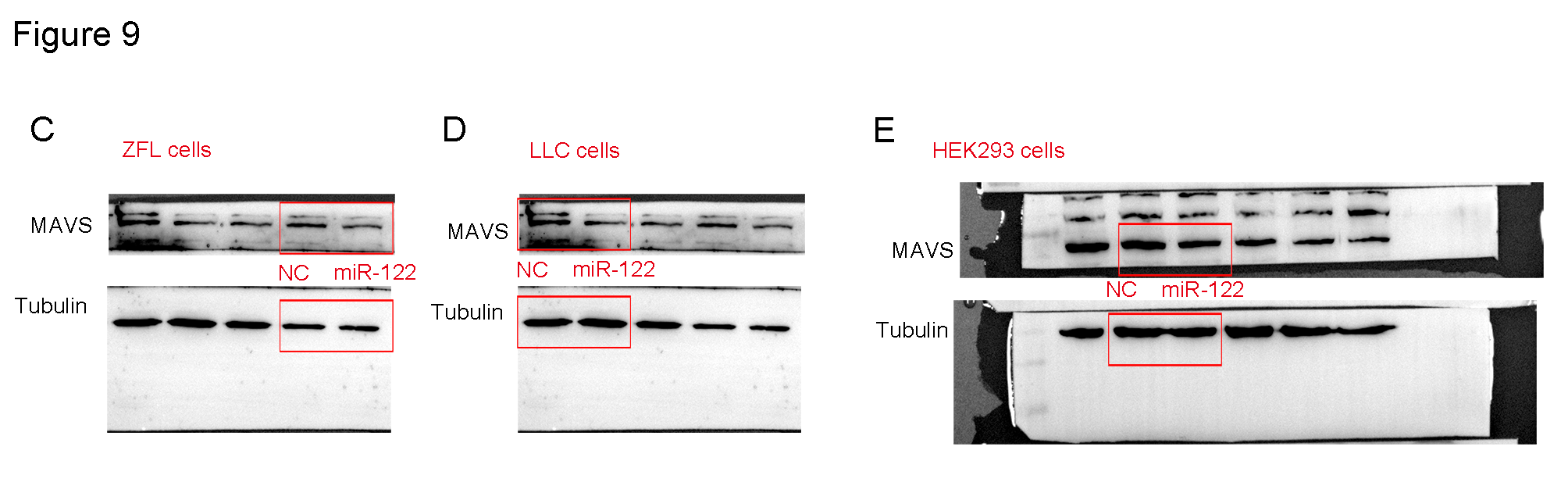

Supplement: S2 File — Underlying image data for Figures 1-9, S1, and S4. (ZIP) [file ppat.1013059.s002.zip › S2 File/Figure9.tif]

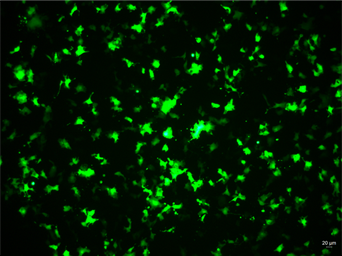

Supplement: S2 File — Underlying image data for Figures 1-9, S1, and S4. (ZIP) [file ppat.1013059.s002.zip › S2 File/FigureS1-Detailed raw data/FigureS1A/FigureS1A-3'UTR-mut-miR-122.tif]

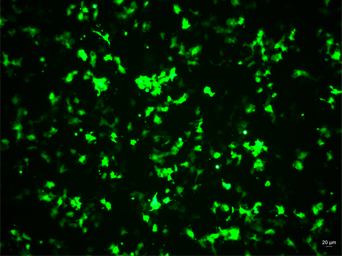

Supplement: S2 File — Underlying image data for Figures 1-9, S1, and S4. (ZIP) [file ppat.1013059.s002.zip › S2 File/FigureS1-Detailed raw data/FigureS1A/FigureS1A-3'UTR-mut-NC.tif]

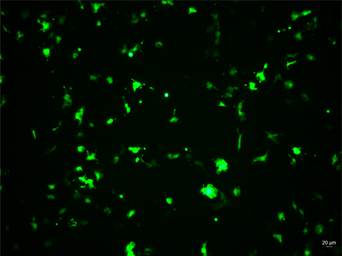

Supplement: S2 File — Underlying image data for Figures 1-9, S1, and S4. (ZIP) [file ppat.1013059.s002.zip › S2 File/FigureS1-Detailed raw data/FigureS1A/FigureS1A-3'UTR-wt-miR-122.tif]

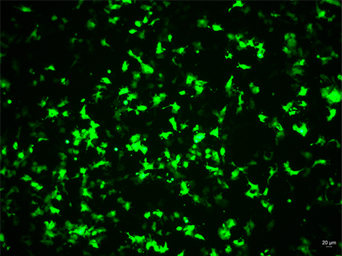

Supplement: S2 File — Underlying image data for Figures 1-9, S1, and S4. (ZIP) [file ppat.1013059.s002.zip › S2 File/FigureS1-Detailed raw data/FigureS1A/FigureS1A-3'UTR-wt-NC.tif]

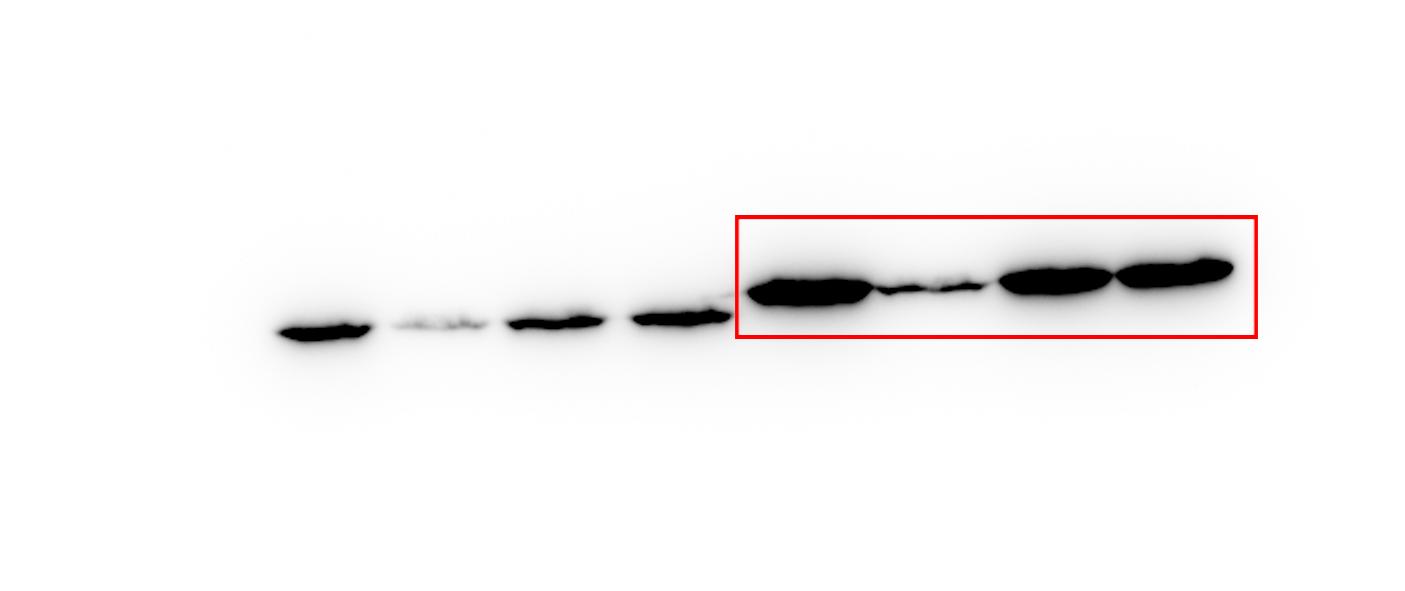

Supplement: S2 File — Underlying image data for Figures 1-9, S1, and S4. (ZIP) [file ppat.1013059.s002.zip › S2 File/FigureS1-Detailed raw data/FigureS1D/FigureS1D-GFP.tif]

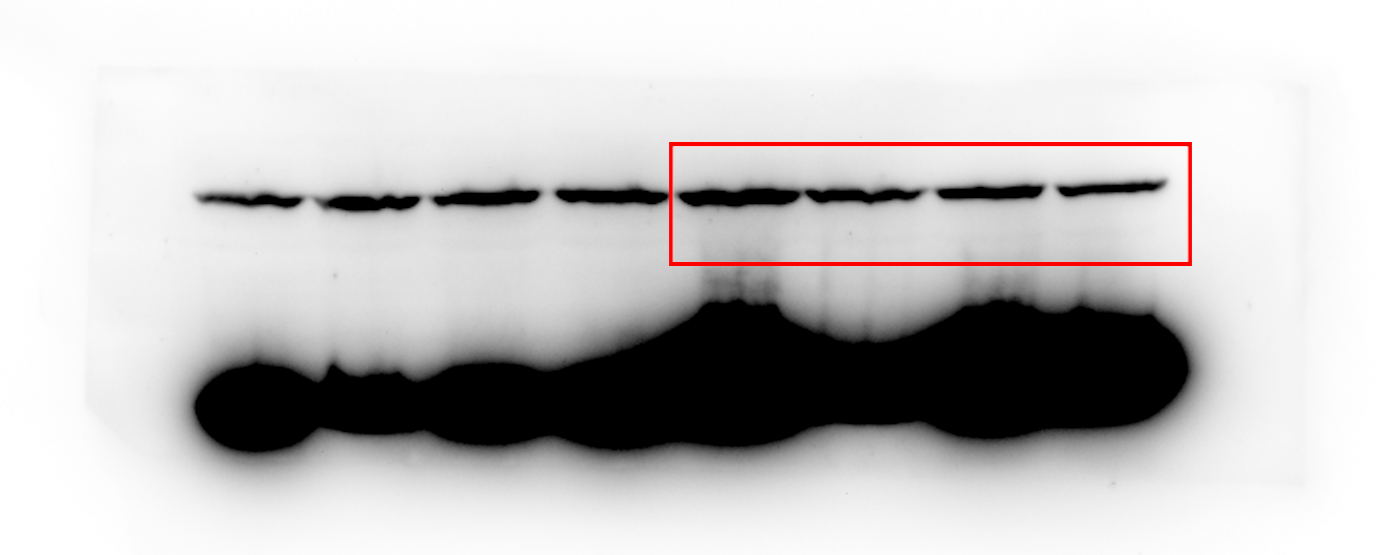

Supplement: S2 File — Underlying image data for Figures 1-9, S1, and S4. (ZIP) [file ppat.1013059.s002.zip › S2 File/FigureS1-Detailed raw data/FigureS1D/FigureS1D-Tubulin.tif]

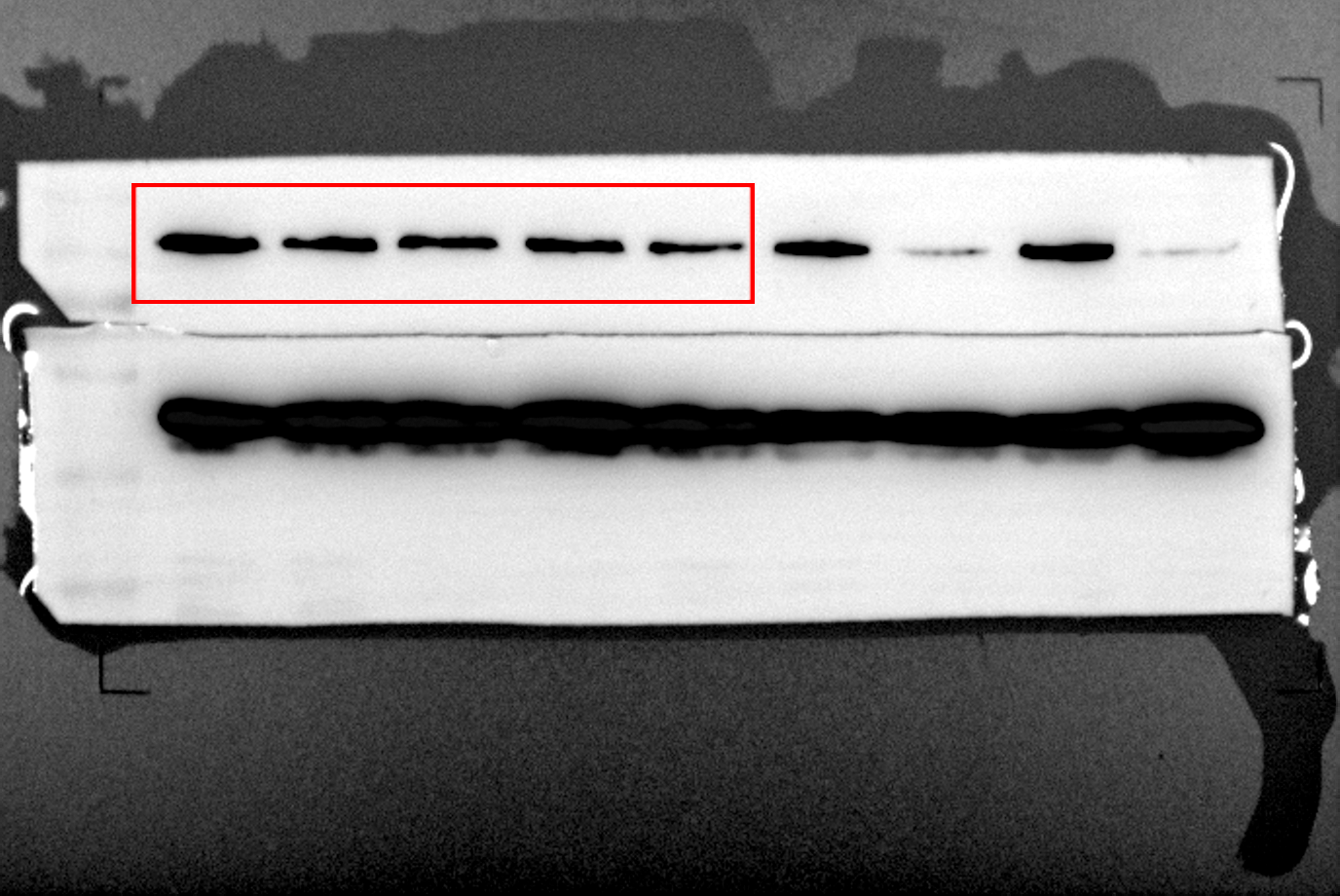

Supplement: S2 File — Underlying image data for Figures 1-9, S1, and S4. (ZIP) [file ppat.1013059.s002.zip › S2 File/FigureS1-Detailed raw data/FigureS1G/FigureS1G-Flag.tif]

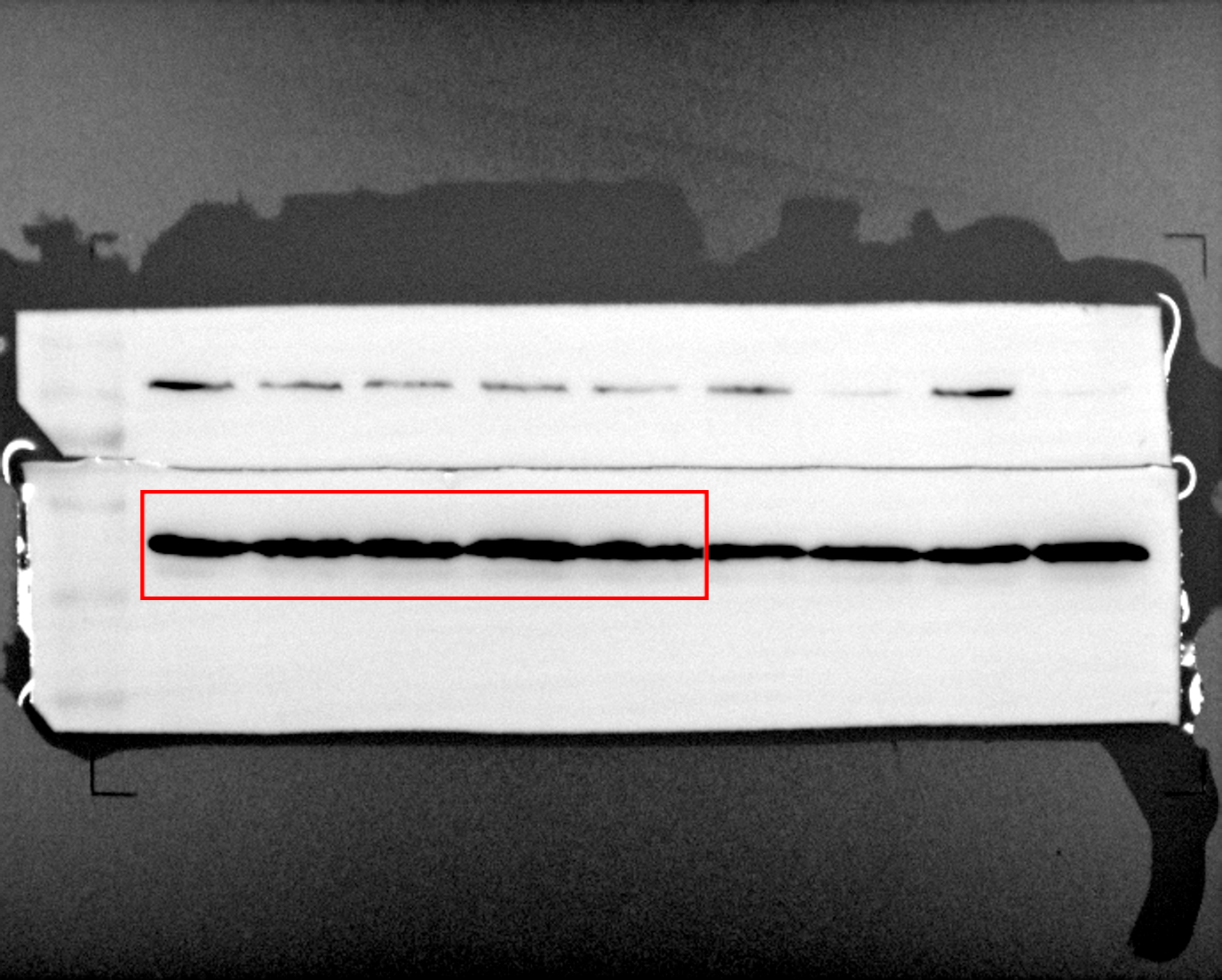

Supplement: S2 File — Underlying image data for Figures 1-9, S1, and S4. (ZIP) [file ppat.1013059.s002.zip › S2 File/FigureS1-Detailed raw data/FigureS1G/FigureS1G-Tubulin.tif]

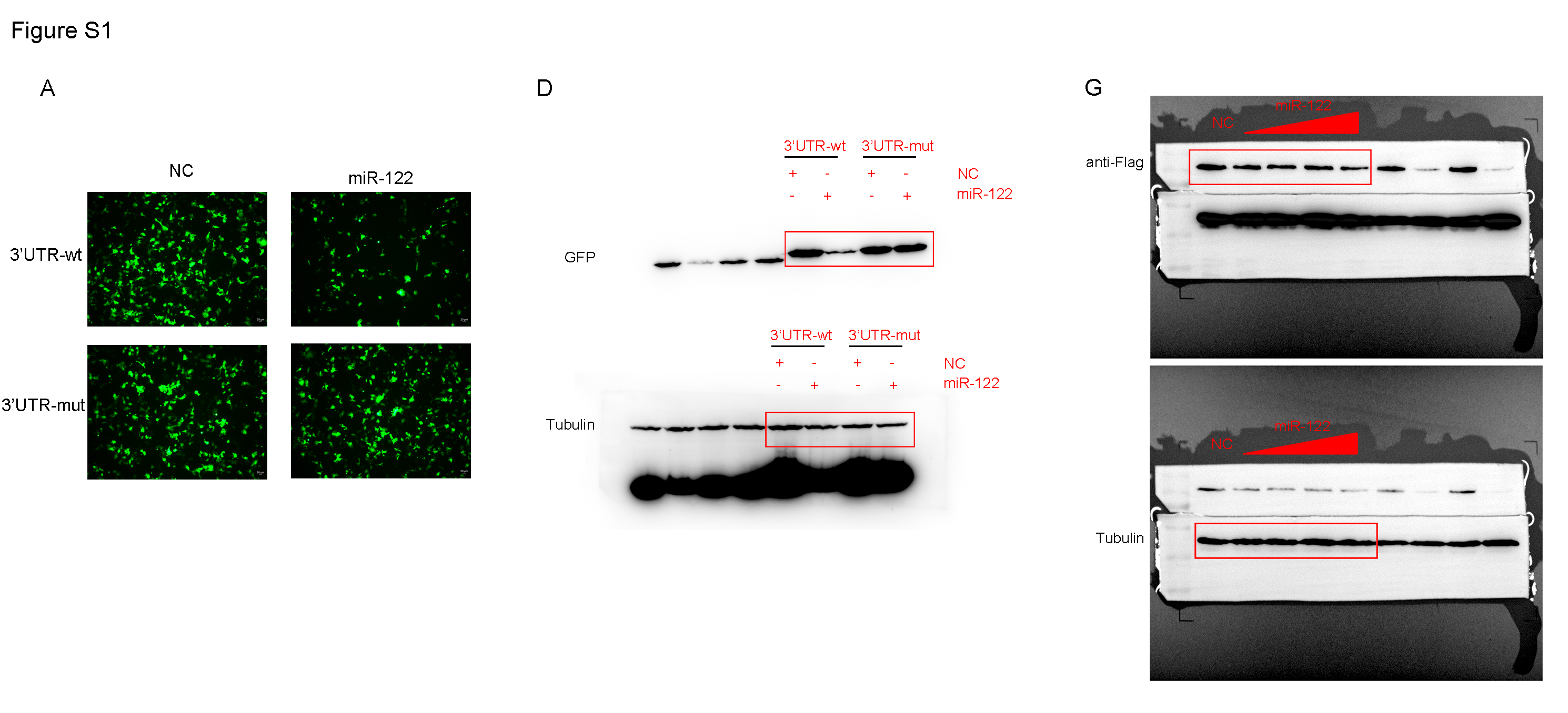

Supplement: S2 File — Underlying image data for Figures 1-9, S1, and S4. (ZIP) [file ppat.1013059.s002.zip › S2 File/FigureS1.tif]

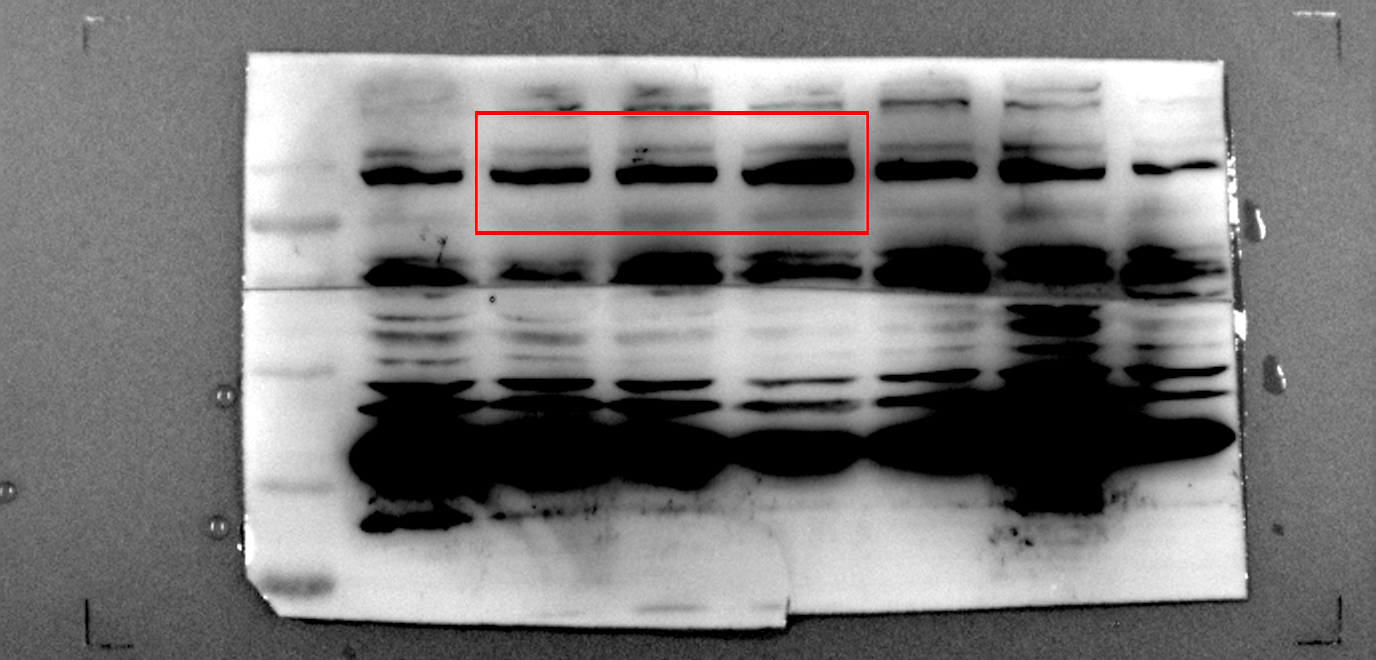

Supplement: S2 File — Underlying image data for Figures 1-9, S1, and S4. (ZIP) [file ppat.1013059.s002.zip › S2 File/FigureS4-Detailed raw data/FigureS4B/FigureS4B-MAVS.tif]

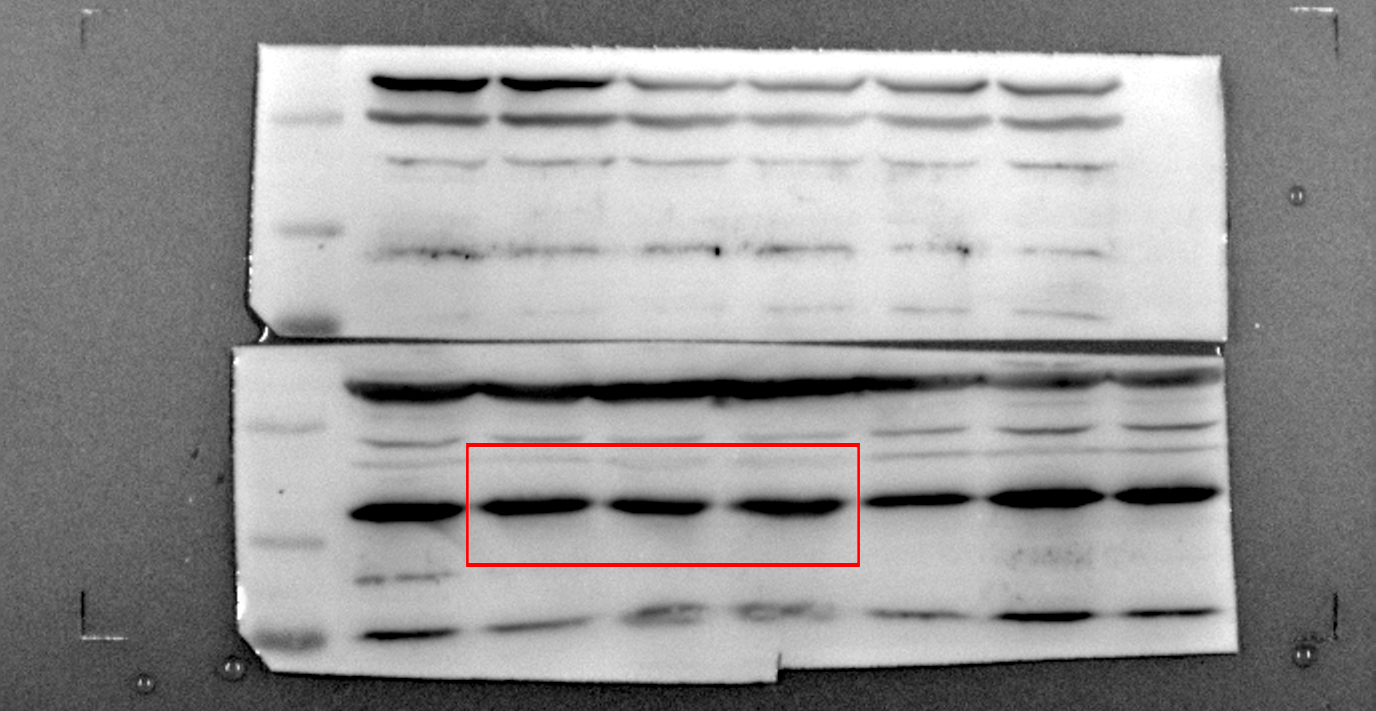

Supplement: S2 File — Underlying image data for Figures 1-9, S1, and S4. (ZIP) [file ppat.1013059.s002.zip › S2 File/FigureS4-Detailed raw data/FigureS4B/FigureS4B-Tubulin.tif]

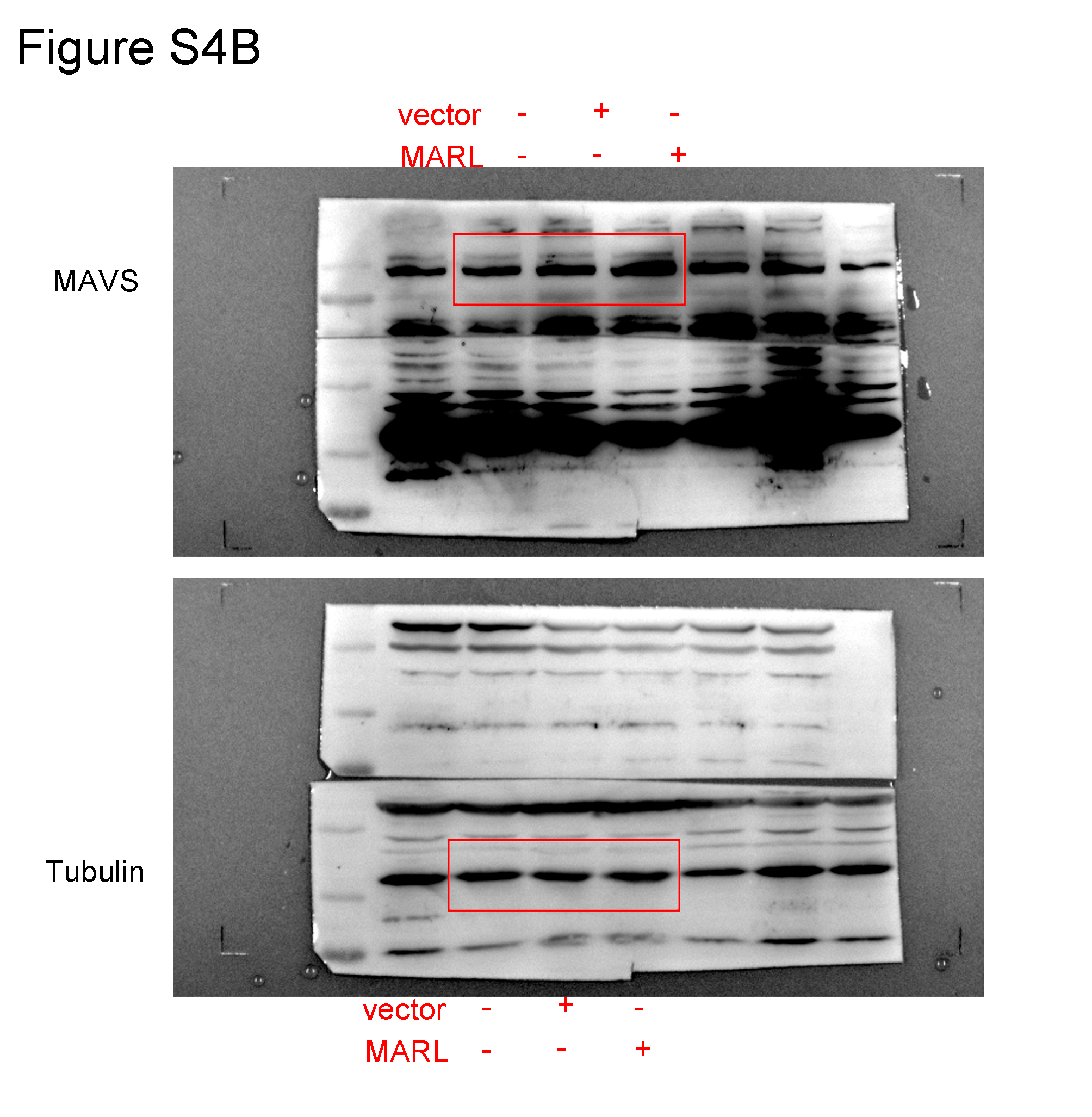

Supplement: S2 File — Underlying image data for Figures 1-9, S1, and S4. (ZIP) [file ppat.1013059.s002.zip › S2 File/FigureS4.tif]
